# Electroreductive coupling of 2-acylbenzoates with α,β-unsaturated carbonyl compounds: density functional theory study on product selectivity

**DOI:** 10.3762/bjoc.18.95

**Published:** 2022-08-02

**Authors:** Naoki Kise, Toshihiko Sakurai

**Affiliations:** 1 Department of Chemistry and Biotechnology, Graduate School of Engineering, Tottori University, 4-101, Koyama-cho Minami, Tottori 680-8552, Japan

**Keywords:** 2-acylbenzoates, chlorotrimethylsilane, 3-(3-cyanoethyl)phthalides, 2-cyanonaphthalen-1-ols, electroductive coupling

## Abstract

The electroreductive coupling of 2-acylbenzoates with acrylonitrile in the presence of TMSCl and successive treatment with 1 M HCl gave 2-cyanonaphthalen-1-ols or 3-(3-cyanoethyl)phthalides. On the other hand, the reaction of 2-acylbenzoates with methyl vinyl ketone under the same conditions produced 3-(3-oxobutyl)phthalides as the sole products. What determines the product selectivity was studied using DFT calculations.

## Introduction

**Scheme 1:**
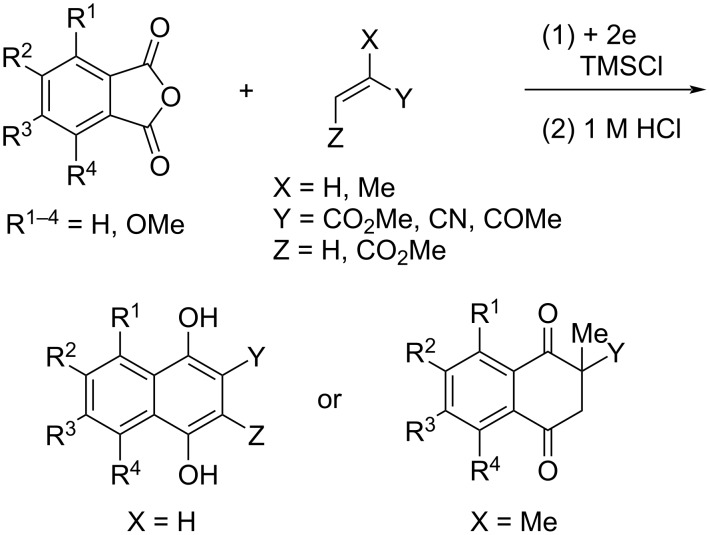
Electroreductive coupling of phthalic anhydrides with α,β-unsaturated carbonyl compounds and subsequent treatment with 1 M HCl (previous work).

**Scheme 2:**
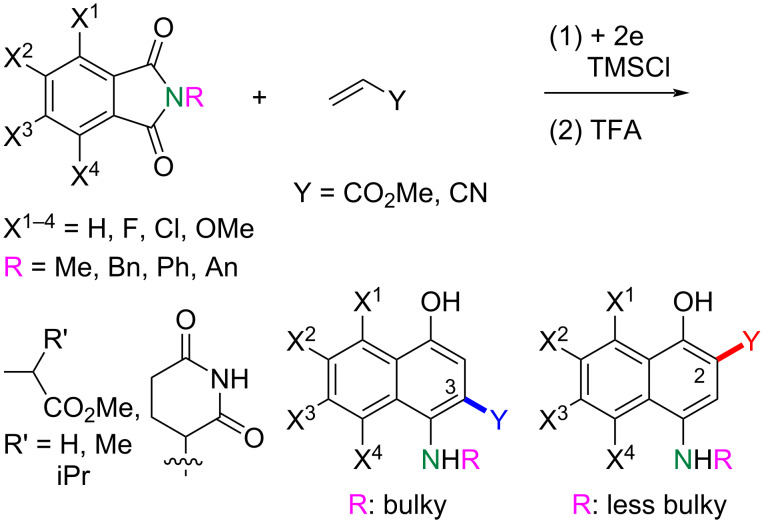
Electroreductive coupling of phthalimides with α,β-unsaturated carbonyl compounds and subsequent treatment with TFA (previous work).

**Scheme 3:**
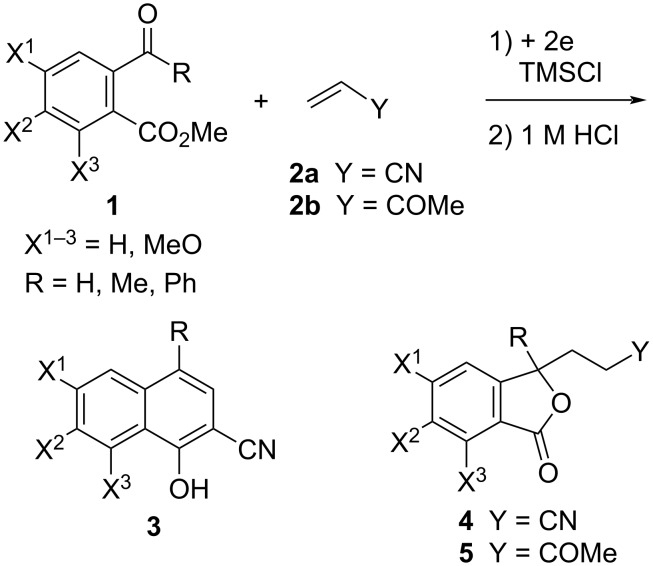
Electroreductive coupling of 2-acylbenzoates with α,β-unsaturated carbonyl compounds and subsequent treatment with 1 M HCl (this work).

The electroreductive coupling between carbon–heteroatom and carbon–carbon double bonds is one of the promising methods for carbon–carbon bond formation [1–4]. Recently, we reported the electroreductive coupling of phthalic anhydrides with α,β-unsaturated carbonyl compounds in the presence of chlorotrimethylsilane (TMSCl) and subsequent treatment with 1 M HCl to give 1,4-dihydroxynaphthalenes and 2-methyl-2,3-dihydronaphthalene-1,4-diones (Scheme 1) [5]. In addition, we disclosed that the electroreduction of phthalimides with α,β-unsaturated carbonyl compounds under the same conditions and subsequent treatment with trifluoroacetic acid (TFA) produced 3- and 2-substituted 4-aminonaphthalen-1-ols (Scheme 2) [6]. In this context, we report here that the electroreduction of *o*-acylbenzoates **1** with acrylonitrile (**2a**) in the presence of TMSCl and subsequent treatment with 1 M HCl gives 2-cyanonaphthalen-1-ols **3** or 3-(3-cyanoethyl)phthalides **4** (Scheme 3). The product selectivity depends on the position of the methoxy substituents on the aromatic ring in substrate **1**. On the other hand, 3-(3-oxobutyl)phthalides **5** are obtained by the reaction of compound **1** with methyl vinyl ketone (**2b**) as the sole products (Scheme 3). The synthesis of naththalene-1-ols [7–9] and 3-substituted phthalides [10–16] is attracting much attention, since bioactive compounds possessing these structures are known. This method has the potential to be applied to synthesize bioactive 2-cyanonaphthalen-1-ols [8–9] and 3-substituted phthalides [12–16]. The reaction mechanisms of the electroreductive coupling of **1** with **2** and subsequent rearrangement to **3** are also discussed. In particular, the latter mechanism was studied using density functional theory (DFT) calculations and it was suggested that the Δ*G* for the cyclization step of an intermediate enolate anion determines the product selectivity.

## Results and Discussion

**Scheme 4:**
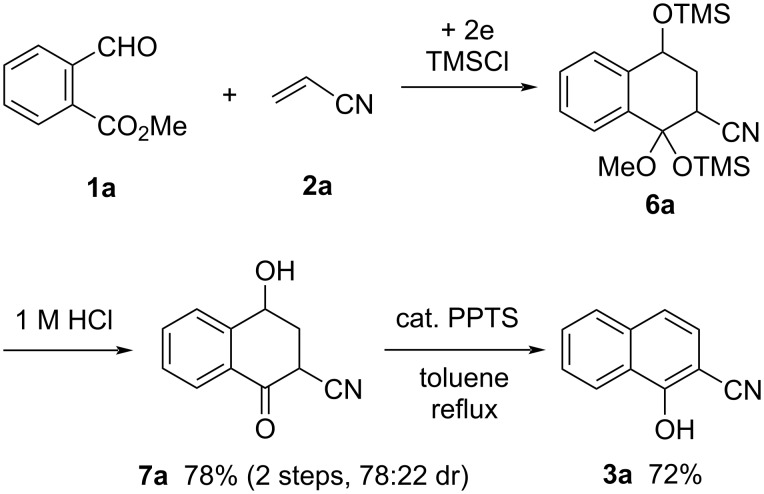
Electroreductive coupling of **1a** with **2a** and subsequent transformation to 2-cyanonaphthalene-1-ol (**3a**).

The electroreduction of methyl 2-formylbenzoate (**1a**) with acrylonitrile (**2a**) was carried out in 0.3 M Bu_4_NClO_4_/THF in the presence of TMSCl at 0.1 A (2.5 F/mol). From the crude product, cyclized product **6a** was obtained by column chromatography as a complex mixture of stereoisomers. Since compound **6a** could not be purified, it was treated with 1 M HCl/dioxane 1:1 at 25 °C for 1 h to give desilylated alcohol **7a** in 78% yield (2 steps) as a mixture of two diastereomers (78:22 dr). Dehydration of compound **7a** in refluxing toluene in the presence of cat. PPTS produced 2-cyanonaphthalene-1-ol (**3a**) in 72% yield (Scheme 4).

Next, the crude products of the electroreduction of methyl 2-acylbenzoates **1a**–**h** with **2a** were successively treated with 1 M HCl/dioxane 1:1 at 25 °C for 1 h and the results are summarized in Table 1. Dehydrated 2-cyanonaphthalene-1-ols **3b–d**,**g** were obtained only by treatment with 1 M HCl without dehydration in refluxing cat. PPTS/toluene (Table 1, entries 2–4 and 7). From 5,6-dimethoxy substrate **1d**, phthalide **4d** was also formed together with naphthol **3d** (Table 1, entry 4). In contrast, phthalides **4e** and **4f** were the sole products in the reactions of 6-methoxy and 4,5,6-trimethoxy substrates **1e** and **1f** (Table 1, entries 5 and 6). In the reaction of methyl 2-benzoylbenzoate (**1h**), the reduced product, 3-phenylphthalide (**i**), was formed mainly in 42% yield accompanied by phthalide **4h** in 24% yield (Table 1, entry 8).

**Table 1:** Electroreductive coupling of **1a–h** with **2a** and subsequent treatment with 1 M HCl.

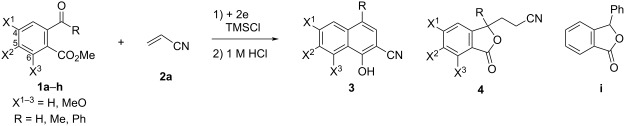						

Entry	**1**	R	X^1^	X^2^	X^3^	% Yield^a^

1	**1a**	H	H	H	H	**3a**, 56^b^
2	**1b**	H	H	MeO	H	**3b**, 71
3	**1c**	H	MeO	MeO	H	**3c**, 62
4	**1d**	H	H	MeO	MeO	**3d**, 36 **4d**, 26
5	**1e**	H	H	H	MeO	**4e**, 48
6	**1f**	H	MeO	MeO	MeO	**4f**, 41
7	**1g**	Me	H	H	H	**3g**, 73^c^
8	**1h**	Ph	H	H	H	**4h**, 24^d^

^a^Isolated yields. ^b^After dehydration of **7a** by refluxing in cat. PPTS/toluene for 1h. ^c^The reaction time for treatment with 1 M HCl was extended to 10 h. ^d^3-Phenylphthalide (**i**) was obtained as main product (42% yield).

On the other hand, the electroreduction of **1a**–**h** with methyl vinyl ketone (**2b**) and subsequent treatment with 1 M HCl afforded phthalides **5a**–**h** in moderate to good yields and naphthalene-1-ols **3’** corresponding to cyclized products **3** were not formed at all (Table 2).

**Table 2:** Electroreductive coupling of **1a**–**h** with methyl vinyl ketone (**2b**) and subsequent treatment with 1 M HCl.

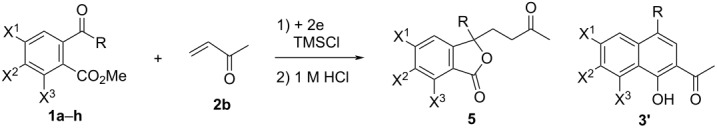						

Entry	**1**	R	X^1^	X^2^	X^3^	% Yield^a^

1	**1a**	H	H	H	H	**5a**, 85
2	**1b**	H	H	MeO	H	**5b**, 77
3	**1c**	H	MeO	MeO	H	**5c**, 88
4	**1d**	H	H	MeO	MeO	**5d**, 67
5	**1e**	H	H	H	MeO	**5e**, 66
6	**1f**	H	MeO	MeO	MeO	**5f**, 73
7	**1g**	Me	H	H	H	**5g**, 74
8	**1h**	Ph	H	H	H	**5h**, 74

^a^Isolated yields.

**Scheme 5:**
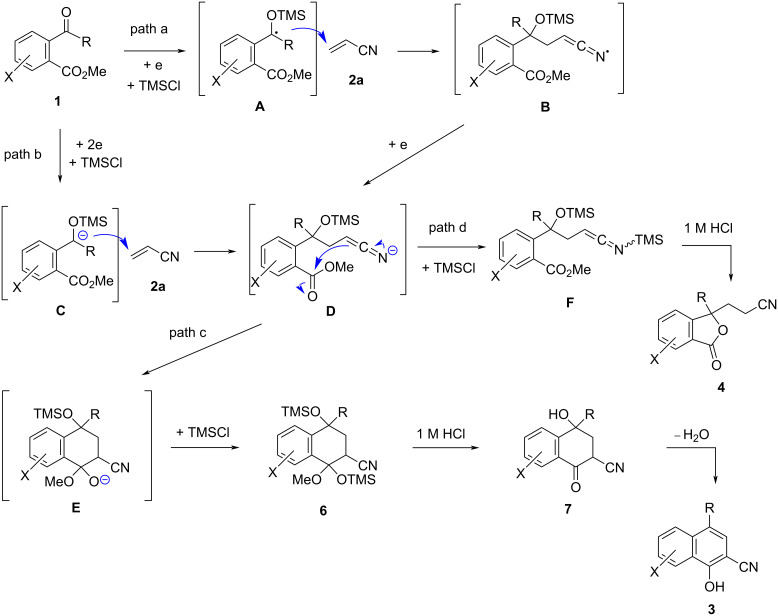
Presumed reaction mechanism of electroreductive coupling of **1** with **2a** and subsequent transformation to products **3** and **4**.

**Scheme 6:**
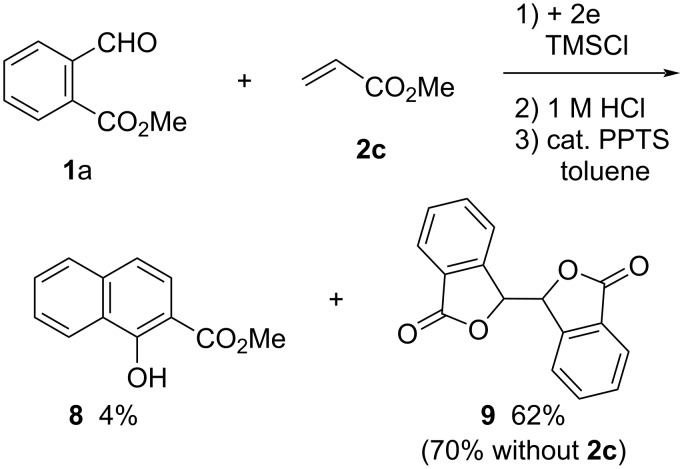
Electroreductive coupling of **1a** with **2c** and subsequent treatment with 1 M HCl.

The *E*_p_ values of substrates **1a**–**h** were observed to be in the range from −1.74 to −1.96 V versus SCE by cyclic voltammetry (Table 3) and acceptors **2** revealed no reduction peaks from 0 to −2.00 V vs SCE [5–6]. Therefore, this electroreductive coupling is initiated by the reduction of compounds **1**. There are two possible reaction mechanisms for the reductive coupling of **1** with **2a** as illustrated in Scheme 5. The first one is a radical addition of *O*-trimethylsilyl radical **A**, which is formed by a one-electron reduction of **1** and subsequent *O*-trimethylsilylation, to **2a** and a following one-electron reduction of the resultant radical **B** to give enolate anion **D** (path a). The second one is an anionic addition of an *O*-trimethylsilyl anion **C**, which is formed by a two-electron reduction of substrate **1** and *O*-trimethylsillylation, to **2a** (path b). Unlike the two reactions previously reported by us that are presumed to proceed with the addition of an anion species (Scheme 1 and Scheme 2) [5–6], methyl acrylate (**2c**) is much less reactive as an acceptor in this reaction as shown in Scheme 6. The main product in this case was the same dimeric phthalide **9** as the product without the acceptor. These results suggest that this reaction proceeds with the radical addition of **A** to form anion **D** (path a). Next, the intramolecular addition of the anion **D** and subsequent *O*-trimethylsilylation of the resultant **E** produces intermediate **6** (path c). Desilylation of **6** with 1 M HCl and following dehydration of **7** affords product **3**. On the other hand, *O*-trimethylsilylation of anion **D** forms *N*-(trimethylsilyl)ethenimine **F** and subsequent treatment with 1 M HCl produces phthalide **4** through desilylation and following lactonization of **F** (path d).

**Table 3:** *E*_p_ values of **1a**–**h** derived from CV.

**1**	*E*_p_^a^	**1**	*E*_p_^a^

**1a**	−1.74	**1e**	−1.90
**1b**	−1.86	**1f**	−1.86
**1c**	−1.74	**1g**	−1.96
**1d**	−1.92	**1h**	−1.92

^a^First reduction peak (volts vs SCE) in CV of a 3 mM solution in 0.03 M TBAP/DMF at a Pt cathode at 0.1 V/s and 25 °C.

As can be seen from Scheme 5, the cyclization of **D** to **E** is the key step for the formation of compound **6**. Therefore, we calculated the intermediates (**D** and **E**) and transition states (**D–E TS**) for this step using the DFT method at the B3LYP/6-311+(2d,p)/IEFPCM(THF) level of theory (Supporting Information File 1). From the calculation results for the reactions of **1a**–**h** with **2a** summarized in Table 4, it was found that the ratio of **D**:**E** calculated from the free energy difference between **D** and **E** (∆*G*) and the product ratio of **4**:**3** from the experimental results (Table 1, entries 1–6) were in good agreement. Therefore, it is presumed that whether the cyclization from **D** to **E** proceeds is thermodynamically controlled. Namely, when ∆*G* is large and negative, product **3** is selectively formed (Table 4, entries 1–3), and conversely, when ∆*G* is large and positive, product **4** is selectively produced (Table 4, entries 5 and 6). On the other hand, when ∆*G* is close to zero, both products **3** and **4** are generated simultaneously (Table 4, entry 4). These results suggest that the substitution of the methoxy group at the 6-position tends to suppress the cyclization of **D** to **E** (Table 4, entries 4–6), since its electron-donating property reduces the electrophilicity of the ester carbonyl group. In contrast, the substitution of the methoxy group at the 5-position tends to promote the cyclization of **D**, owing to its electron-withdrawing property (Table 4, entries 2 and 3).

**Table 4:** Calculations of activation energies (∆*G*^‡^) and energy differences (∆*G*) from **Dx** to **Ex**.

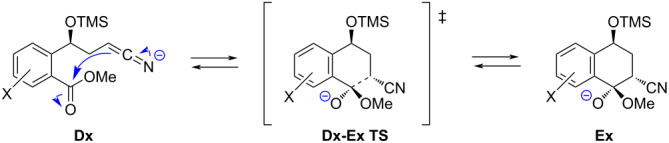					

		∆*G*^‡^	∆*G*	**D**:**E**	**4**:**3**
Entry	**Dx**	(kcal/mol)^a^		(calcd)^b^	(exp)^c^

1	**Da**	6.73	−1.22	11:89	<1:99
2	**Db**	5.86	−2.38	2:98	<1:99
3	**Dc**	4.94	−2.17	3:97	<1:99
4	**Dd**	7.82	0.33	36:64	42:58
5	**De**	9.16	1.37	91:9	>99:1
6	**Df**	9.13	2.55	99:1	>99:1

^a^Calculated at the B3LYP/6-311+G(2d,p)/ICFPCM(THF) level of theory at 25 °C. ^b^Calculated from ∆*G* on the basis of the Maxwell–Boltzmann distribution law at 25 °C. ^c^Data from entries 1–6 in Table 1.

From the calculation results for the reaction of **1a** with **2b** (Table 5), it is understood that the cyclization from **D'a** to **E'a** hardly occurs because it shows a relatively large positive Δ*G*.

**Table 5:** Calculations of ∆*G* from **D’a** to **E’a**.

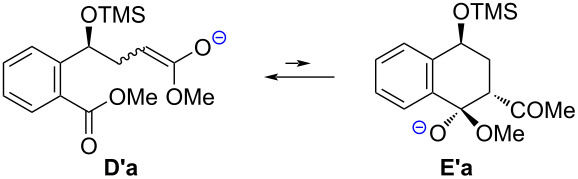			

	∆*G*	**D’a**:**E’a**	**5**:**3’**
**D’a**	(kcal/mol)^a^	(calcd)^b^	(exp)^c^

*E*-form	6.90	100:0	>99:1
*Z*-form	8.13	100:0	>99:1

^a^Calculated at the B3LYP/6-311+G(2d,p)/ICFPCM(THF) level of theory at 25 °C. ^b^Calculated from ∆*G* on the basis of the Maxwell–Boltzmann distribution law at 25 °C. ^c^Data from entry 1 in Table 2.

## Conclusion

The electroreduction of *o*-acylbenzoates **1** with acrylonitrile (**2a**) in the presence of TMSCl and subsequent treatment with 1 M HCl gave 2-cyanonaphthalen-1-ols **3** and 3-(3-cyanoethyl)phthalides **4**. Which product was preferentially produced is determined by the position of the methoxy group on the aromatic ring of the substrate **1**. Using the same method, 3-(3-oxobutyl)phthalides **5** were produced as the sole products by the reaction of **1** with methyl vinyl ketone (**2b**). It was found by DFT calculations for the cyclization step of the intermediate enolate anions that the product selectivity was in good agreement with the free energy differences (∆*G*) in the cyclization step.

## Experimental

**General information.** The ^1^H NMR (500 MHz) and ^13^C NMR (125 MHz) spectra were measured on a JEOL GMX-500 spectrometer with tetramethylsilane (TMS) or the residual signals of protonated solvents as an internal standard: CDCl_3_ (δ = 77.0 in ^13^C NMR). IR spectra were recorded on a Shimadzu IRAffinity-1 infrared spectrometer. HRMS were measured on a Thermo Scientic Exactive FTMS spectrometer. Melting points were uncorrected. Column chromatography was performed on silica gel 60. THF was distilled from sodium benzophenone ketyl radical. TMSCl, TEA, and DMF were distilled from CaH_2_.

**Starting materials.** Methyl 2-formylbenzoate (**1a**) and methyl 2-benzoylbenzoate (**1h**) were purchased from Tokyo Chemical Industry Corporation. Methyl 2-acetylbenzoate (**1g**) [17] was prepared from commercially available 2-acetylbenzoic acids (Tokyo Chemical Industry Corporation) by usual esterification using MeI, K_2_CO_3_/acetone at 25 °C for 12 h. Methoxy-substituted 2-formylbenzoates **1b** [18], **1c** [19], **1d** [20], **1e** [21], and **1f** [22] were prepared according to the reported methods.

**Typical procedures for electroreduction in the presence of TMSCl** (Table 1, entry 1). A 0.3 M solution of Bu_4_NClO_4_ in THF (15 mL) was placed in the cathodic chamber of a divided cell (40 mL beaker, 3 cm diameter, 6 cm height) equipped with a platinum cathode (5 × 5 cm^2^), a platinum anode (2 × 1 cm^2^), and a ceramic cylindrical diaphragm (1.5 cm diameter). A 0.3 M solution of Bu_4_NClO_4_ in DMF (4 mL) was placed in the anodic chamber (inside the diaphragm). Methyl 2-formylbenzoate (**1a**, 161 mg, 1.0 mmol), acrylonitrile (**2a**, 258 mg, 2.5 mmol), TMSCl (0.64 mL, 5 mmol), and TEA (0.14 mL, 1 mmol) were added to the cathodic chamber. After 250 C of electricity (2.5 F/mol) have passed at a constant current of 100 mA at room temperature under a nitrogen atmosphere (42 min), the catholyte was evaporated in vacuo. The residue was dissolved in diethyl ether (20 mL) and insoluble solid was filtered off. After removal of the solvent in vacuo, the residue was dissolved in 1 M HCl (5 mL)/1,4-dioxane (5 mL) and the solution was stirred at 30 °C for 1 h. The mixture was diluted with sat. aqueous NaCl solution (20 mL) and water (20 mL), and then extracted with ethyl acetate (3 × 20 mL). The organic layer was washed with sat. aqueous NaCl solution, dried over MgSO_4_, and filtered. After removal of the solvent in vacuo, the residue was purified by column chromatography on silica gel (hexanes/EtOAc) to give 146 mg of **7a** [23] (78% yield) as a mixture of two diastereomers (78:22 dr). A solution of **7a** (146 mg) and PPTS (10 mg) in toluene (10 mL) was refluxed using the Dean–Stark apparatus under nitrogen atmosphere for 1 h. After removal of the solvent in vacuo, the residue was purified by column chromatography on silica gel (hexanes/EtOAc) to give 95 mg of **3a** [8,23] (56% yield in two steps).

## Supporting Information

File 1 Characterization data for compounds, copies of ^1^H and ^13^C NMR spectra, X-ray crystallographic data (ORTEP) of **3b**, CV data of compounds **1a**–**h**, and DFT calculation data for cyclization of enolate anions.

File 2 Cif for **3b**.
