# Supplementary material for: Electroreductive coupling of 2-acylbenzoates with α,β-unsaturated carbonyl compounds: density functional theory study on product selectivity
Source: Beilstein J Org Chem. 2022 Aug 2;18:956–62. doi: 10.3762/bjoc.18.95 (PMC9359203; doi:10.3762/bjoc.18.95)
Supplement: File 1 — Characterization data for compounds, copies of 1H and 13C NMR spectra, X-ray crystallographic data (ORTEP) of 3b, CV data of compounds 1a–h, and DFT calculation data for cyclization of enolate anions. [file Beilstein_J_Org_Chem-18-956-s001.pdf]

BEILSTEIN JOURNAL OF ORGANIC CHEMISTRY

Supporting Information

for

Electroreductive coupling of 2-acylbenzoates with α,β-unsaturated carbonyl compounds: density functional theory study on product selectivity

Naoki Kise and Toshihiko Sakurai

Beilstein J. Org. Chem. **2022,** *18,* 956–962. doi:10.3762/bjoc.18.95

Characterization data for compounds, copies of ^{1}H and ^{13}C NMR spectra, X-ray crystallographic data (ORTEP) of 3b, CV data of compounds 1a–h, and DFT calculation data for cyclization of enolate anions

License and Terms: This is a supporting information file under the terms of the Creative Commons Attribution License (https://creativecommons.org/licenses/by/4.0). Please note that the reuse, redistribution and reproduction in particular requires that the author(s) and source are credited and that individual graphics may be subject to special legal provisions.
The license is subject to the *Beilstein Journal of Organic Chemistry* terms and conditions: (https://www.beilstein-journals.org/bjoc/terms)

Table of contents

1. Characterization data for compounds S1–S5

2. ^{1}H and ^{13}C NMR spectra of compounds S6–S35

3. X-ray crystallographic data (ORTEP) of **3b** S36

4. CV data of **1a**–**h** S37

5. DFT calculation data for cyclization of enolate anions S38–S71

 1) Electroreductive coupling of **1a** with **2a** S39–S49

 2) Electroreductive coupling of **1b**–**f** with **2a** S50–S65

 3) Electroreductive coupling of **1a** with **2b** S66–S71

References S72

1. Characterization data for compounds

4-Hydroxy-1-oxo-1,2,3,4-tetrahydronaphthalene-2-carbonitrile (7a) (72:28 diastereomeric mixture) [1]

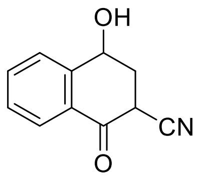

Yellow solid (146 mg, 78%); R_f 0.35 (1:5 hexanes/ethyl acetate); mp 126−128 °C (recrystallized from 2:1 hexanes/ethyl acetate, lit.[1] 116−118 °C); ^{1}H NMR ($CDCl_3$, 500 MHz) δ 8.11-8.06 (m, 1H), 7.77-7.65 (m, 1.28H), 7.54-7.44 (m, 1.72H), 5.12-5.04 (m, 1H), 4.42 (dd, 0.72H, J = 4.6, 12.2 Hz), 3.82 (dd, 0.28H, J = 4.3, 13.2 Hz), 2.90-2.84 (m, 0.28H), 2.77-2.71 (m, 0.72H), 2.70-2.62 (m, 0.72H), 2.58 (brs, 0.28H), 2.51-2.41 (m, 0.28H), 2.31 (brs, 0.72H); ^{13}C{^{1}H} NMR ($CDCl_3$/DMSO-d_6, 125 MHz) δ 188.1 (s), 187.5 (s), 146.6 (s), 143.3 (s), 134.8 (d), 134.6 (d), 129.1 (d), 128.9 (s), 128.6 (d), 128.5 (s), 127.6 (d), 127.2 (d), 127.1 (d), 126.4 (d), 117.1 (s), 116.3 (s), 65.5 (d), 64.3 (d), 39.1 (d), 36.3 (t), 35.5 (d), 34.9 (t).

1-Hydroxy-2-naphthonitrile (3a) [1,2]

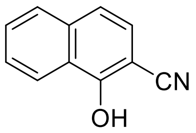

White solid (95 mg, 56%); R_f 0.6 (2:1 hexanes/ethyl acetate); mp 182−184 °C (recrystallized from 2:1 hexanes/ethyl acetate, lit.[1] 176−177 °C); ^{1}H NMR ($CDCl_3$, 500 MHz) δ 8.31 (d, 1H, J = 8.2 Hz), 7.80 (d, 1H, J = 8.2 Hz), 7.66-7.61 (m, 1H), 7.60-7.56 (m, 1H), 7.42 (d, 1H, J = 8.6 Hz), 7.36 (d, 1H, J = 8.6 Hz), 7.13 (brs, 1H); ^{13}C{^{1}H} NMR ($CDCl_3$/DMSO-d_6, 125 MHz) δ 157.8 (s), 136.5 (s), 129.7 (d), 127.8 (d), 126.8 (d), 125.2 (d), 123.9 (s), 123.0 (d), 121.2 (d), 117.2 (s), 91.9 (s).

1-Hydroxy-7-methoxy-2-naphthonitrile (3b)

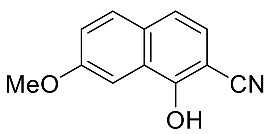

White solid (141 mg, 71%); R_f 0.5 (2:1 hexanes/ethyl acetate); mp 181−183 °C (recrystallized from 2:1 hexanes/ethyl acetate); IR (ATR) 3290 (br), 2231, 1629, 1605, 1580, 1514 cm^{-1}; ^{1}H NMR ($CDCl_3$, 500 MHz) δ 7.72 (d, 1H, J = 9.0 Hz), 7.55 (d, 1H, J = 2.6 Hz), 7.37 (d, 1H, J = 8.5 Hz), 7.29 (dd, 1H, J = 2.6 9.0 Hz), 7.25 (d, 1H, J = 8.5 Hz), 6.76 (brs, 1H), 3.96 (s, 3H); ^{13}C{^{1}H} NMR ($CDCl_3$, 125 MHz) δ 158.4 (s), 156.5 (s), 131.8 (s), 129.3 (d), 124.9 (s), 122.8 (d), 122.3 (d), 120.9 (d), 117.5 (s), 101.1 (d), 92.4 (s), 55.5 (q); HRMS (ESI) *m/z* [M + Na]$^+$ calcd. for $C_{12}H_9NO_2Na$ 222.0531, found 222.0531.

1-Hydroxy-6,7-dimethoxy-2-naphthonitrile (3c)

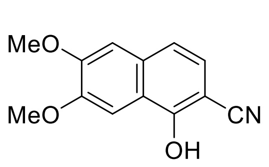

White solid (142 mg, 62%); R_f 0.55 (1:1 hexanes/ethyl acetate); mp 225−227 °C (recrystallized from 2:1 hexanes/ethyl acetate); IR (ATR) 3284 (br), 2231, 1625, 1584, 1517 cm^{-1}; ^{1}H NMR ($CDCl_3$, 500 MHz) δ 9.88 (brs, 1H), 7.66 (s, 1H), 7.26 (d, 1H, J = 8.5 Hz), 7.21 (d, 1H, J = 8.5 Hz), 7.06 (s, 1H), 4.01 (s, 6H); ^{13}C{^{1}H} NMR ($CDCl_3$, 125 MHz) δ 155.5 (s), 149.9 (s), 147.9 (s), 131.1 (s), 123.5 (d), 118.0 (s), 117.3 (d), 116.8 (s), 105.2 (d), 100.5 (d), 90.5 (s), 54.2 (q), 54.1 (q) ; HRMS (ESI) *m/z* [M + H]$^+$ calcd. for $C_{13}H_{12}NO_3$ 230.0817, found 230.0812.

1-Hydroxy-7,8-dimethoxy-2-naphthonitrile (3d)

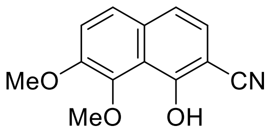

White solid (83 mg, 36%); *Rf* 0.5 (2:1 hexanes/ethyl acetate); mp 138−139 °C (recrystallized from 1:1 hexanes/ethyl acetate); IR (ATR) 3184 (br), 2219, 1610, 1587, 1516 cm^{-1}; ^{1}H NMR ($CDCl_3$, 500 MHz) δ 10.66 (brs, 1H), 7.58 (d, 1H, J = 9.0 Hz), 7.39 (d, 1H, J = 9.0 Hz), 7.27 (d, 1H, J = 8.6 Hz), 7.24 (d, 1H, J = 8.6 Hz), 4.10 (s, 3H), 4.02 (s, 3H); ^{13}C{^{1}H} NMR ($CDCl_3$, 125 MHz) δ 158.8 (s), 148.1 (s), 143.0 (s), 131.8 (s), 125.21 (d), 125.16 (d), 119.5 (d), 117.5 (d), 117.2 (s), 117.0 (s), 93.5 (s), 62.3 (q), 56.7 (q); HRMS (ESI) *m/z* [M + H]$^{+}$ calcd. for $C_{13}H_{12}NO_3$ 230.0817, found 230.0813.

3-(4,5-Dimethoxy-3-oxo-1,3-dihydroisobenzofuran-1-yl)propanenitrile (4d)

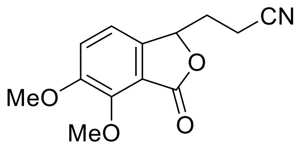

White solid (64 mg, 26%); *Rf* 0.55 (1:2 hexanes/ethyl acetate); mp 134−135 °C (recrystallized from 1:2 hexanes/ethyl acetate); IR (ATR) 2237, 1756, 1597 cm^{-1}; ^{1}H NMR ($CDCl_3$, 500 MHz) δ 7.27 (d, 1H, J = 8.2 Hz), 7.08 (d, 1H, J = 8.2 Hz), 5.46 (dd, 1H, J = 3.0, 9.0 Hz), 4.11 (s, 3H), 3.93 (s, 3H), 2.67-2.59 (m, 1H), 2.57-2.40 (m, 2H), 2.04-1.95 (m, 1H); ^{13}C{^{1}H} NMR ($CDCl_3$, 125 MHz) δ 167.1 (s), 152.8 (s), 148.2 (s), 140.6 (s), 119.4 (d), 118.5 (s), 117.7 (s), 116.2 (d), 77.2 (d), 62.2 (q), 56.6 (q), 30.8 (t), 13.2 (t); HRMS (ESI) *m/z* [M + H]$^{+}$ calcd. for $C_{13}H_{14}NO_4$ 248.0923, found 248.0918.

3-(4-Methoxy-3-oxo-1,3-dihydroisobenzofuran-1-yl)propanenitrile (4e)

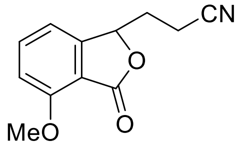

Colorless paste (104 mg, 48%); *Rf* 0.35 (1:2 hexanes/ethyl acetate); IR (ATR) 2247, 1754, 1600 cm^{-1}; ^{1}H NMR ($CDCl_3$, 500 MHz) δ 7.67 (t, 1H, J = 8.0 Hz), 7.00 (d, 1H, J = 8.0 Hz), 6.99 (d, 1H, J = 8.0 Hz), 5.49 (dd, 1H, J = 2.9, 8.7 Hz), 4.01 (s, 3H), 2.68-2.58 (m, 1H), 2.55-2.42 (m, 2H), 2.05-1.96 (m, 1H) ; ^{13}C{^{1}H} NMR ($CDCl_3$, 125 MHz) δ 167.6 (s), 158.7 (s), 150.6 (s), 136.7 (d), 118.4 (s), 113.2 (d), 113.1 (s), 111.3 (d), 77.3 (d), 56.0 (q), 30.7 (t), 13.3 (t); HRMS (ESI) *m/z* [M + H]$^{+}$ calcd. for $C_{12}H_{12}NO_3$ 218.0817, found 218.0812.

3-(4,5,6-Trimethoxy-3-oxo-1,3-dihydroisobenzofuran-1-yl)propanenitrile (4f)

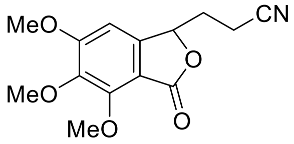

White solid (114 mg, 41%); *Rf* 0.45 (1:2 hexanes/ethyl acetate); mp 113−114 °C (recrystallized from 1:2 hexanes/ethyl acetate); IR (ATR) 2248, 1756, 1602 cm^{-1}; ^{1}H NMR ($CDCl_3$, 500 MHz) δ 6.63 (s, 1H), 5.40 (dd, 1H, J = 2.9, 8.9 Hz), 4.14 (s, 3H), 3.97 (s, 3H), 3.88 (s, 3H), 2.68-2.61 (m, 1H), 2.57-2.40 (m, 2H), 2.03-1.95 (m, 1H); ^{13}C{^{1}H} NMR ($CDCl_3$, 125 MHz) δ 167.2 (s), 160.0 (s), 152.6 (s), 145.7 (s), 142.2 (s), 118.5 (s), 110.2 (s), 99.1 (d), 77.1 (d), 62.3 (q), 61.4 (q), 56.5 (q), 31.0 (t), 13.4 (t); HRMS (ESI) *m/z* [M + H]$^{+}$ calcd. for $C_{14}H_{16}NO_5$ 278.1028, found 278.1018.

1-Hydroxy-4-methyl-2-naphthonitrile (3g)

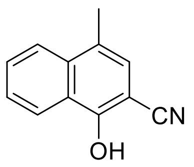

White solid (134 mg, 73%); *Rf* 0.6 (2:1 hexanes/ethyl acetate); mp 173−175 °C (recrystallized from 2:1 hexanes/ethyl acetate); IR (ATR) 3246 (br), 2227, 1623, 1575, 1509 cm^{-1}; ^{1}H NMR (CDCl$_3$, 500 MHz) δ 8.33 (d, 1H, J = 8.5 Hz), 7.90 (d, 1H, J = 8.5 Hz), 7.69-7.65 (m, 1H), 7.59-7.55 (m, 1H), 7.35 (brs, 1H), 7.15 (s, 1H), 2.56 (s, 3H); ^{13}C{^{1}H} NMR (CDCl$_3$, 125 MHz) δ 156.7 (s), 135.6 (s), 129.5 (d), 127.4 (s), 126.3 (d), 124.7 (d), 124.3 (d), 124.1 (s), 123.5 (d), 117.5 (s), 91.1 (s), 18.6 (q); HRMS (ESI) *m/z* [M + H]$^+$ calcd. for $C_{12}H_{10}NO$ 184.0762, found 184.0762.

3-(3-Oxo-1-phenyl-1,3-dihydroisobenzofuran-1-yl)propanenitrile (4h) [3]

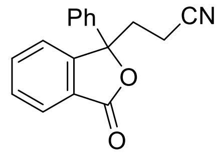

Colorless paste (63 mg, 24%); *Rf* 0.4 (2:1 hexanes/ethyl acetate); ^{1}H NMR (CDCl$_3$, 500 MHz) δ 8.04 (d, 1H, J =8.0 Hz), 7.86-7.81 (m, 1H), 7.73-7.60 (m, 4H), 7.55-7.45 (m, 3H), 3.06-2.99 (m, 1H), 2.68-2.60 (m, 1H), 2.50-2.39 (m, 2H); ^{13}C{^{1}H} NMR (CDCl$_3$, 125 MHz) δ 168.8 (s), 150.8 (s), 138.1 (s), 134.6 (d), 129.5 (d), 128.7 (d), 128.3 (d), 125.6 (d), 124.2 (d and s), 121.9 (d), 118.2 (s), 87.6 (s), 35.2 (t), 12.0 (t).

3-Phenylisobenzofuran-1(3*H*)-one (i) [4]

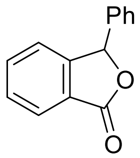

White solid (88 mg, 42%); *Rf* 0.45 (5:1 hexanes/ethyl acetate); mp 117−119 °C (recrystallized from 5:1 hexanes/ethyl acetate, lit.[4] 107−110 °C); ^{1}H NMR (CDCl$_3$, 500 MHz) δ 7.96 (d, 1H, J = 7.5 Hz), 7.65 (t, 1H, J = 7.5 Hz), 7.55 (t, 1H, J = 7.5 Hz), 7.40-7.36 (m, 3H), 7.34 (d, 1H, J = 7.5 Hz), 7.30-7.26 (m, 2H), 6.40 (s, 1H); ^{13}C{^{1}H} NMR (CDCl$_3$, 125 MHz) δ 170.5 (s), 149.7 (s), 136.4 (s), 134.3 (d), 129.33 (d), 129.28 (d), 129.0 (d), 126.9 (d), 125.62 (d), 125.58 (s), 122.8 (d), 82.7 (d).

3-(3-Oxobutyl)isobenzofuran-1(3*H*)-one (5a) [5]

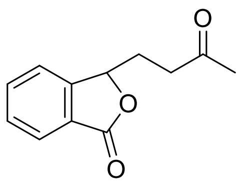

Colorless paste (174 mg, 85%); *Rf* 0.5 (1:1 hexanes/ethyl acetate); ^{1}H NMR (CDCl$_3$, 500 MHz) δ 7.90 (d, 1H, J = 7.5 Hz), 7.71-7.67 (m, 1H), 7.54 (t, 1H, J = 7.5 Hz), 7.49-7.47 (m, 1H), 5.52 (dd, 1H, J = 3.3, 8.8 Hz), 2.79-2.72 (m, 1H), 2.60-2.44 (m, 2H), 2.16 (s, 3H), 1.91-1.83 (m, 1H); ^{13}C{^{1}H} NMR (CDCl$_3$, 125 MHz) δ 207.1 (s), 170.1 (s), 149.3 (s), 134.0 (d), 129.1 (d), 125.6 (s), 125.4 (d), 121.7 (d), 80.0 (d), 38.0 (t), 29.8 (q), 28.2 (t).

6-Methoxy-3-(3-oxobutyl)isobenzofuran-1(3*H*)-one (5b)

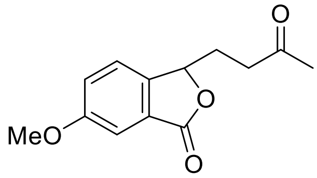

Colorless paste (180 mg, 77%); *Rf* 0.25 (2:1 hexanes/ethyl acetate); IR (ATR) 1760, 1714, 1674, 1625 cm^{-1}; ^{1}H NMR (CDCl$_3$, 500 MHz) δ 7.36 (d, 1H, J = 8.6 Hz), 7.32-7.30 (m, 1H), 7.26-7.23 (m, 1H), 5.47 (dd, 1H, J = 2.9, 8.5 Hz), 3.88 (s, 3H), 2.77-2.68 (m, 1H), 2.59-2.51 (m, 1H), 2.47-2.40 (m, 1H), 2.45 (s, 3H), 1.90-1.81 (m, 1H); ^{13}C{^{1}H} NMR (CDCl$_3$, 125 MHz) δ 206.9 (s), 169.9 (s), 160.3 (s), 141.5 (s), 126.9 (s), 122.51 (d), 122.48 (d), 107.1 (d), 78.7 (d), 55.4 (q), 37.8 (t), 29.6 (q), 28.1 (t); HRMS (ESI) *m/z* [M + Na]$^+$ calcd. for $C_{13}H_{14}O_4Na$ 257.0790, found 257.0788.

5,6-Dimethoxy-3-(3-oxobutyl)isobenzofuran-1(3*H*)-one (5c)

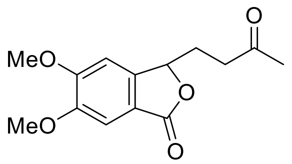

Colorless paste (233 mg, 88%); *Rf* 0.35 (1:1 hexanes/ethyl acetate); IR (ATR) 1747, 1714, 1602 cm^{-1}; ^{1}H NMR (CDCl$_3$, 500 MHz) δ 7.27 (s, 1H), 6.87 (s, 1H), 5.41 (dd, 1H, J = 2.9, 8.6 Hz), 3.99 (s, 3H), 3.94 (s, 3H), 2.79-2.70 (m, 1H), 2.58-2.42 (m, 2H), 2.16 (s, 3H), 1.87-1.79 (m, 1H); ^{13}C{^{1}H} NMR (CDCl$_3$, 125 MHz) δ 207.3 (s), 170.4 (s), 154.7 (s), 150.3 (s), 143.8 (s), 117.5 (s), 105.8 (d), 103.1 (d), 79.3 (d), 56.2 (q), 56.0 (q), 37.9 (t), 29.9 (q), 28.2 (t); HRMS (ESI) *m/z* [M + H]$^+$ calcd. for $C_{14}H_{17}O_5$ 265.1076, found 265.1075.

6,7-Dimethoxy-3-(3-oxobutyl)isobenzofuran-1(3*H*)-one (5d)

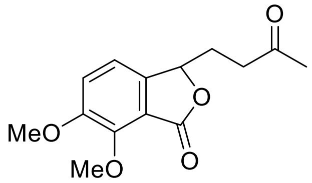

White solid (177 mg, 67%); *Rf* 0.35 (1:1 hexanes/ethyl acetate); mp 113−115 °C (recrystallized from 2:1 hexanes/ethyl acetate); IR (ATR) 1750, 1714, 1594, 1500 cm^{-1}; ^{1}H NMR (CDCl$_3$, 500 MHz) δ 7.23 (d, 1H, J = 8.0 Hz), 7.06 (d, 1H, J = 8.0 Hz), 5.38 (dd, 1H, J = 3.4, 8.6 Hz), 4.11 (s, 3H), 3.91 (s, 3H), 2.77-2.69 (m, 1H), 2.59-2.51 (m, 1H), 2.45-2.37 (m, 1H), 2.16 (s, 3H), 1.87-1.78 (m, 1H); ^{13}C{^{1}H} NMR (CDCl$_3$, 125 MHz) δ 206.9 (s), 167.4 (s), 152.2 (s), 147.7 (s), 142.0 (s), 119.2 (d), 117.7 (s), 116.1 (d), 78.5 (d), 61.8 (q), 56.4 (q), 37.8 (t), 29.6 (q), 28.4 (t); HRMS (ESI) *m/z* [M + H]$^+$ calcd. for $C_{14}H_{17}O_5$ 265.1076, found 265.1076.

7-Methoxy-3-(3-oxobutyl)isobenzofuran-1(3*H*)-one (5e)

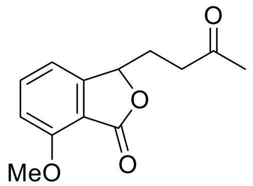

Colorless paste (155 mg, 66%); *Rf* 0.4 (1:2 hexanes/ethyl acetate); IR (ATR) 1757, 1711, 1605 cm^{-1}; ^{1}H NMR (CDCl$_3$, 500 MHz) δ 7.62 (t, 1H, J = 8.0 Hz), 7.00 (d, 1H, J = 8.0 Hz), 6.94 (d, 1H, J = 8.0 Hz), 5.42 (dd, 1H, J = 3.4, 8.6 Hz), 4.00 (s, 3H), 2.79-2.70 (m, 1H), 2.59-2.50 (m, 1H), 2.48-2.39 (m, 1H), 2.15 (s, 3H), 1.89-1.79 (m, 1H); ^{13}C{^{1}H} NMR (CDCl$_3$, 125 MHz) δ 207.0 (s), 168.0 (s), 158.2 (s), 152.0 (s), 136.2 (d), 113.2 (d), 113.0 (s), 110.6 (d), 78.7 (d), 55.7 (q), 37.9 (t), 29.7 (q), 28.1 (t); HRMS (ESI) *m/z* [M + H]$^+$ calcd. for $C_{13}H_{15}O_4$ 235.0970, found 235.0967.

5,6,7-Trimethoxy-3-(3-oxobutyl)isobenzofuran-1(3*H*)-one (5f)

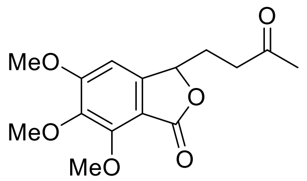

White solid (215 mg, 73%); *Rf* 0.3 (1:1 hexanes/ethyl acetate); mp 118−120 °C (recrystallized from 1:1 hexanes/ethyl acetate); IR (ATR) 1745, 1715, 1598 cm^{-1}; ^{1}H NMR (CDCl$_3$, 500 MHz) δ 6.63 (s, 1H), 5.32 (dd, 1H, J = 3.0, 9.0 Hz), 4.14 (s, 3H), 3.95 (s, 3H), 3.87 (s, 3H), 2.80-2.72 (m, 1H), 2.61-2.53 (m, 1H), 2.47-2.39 (m, 1H), 2.17 (s, 3H), 1.83-1.75 (m, 1H); ^{13}C{^{1}H} NMR (CDCl$_3$, 125 MHz) δ 207.1 (s), 167.5 (s), 159.5 (s), 151.9 (s), 147.1 (s), 141.5 (s), 110.0 (s), 99.2 (d), 78.4 (d), 61.9 (q), 61.0 (q), 56.2 (q), 37.9 (t), 29.7 (q), 28.3 (t); HRMS (ESI) *m/z* [M + H]$^+$ calcd. for $C_{15}H_{19}O_6$ 295.1182, found 295.1180.

3-Methyl-3-(3-oxobutyl)isobenzofuran-1(3*H*)-one (5g)

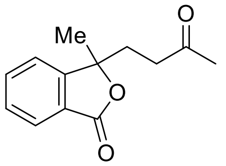

Colorless paste (162 mg, 74%); *Rf* 0.25 (2:1 hexanes/ethyl acetate); IR (ATR) 1764, 1714, 1615, 1600 cm^{-1}; ^{1}H NMR ($CDCl_3$, 500 MHz) δ 7.88 (d, 1H, *J* = 7.5 Hz), 7.74-7.70 (m, 1H), 7.58-7.54 (m, 1H), 7.44 (d, 1H, *J* = 8.0 Hz), 2.52-2.44 (m, 1H), 2.41-2.34 (m, 1H), 2.26-2.19 (m, 1H), 2.14-2.07 (m, 1H), 2.04 (s, 3H), 1.67 (s, 3H); ^{13}C{^{1}H} NMR ($CDCl_3$, 125 MHz) δ 206.4 (s), 169.1 (s), 152.6 (s), 133.9 (d), 128.7 (d), 125.2 (s), 125.1 (d), 120.8 (d), 86.2 (s), 37.0 (t), 32.6 (t), 29.3 (q), 25.5 (q); HRMS (ESI) *m/z* [M + H]$^+$ calcd. for $C_{13}H_{15}O_3$ 219.1021, found 219.1018.

3-(3-Oxobutyl)-3-phenylisobenzofuran-1(3*H*)-one (5h)

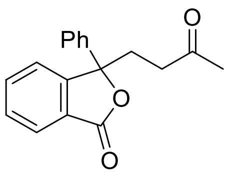

Colorless paste (207 mg, 74%); *Rf* 0.55 (1:1 hexanes/ethyl acetate); IR (ATR) 1772, 1712, 1611, 1600 cm^{-1}; ^{1}H NMR ($CDCl_3$, 500 MHz) δ 7.89 (d, 1H, *J* = 7.5 Hz), 7.88-7.63 (m, 1H), 7.55-7.49 (m, 4H), 7.39-7.35 (m, 2H), 7.33-7.29 (m, 1H), 2.85-2.77 (m, 1H), 2.49-2.35 (m, 3H), 2.02 (s, 3H); ^{13}C{^{1}H} NMR ($CDCl_3$, 125 MHz) δ 207.0 (s), 169.7 (s), 152.7 (s), 139.7 (s), 134.5 (d), 129.3 (d), 128.9 (d), 128.3 (d), 125.9 (d), 125.0 (s), 124.7 (d), 122.1 (d), 89.1 (s), 37.9 (t), 33.6 (t), 30.0 (q); HRMS (ESI) *m/z* [M + H]$^+$ calcd. for $C_{18}H_{17}O_3$ 281.1178, found 281.1178.

Methyl 1-hydroxy-2-naphthoate (8) [1]

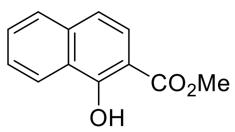

White solid (8 mg, 4%); *Rf* 0.55 (10:1 hexanes/ethyl acetate); mp 83−85 °C (recrystallized from 10:1 hexanes/ethyl acetate, lit.[1] 84−86 °C); ^{1}H NMR ($CDCl_3$, 500 MHz) δ 11.98 (brs, 1H), 8.41 (d, 1H, *J* = 8.6 Hz), 7.76 (d, 2H, *J* = 8.6 Hz), 7.62-7.58 (m, 1H), 7.54-7.50 (m, 1H), 7.28 (d, 1H, *J* = 8.6 Hz), 3.99 (s, 3H); ^{13}C{^{1}H} NMR ($CDCl_3$, 125 MHz) δ 171.4 (s), 160.9 (s), 137.2 (s), 129.4 (d), 127.4 (d), 125.7 (d), 124.8 (s), 124.2 (d), 123.9 (d), 118.6 (d), 105.6 (s), 52.3 (q).

[1,1'-biisobenzofuran]-3,3'(1*H*,1'*H*)-dione (9) {65:35 diastereomeric mixture, (*R,R*)-9 has been reported [6]}

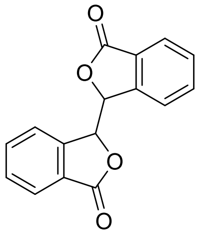

White solid (83 mg, 62 %); IR (ATR) 1763, 1725, 1598 cm^{-1}; ^{1}H NMR ($CDCl_3$/DMSO-d_6, 500 MHz) δ 7.98 (t, 0.65H, *J* = 7.5 Hz), 7.81 (d, 0.35H, *J* = 7.6 Hz), 7.75-7.70 (m, 0.65H), 7.69-7.62 (m, 1H), 7.59 (d, 0.35H, *J* = 7.5 Hz), 7.52-7.48 (m, 0.35H), 7.37 (d, 0.65H, *J* = 7.7 Hz), 6.12 (s, 0.35H), 5.76 (s, 0.65H); ^{13}C{^{1}H} NMR ($CDCl_3$/DMSO-d_6, 125 MHz) δ 168.3 (s), 168.1 (s), 144.5 (s), 143.7 (s), 133.5 (d), 133.4 (d), 129.4 (d), 129.0 (d), 125.9 (s), 125.5 (s), 124.9 (d), 124.7 (d), 122.1 (d), 121.8 (d), 79.1 (d), 77.8 (d); HRMS (ESI) *m/z* [M + H]$^+$ calcd. for $C_{16}H_{10}O_4Na$ 289.0477, found 289.0471.

^{1}H and $^{13}C\{^{1}H\}$ NMR spectra of compounds

^{1}H NMR Spectrum of 1a (500 MHz, $CDCl_3$)

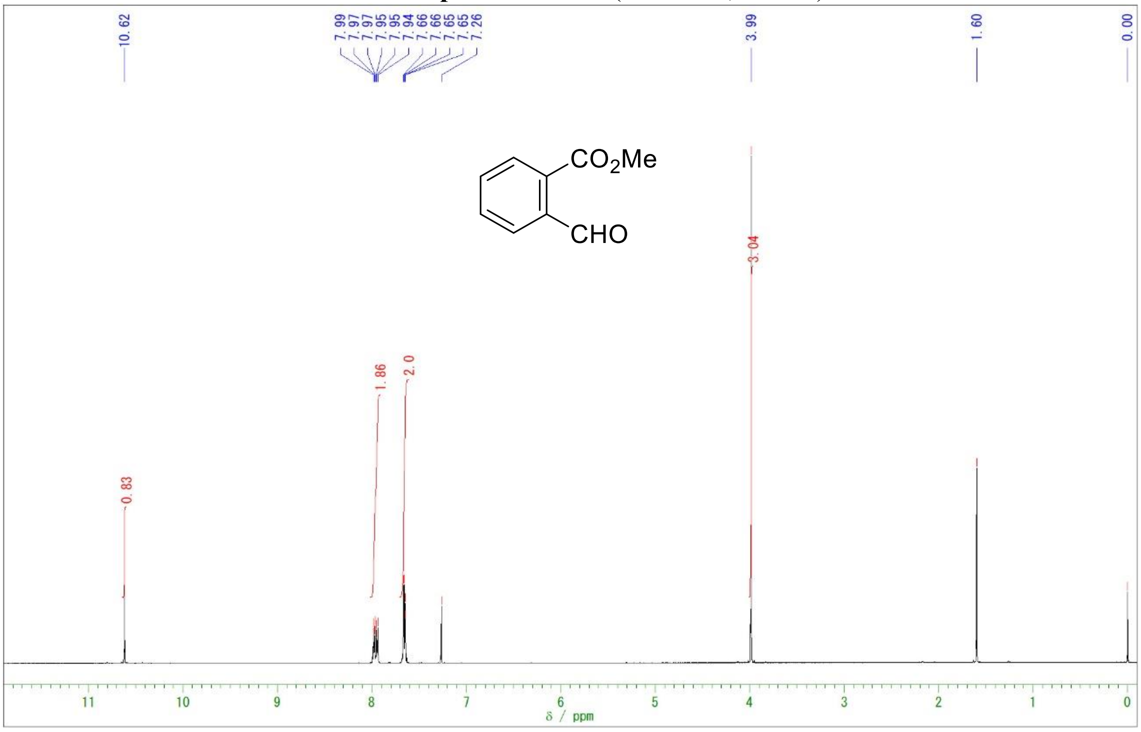

$^{13}C\{^{1}H\}$ NMR Spectrum of 1a (125 MHz, $CDCl_3$)

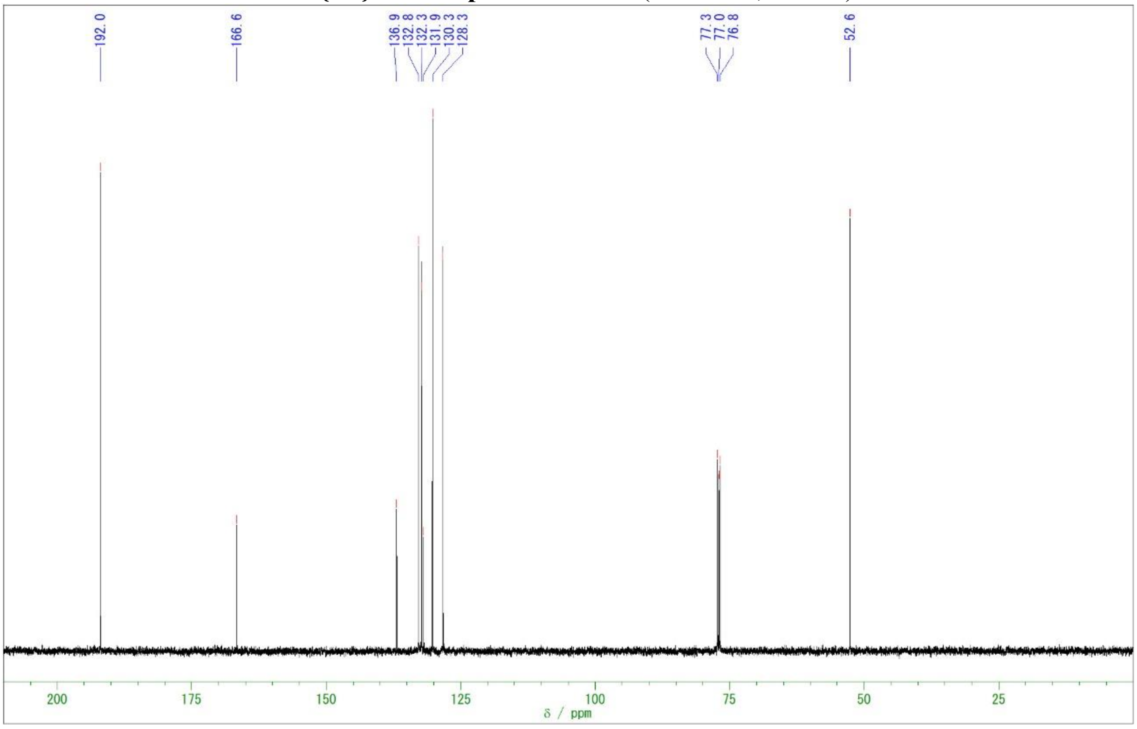

^{1}H NMR Spectrum of 1b (500 MHz, $CDCl_3$)

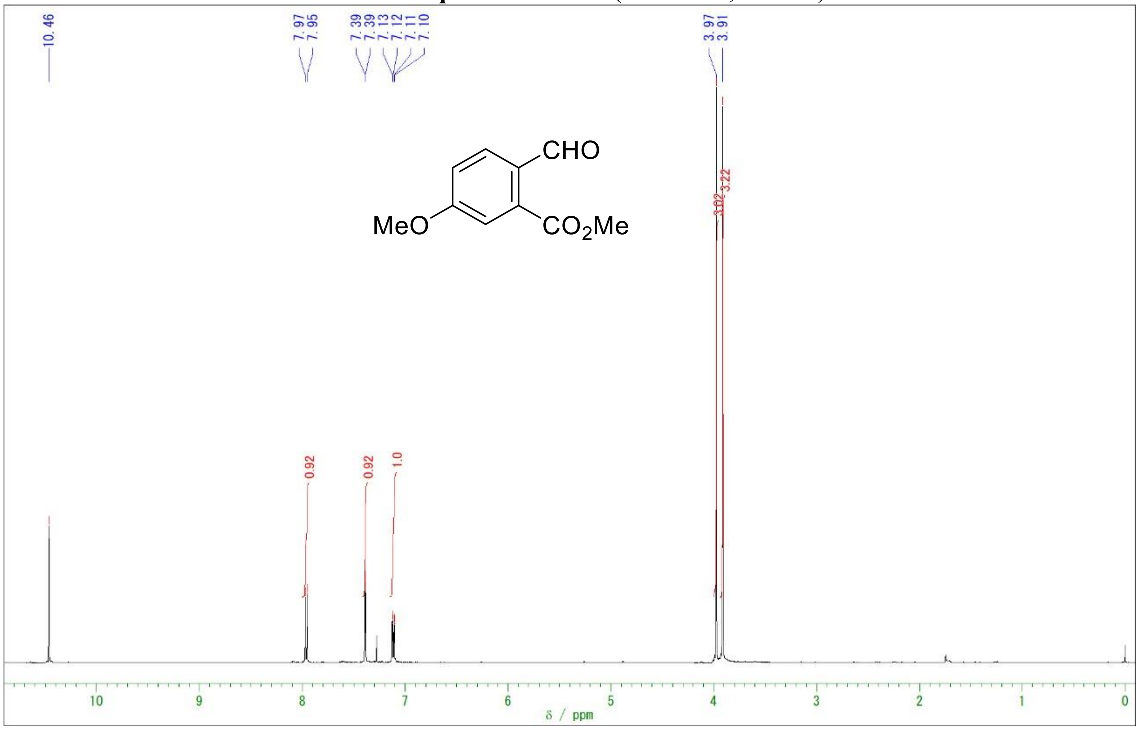

^{13}C{^{1}H} NMR Spectrum of 1b (125 MHz, $CDCl_3$)

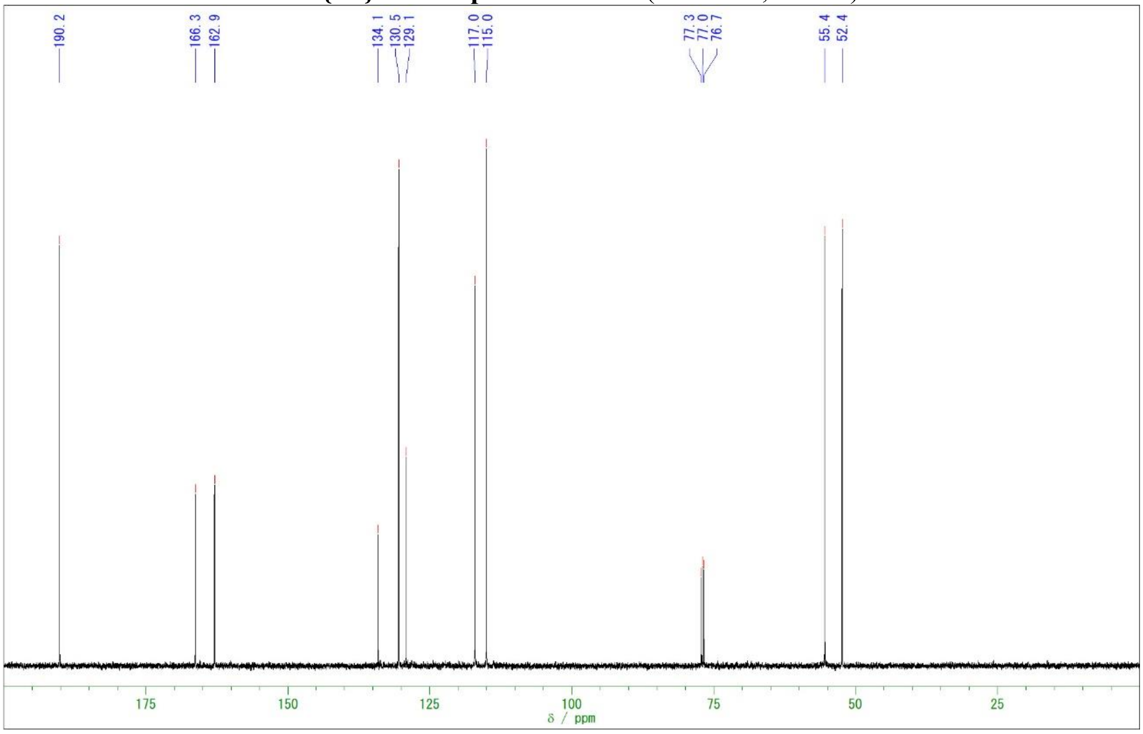

^{1}H NMR Spectrum of 1c (500 MHz, $CDCl_3$)

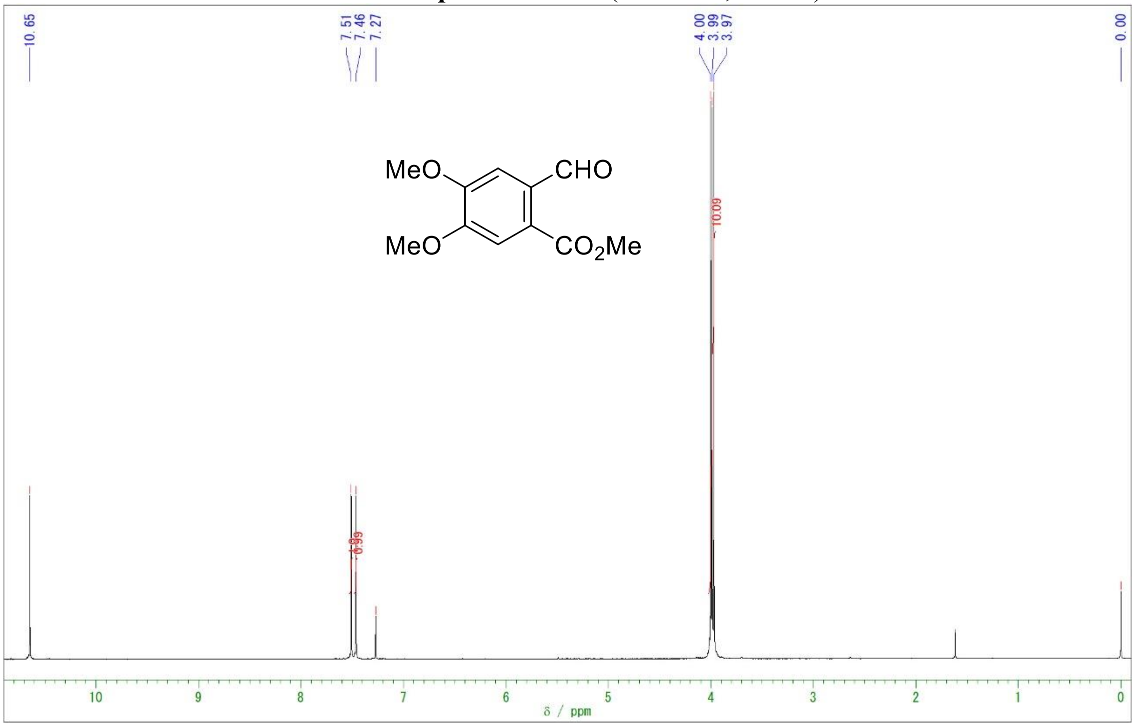

^{13}C{^{1}H} NMR Spectrum of 1c (125 MHz, $CDCl_3$)

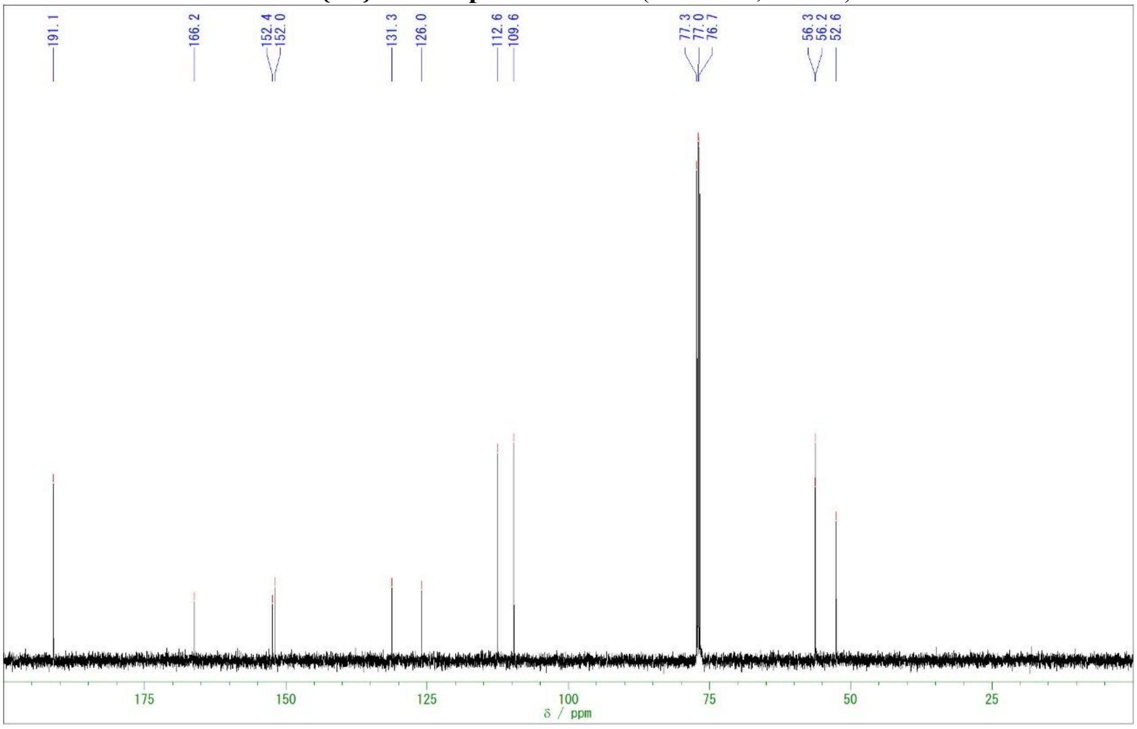

^{1}H NMR Spectrum of 1d (500 MHz, $CDCl_3$)

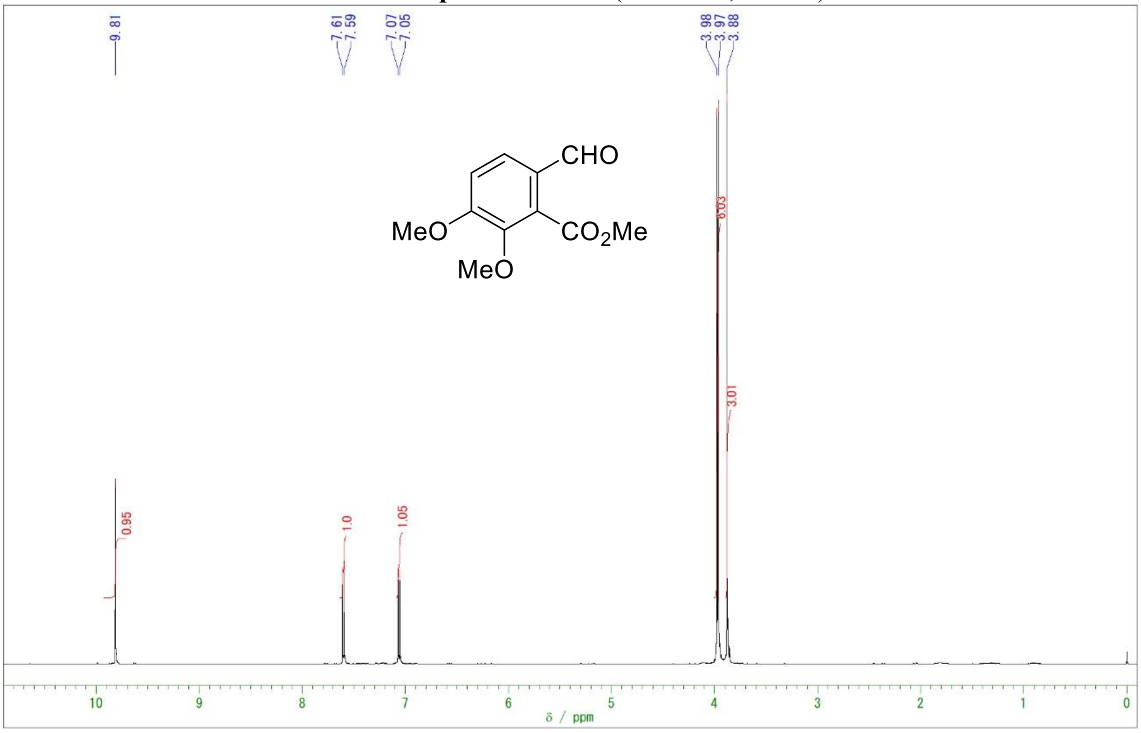

^{13}C{^{1}H} NMR Spectrum of 1d (125 MHz, $CDCl_3$)

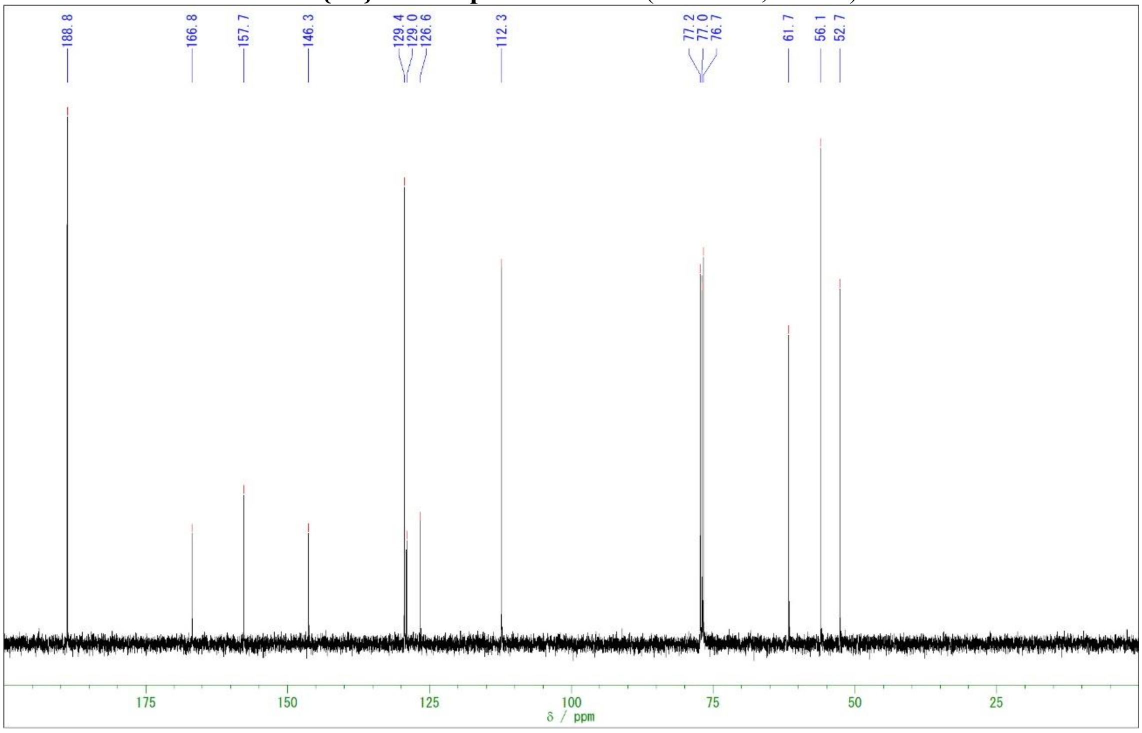

^{1}H NMR Spectrum of 1e (500 MHz, $CDCl_3$)

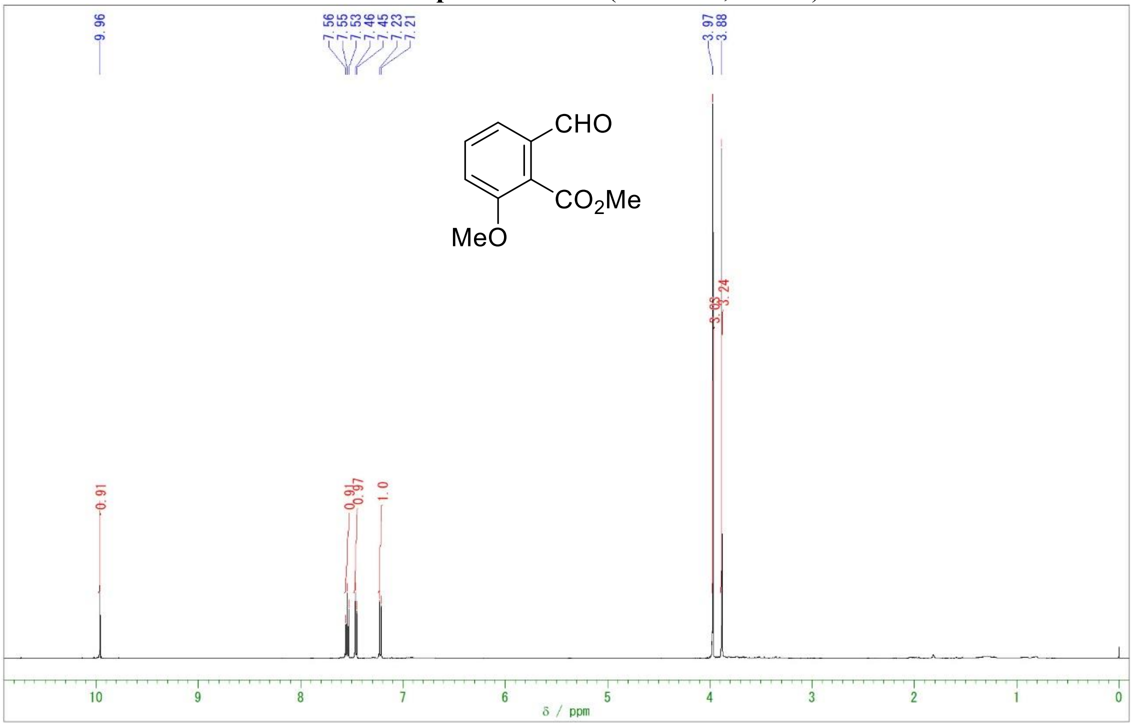

^{13}C{^{1}H} NMR Spectrum of 1e (125 MHz, $CDCl_3$)

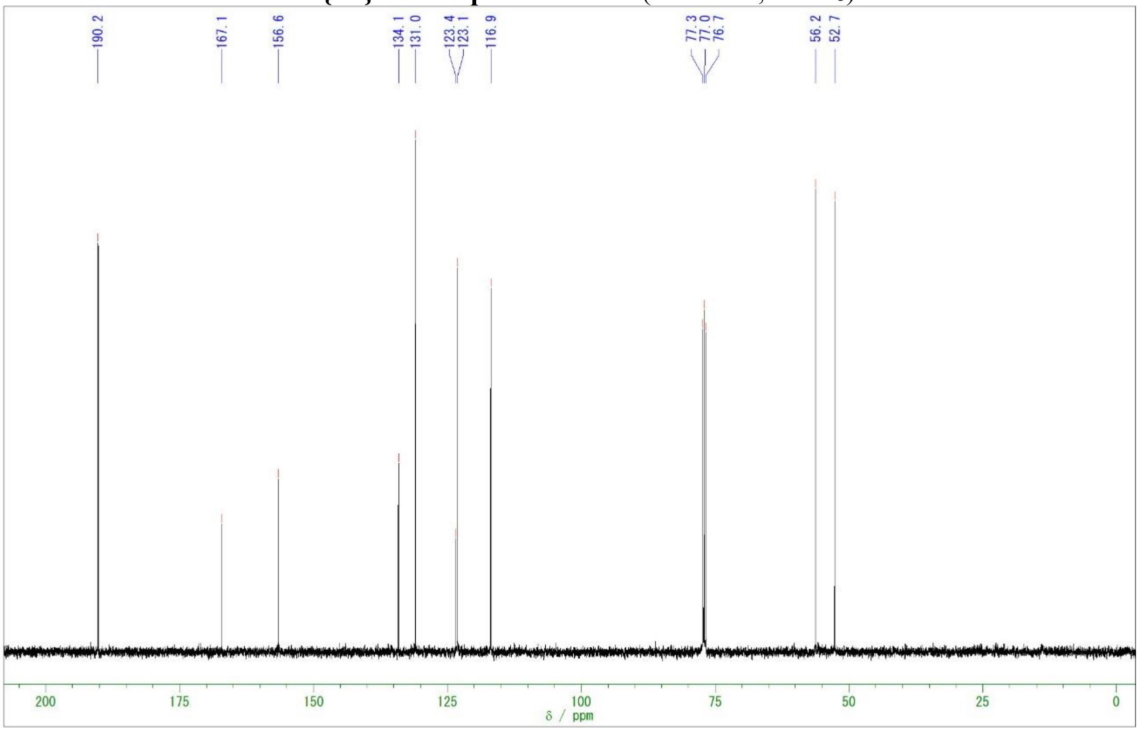

^{1}H NMR Spectrum of 1f (500 MHz, $CDCl_3$)

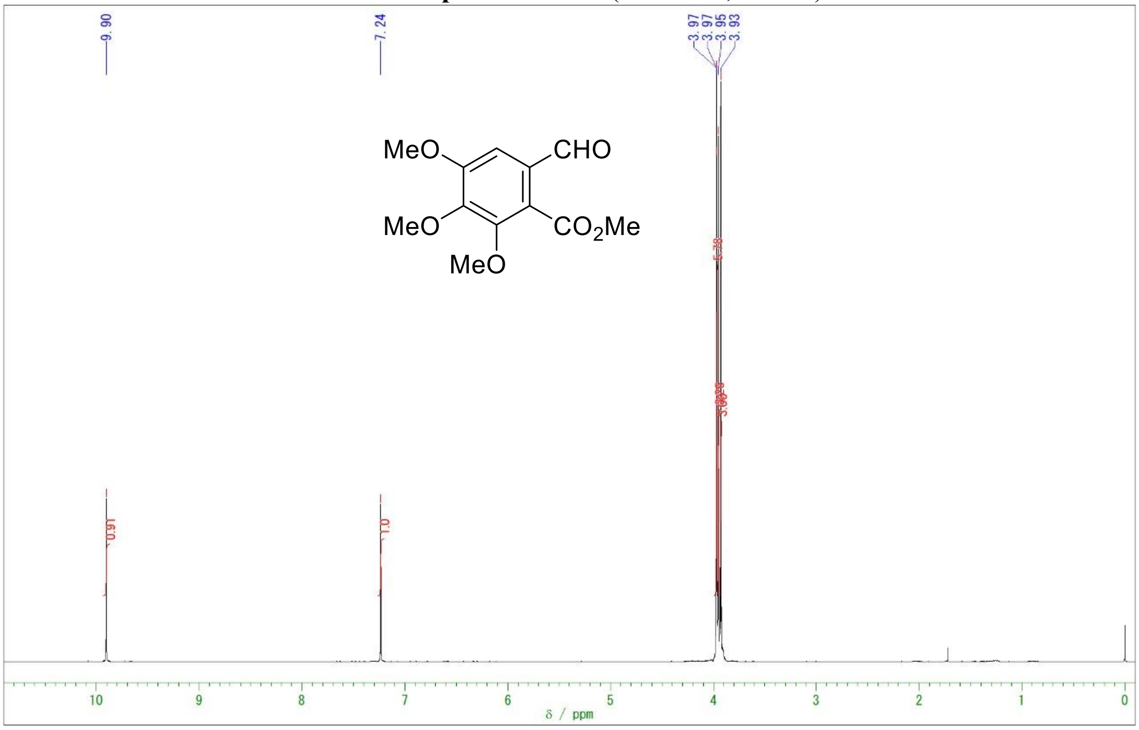

^{13}C{^{1}H} NMR Spectrum of 1f (125 MHz, $CDCl_3$)

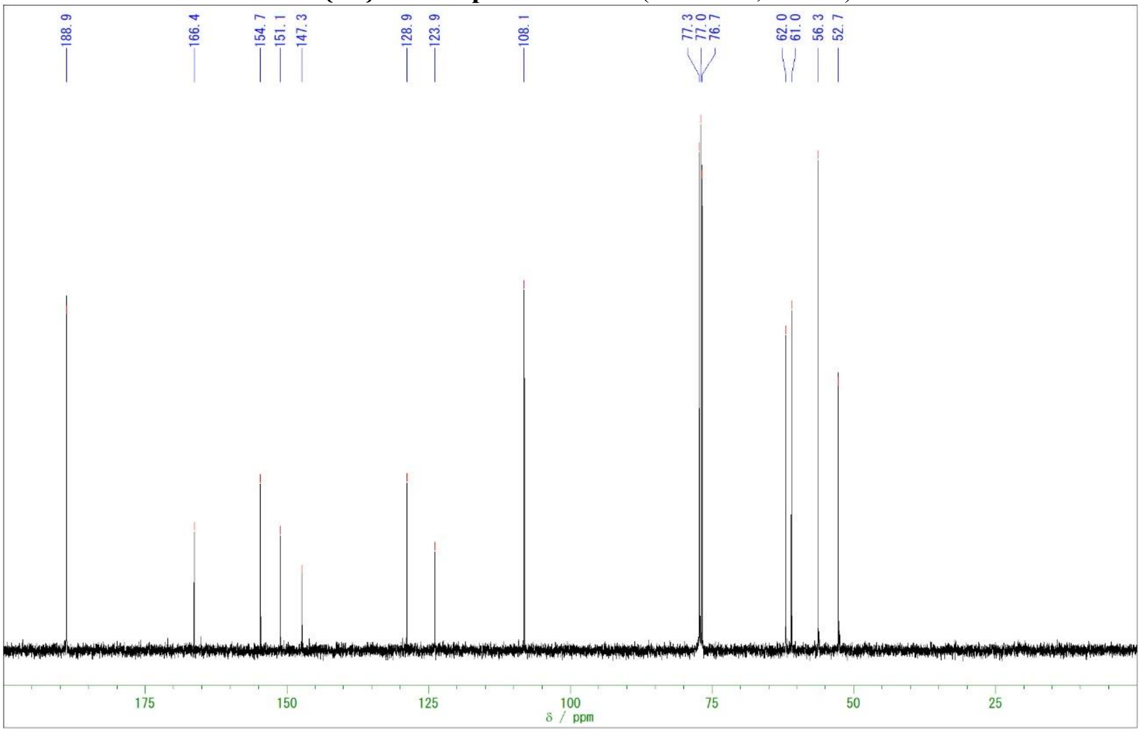

^{1}H NMR Spectrum of 1g (500 MHz, $CDCl_3$))

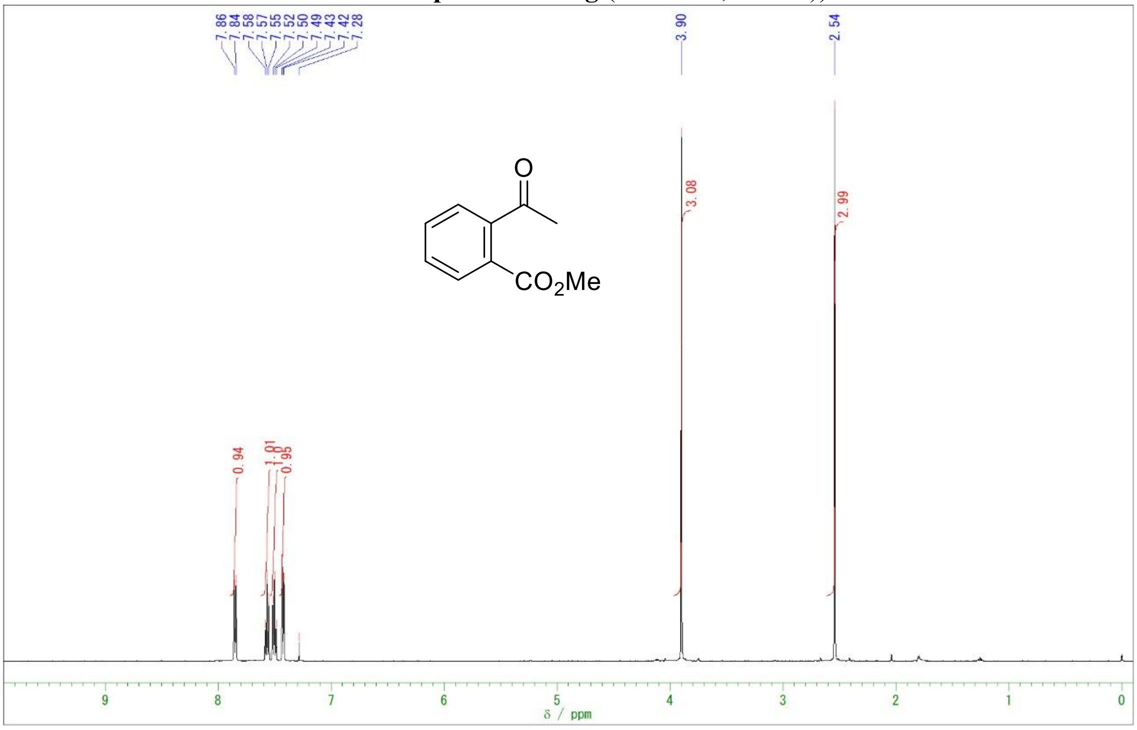

^{13}C{^{1}H} NMR Spectrum of 1g (125 MHz, $CDCl_3$))

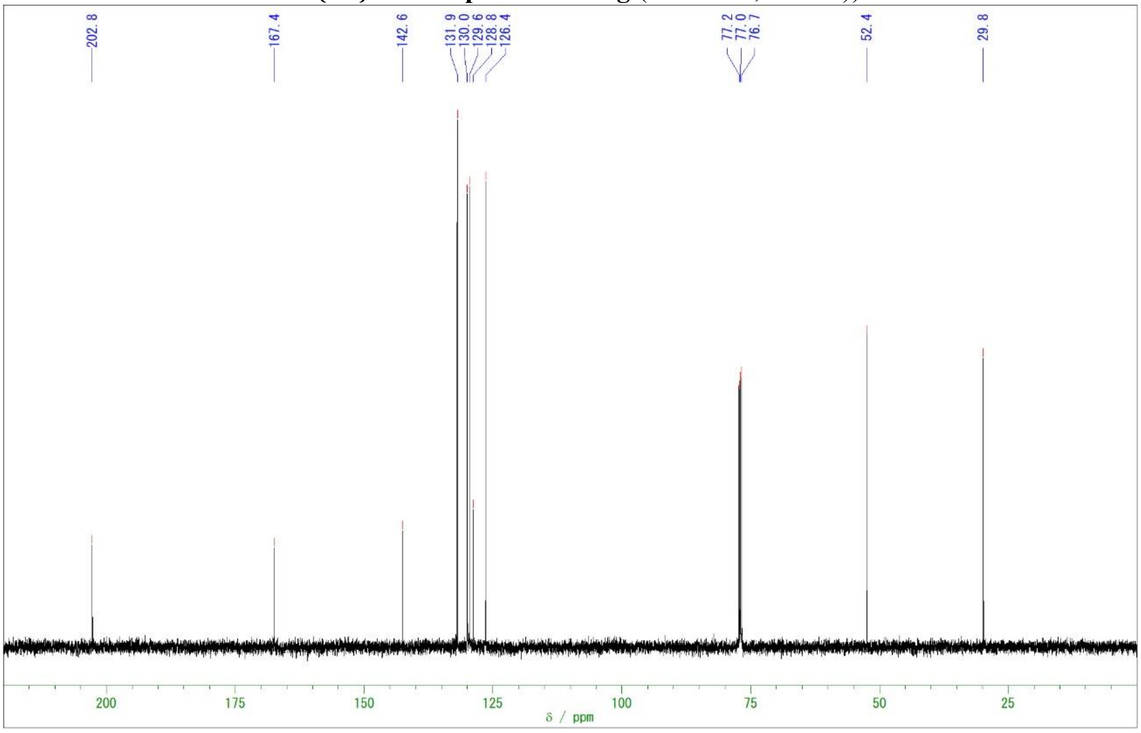

^{1}H NMR Spectrum of 1h (500 MHz, $CDCl_3$)

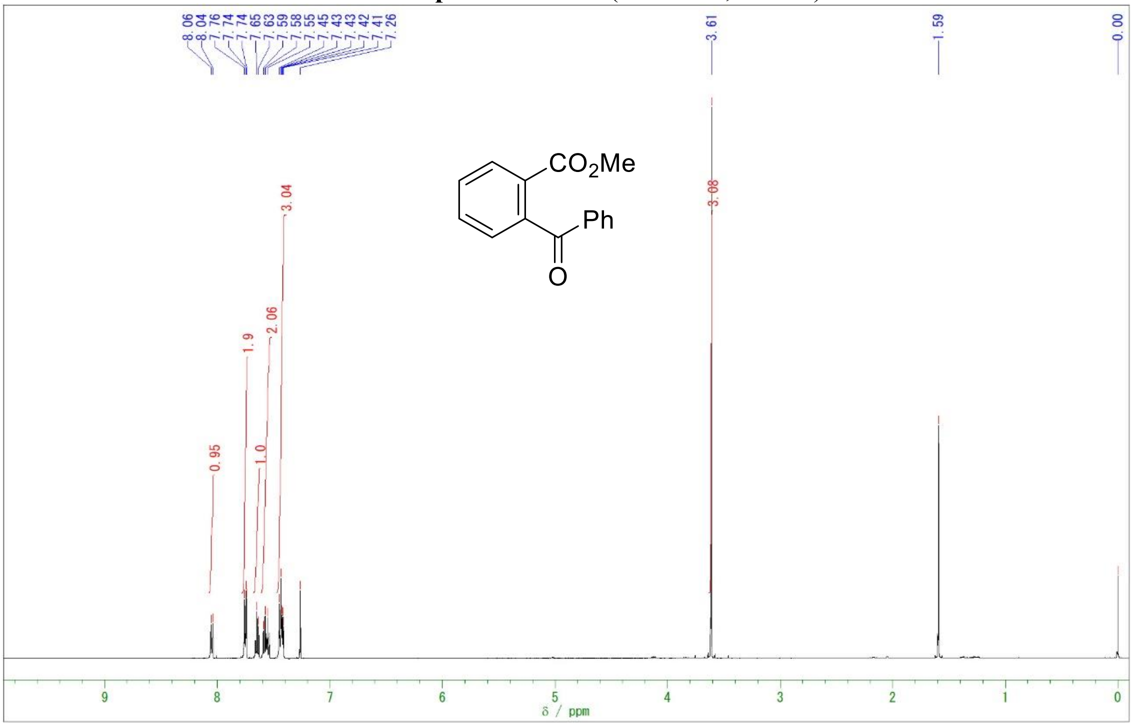

^{13}C{^{1}H} NMR Spectrum of 1h (125 MHz, $CDCl_3$)

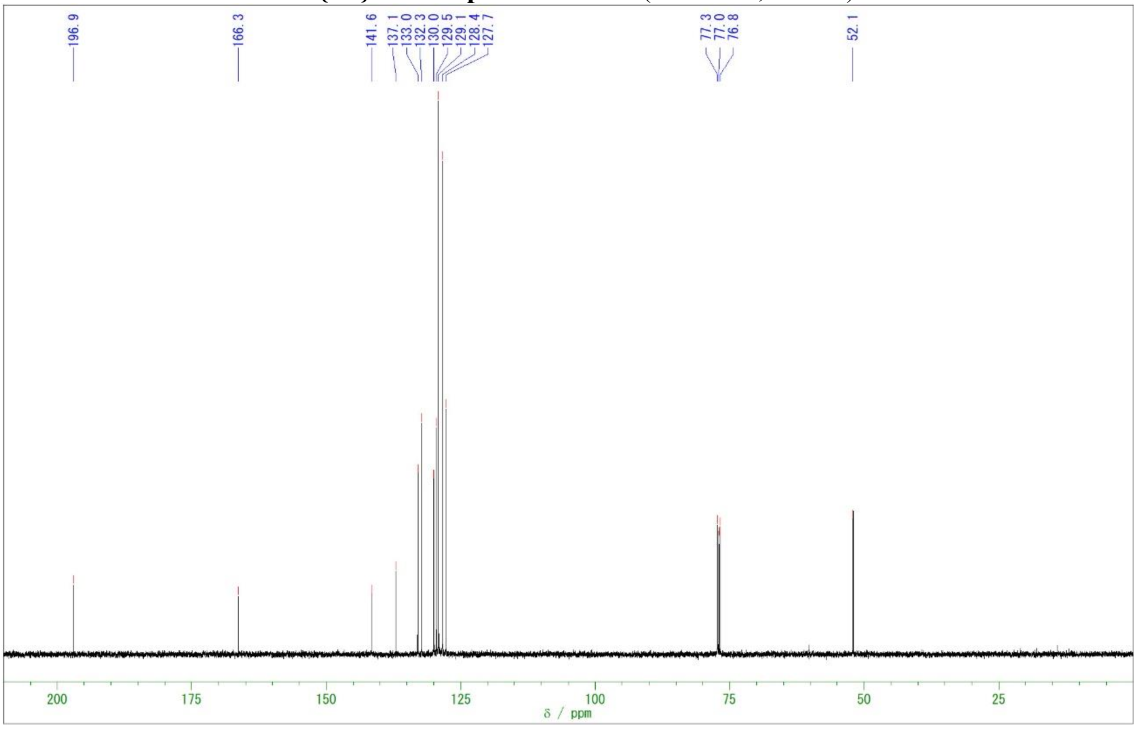

^{1}H NMR Spectrum of 6a (impure) (500 MHz, $CDCl_3$)

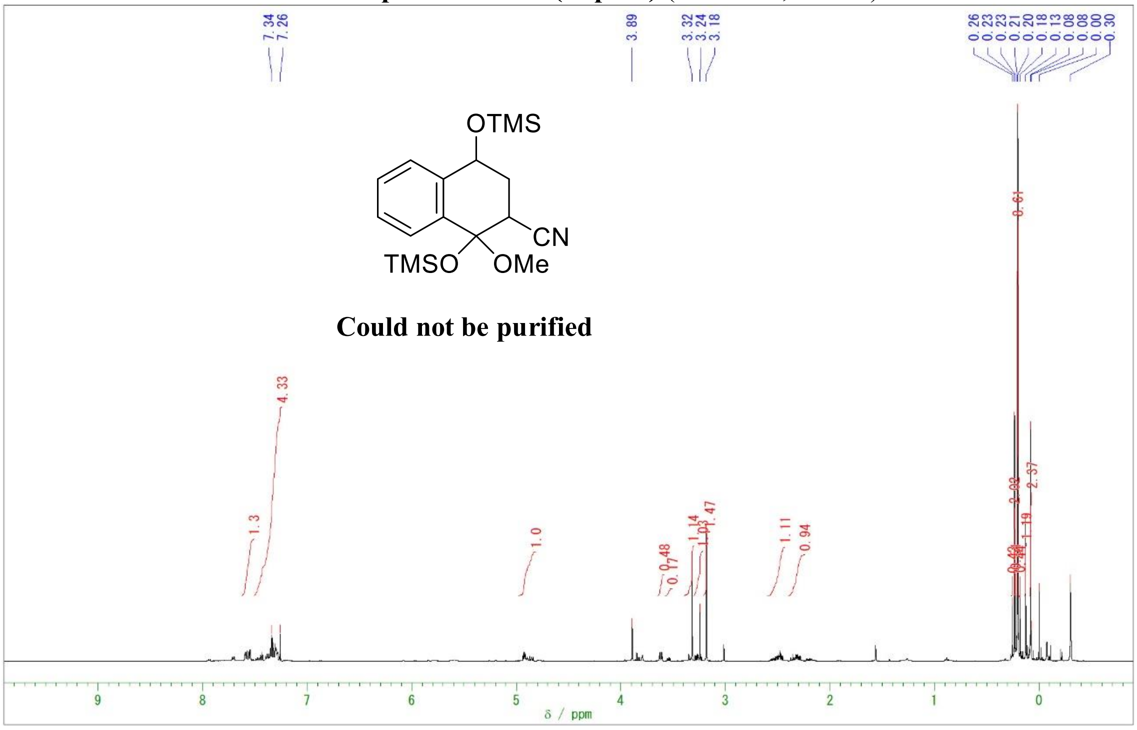

^{13}C{^{1}H} NMR Spectrum of 6a (impure) (125 MHz, $CDCl_3$)

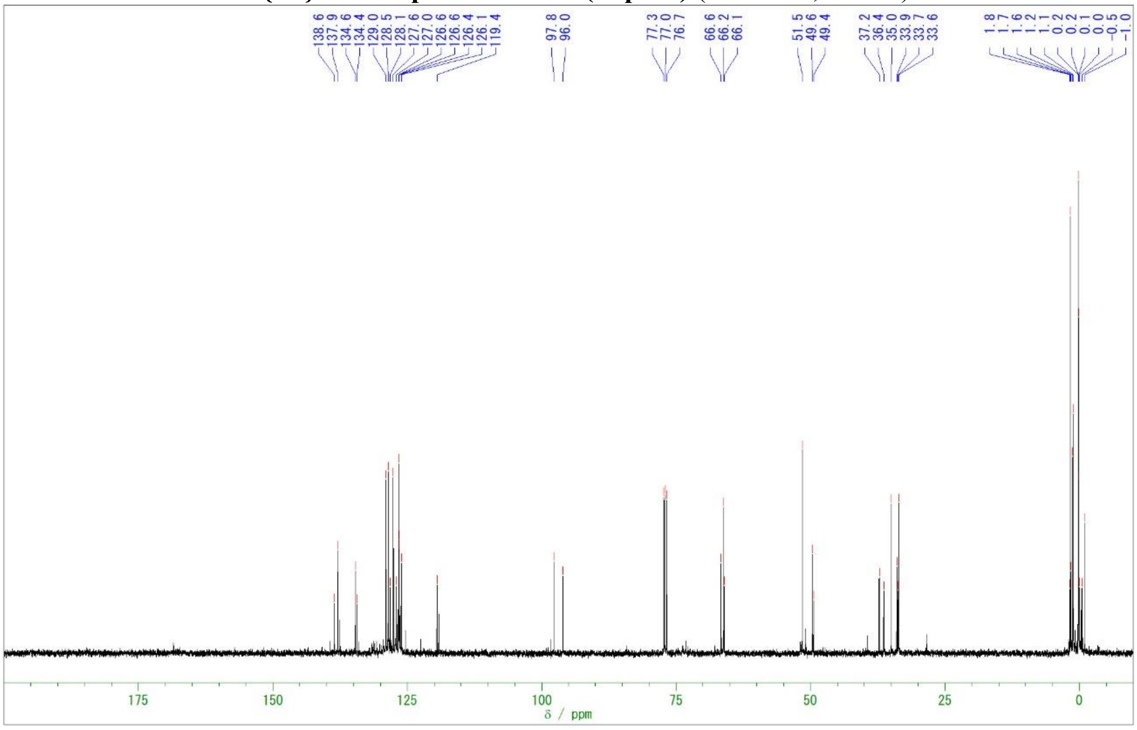

^{1}H NMR Spectrum of 7a (72:28 dr) (500 MHz, $CDCl_3$)

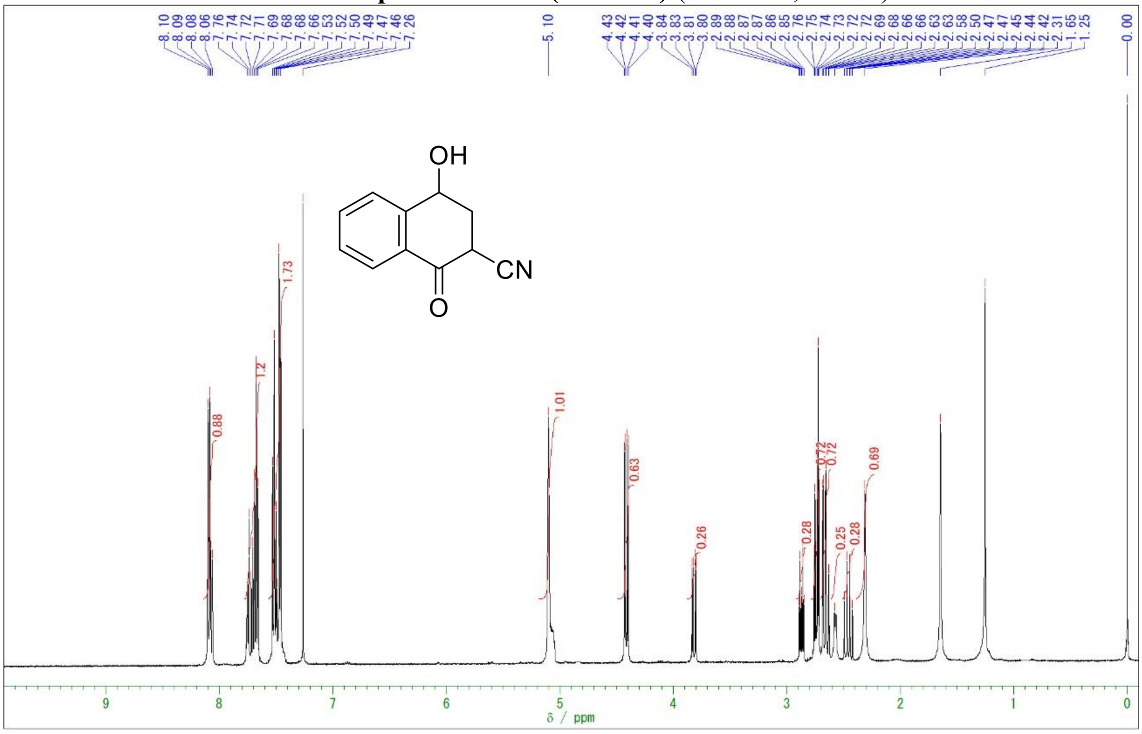

^{13}C{^{1}H} NMR Spectrum of 7a (125 MHz, $CDCl_3$/DMSO-*d6*)

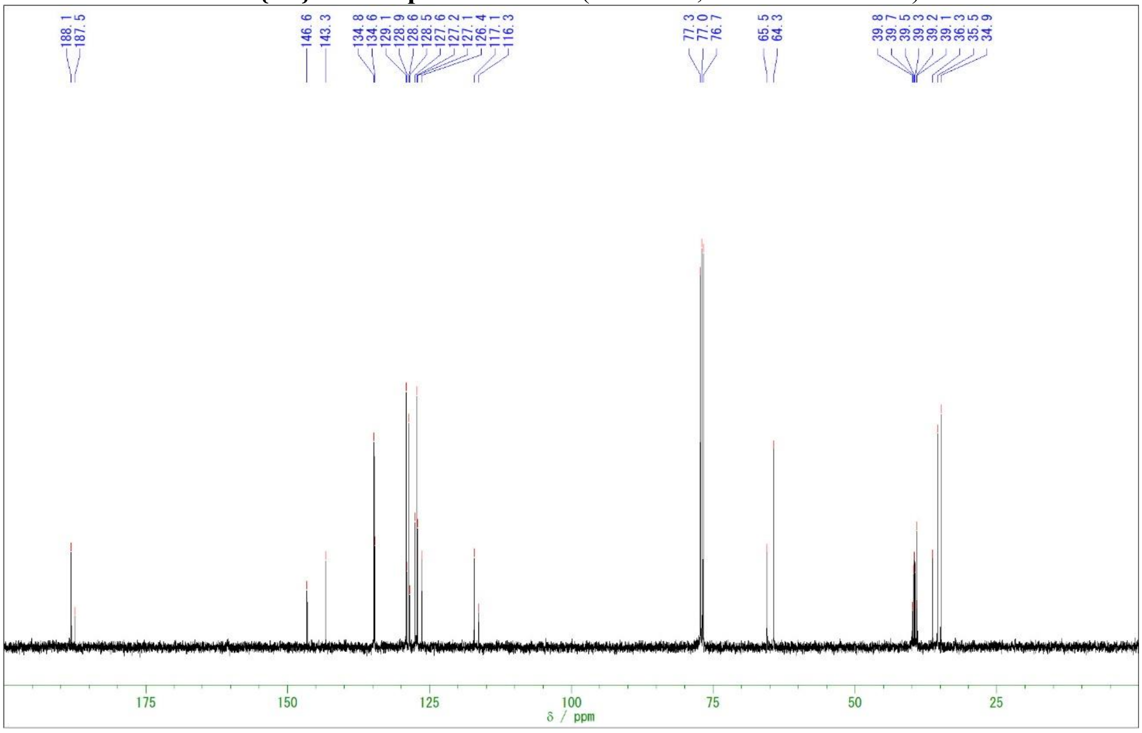

^{1}H NMR Spectrum of 3a (500 MHz, $CDCl_3$)

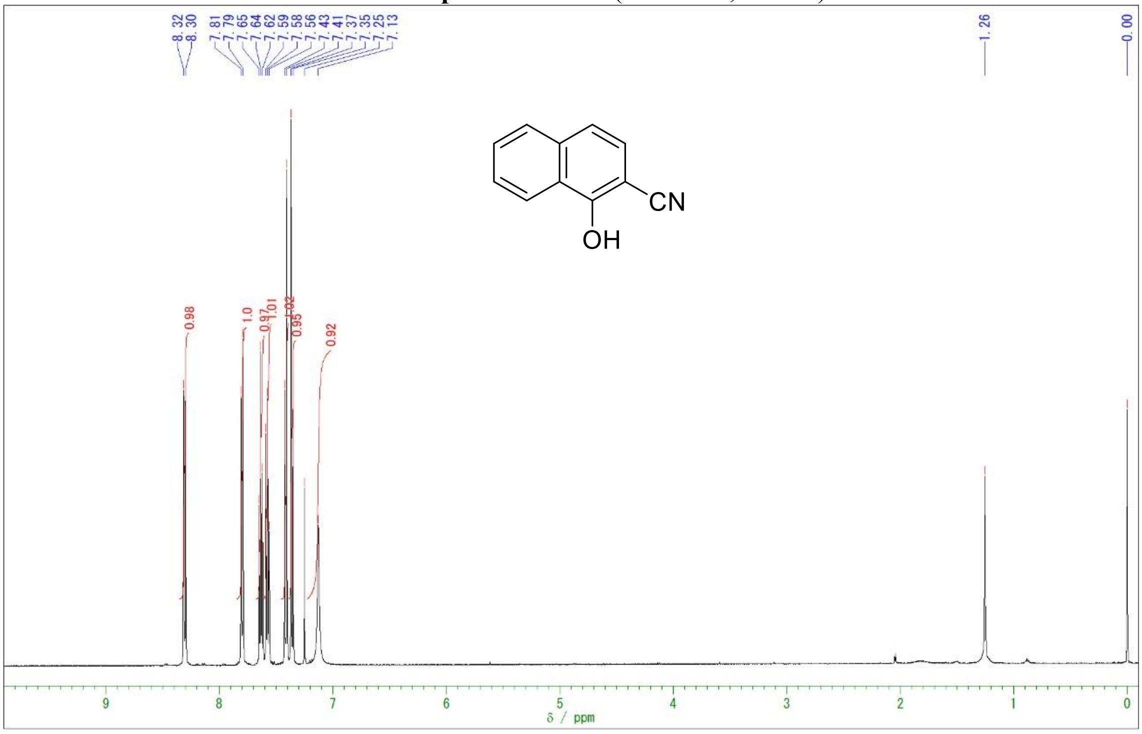

^{13}C{^{1}H} NMR Spectrum of 3a (125 MHz, $CDCl_3$)

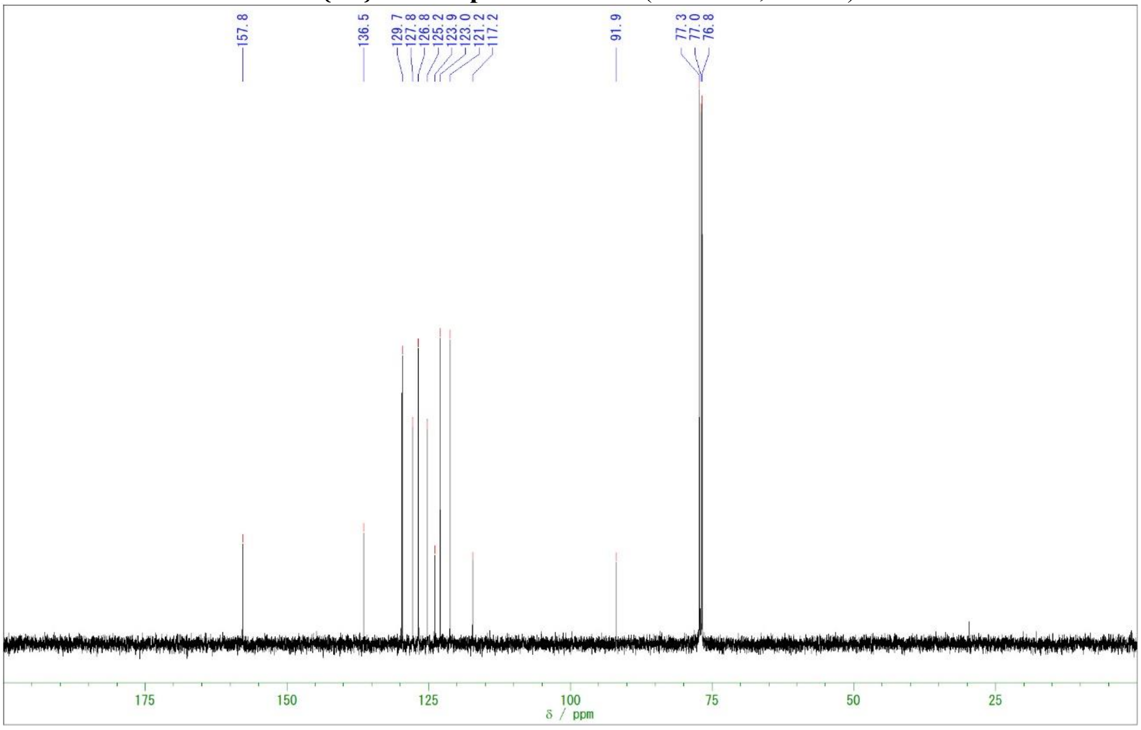

^{1}H NMR Spectrum of 3b (500 MHz, $CDCl_3$)

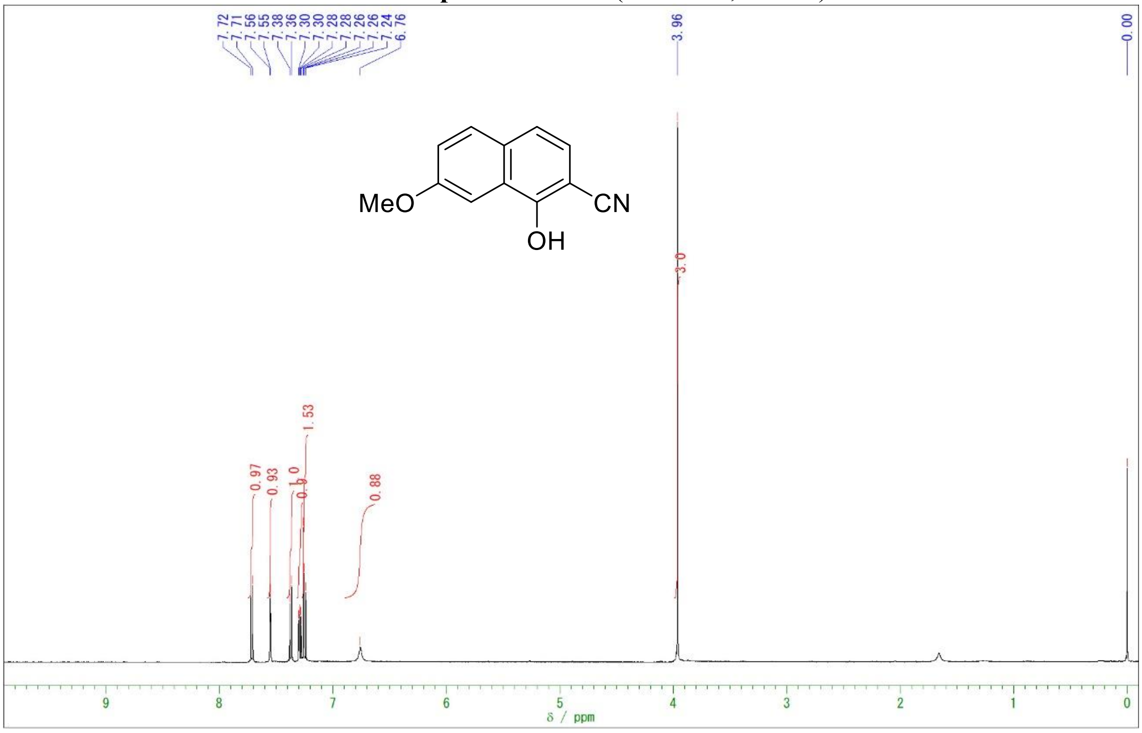

$^{13}C\{^{1}H\}$ NMR Spectrum of 3b (125 MHz, $CDCl_3$)

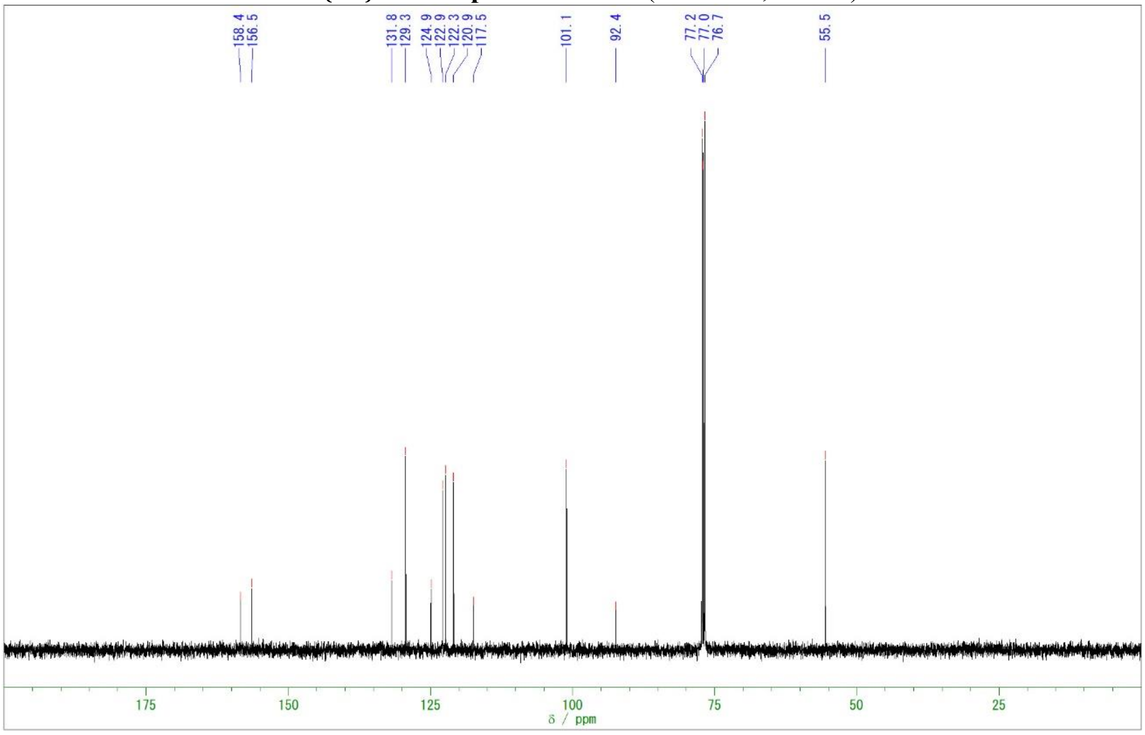

^{1}H NMR Spectrum of 3c (500 MHz, $CDCl_3$)

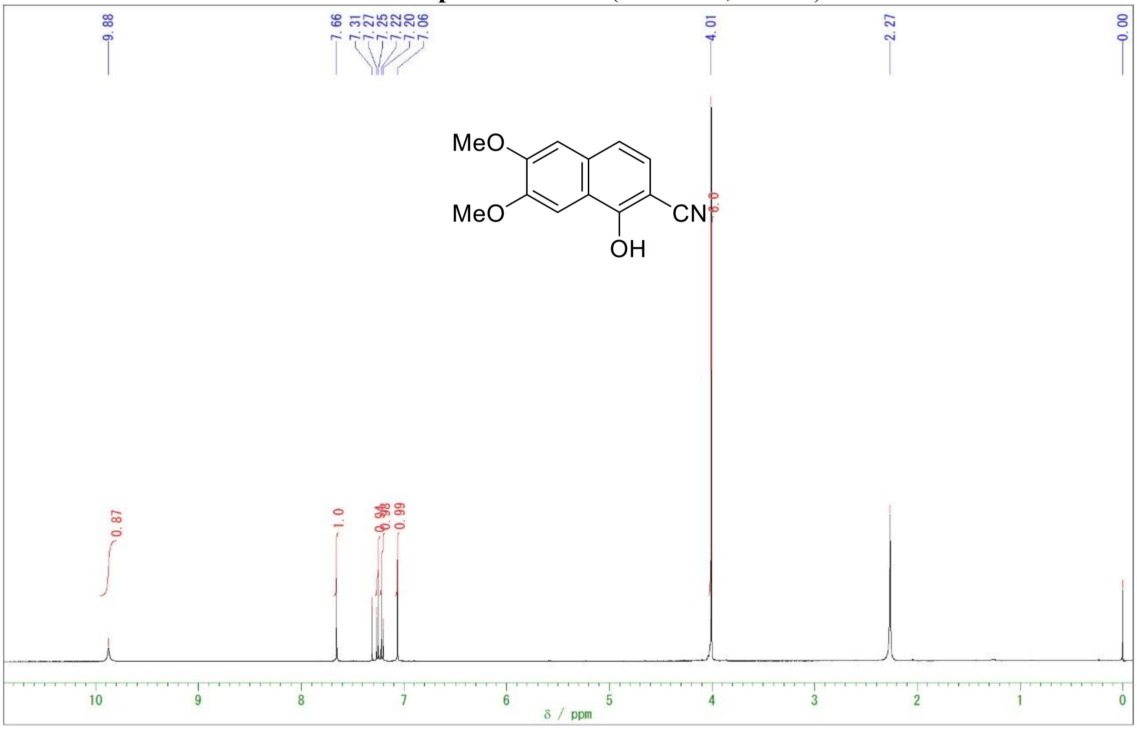

^{13}C{^{1}H} NMR Spectrum of 3c (125 MHz, $CDCl_3$/DMSO-*d6*)

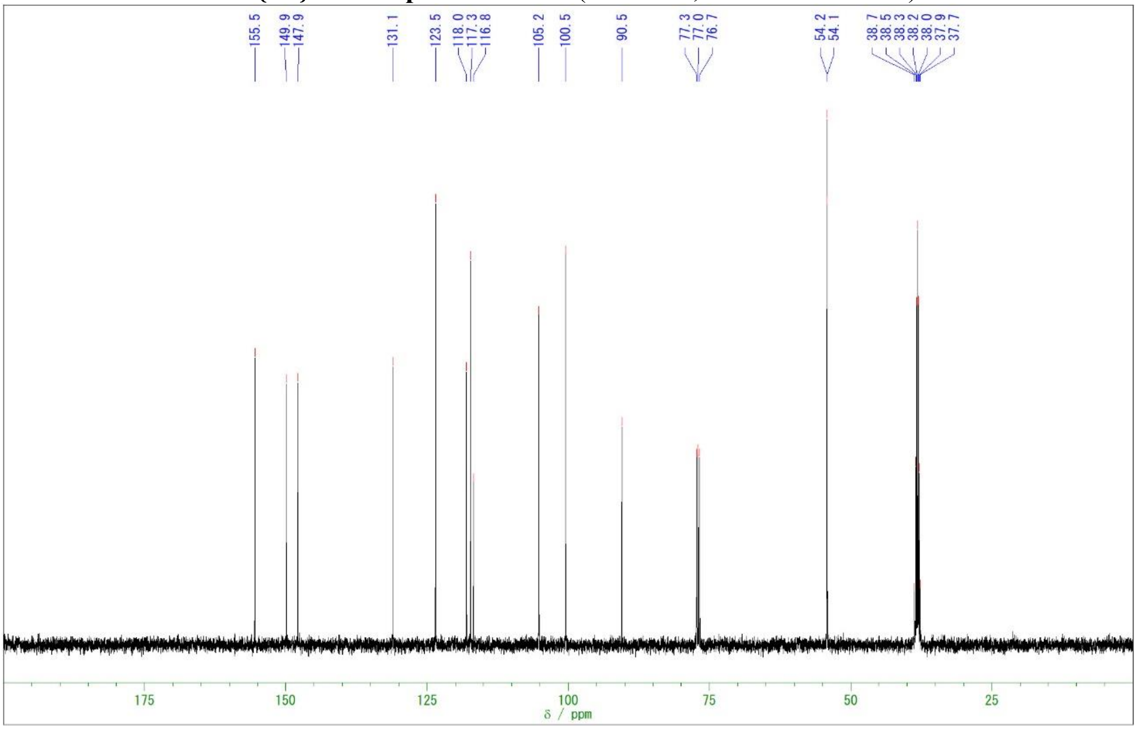

^{1}H NMR Spectrum of 3d (500 MHz, $CDCl_3$)

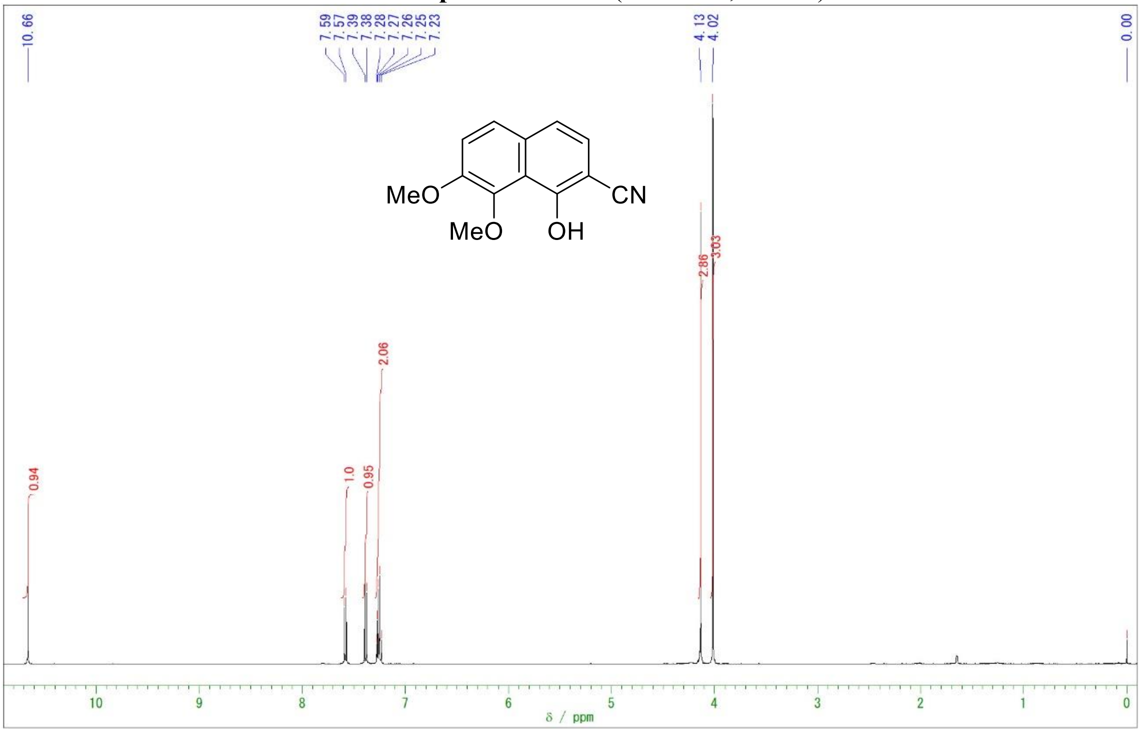

^{13}C{^{1}H} NMR Spectrum of 3d (125 MHz, $CDCl_3$)

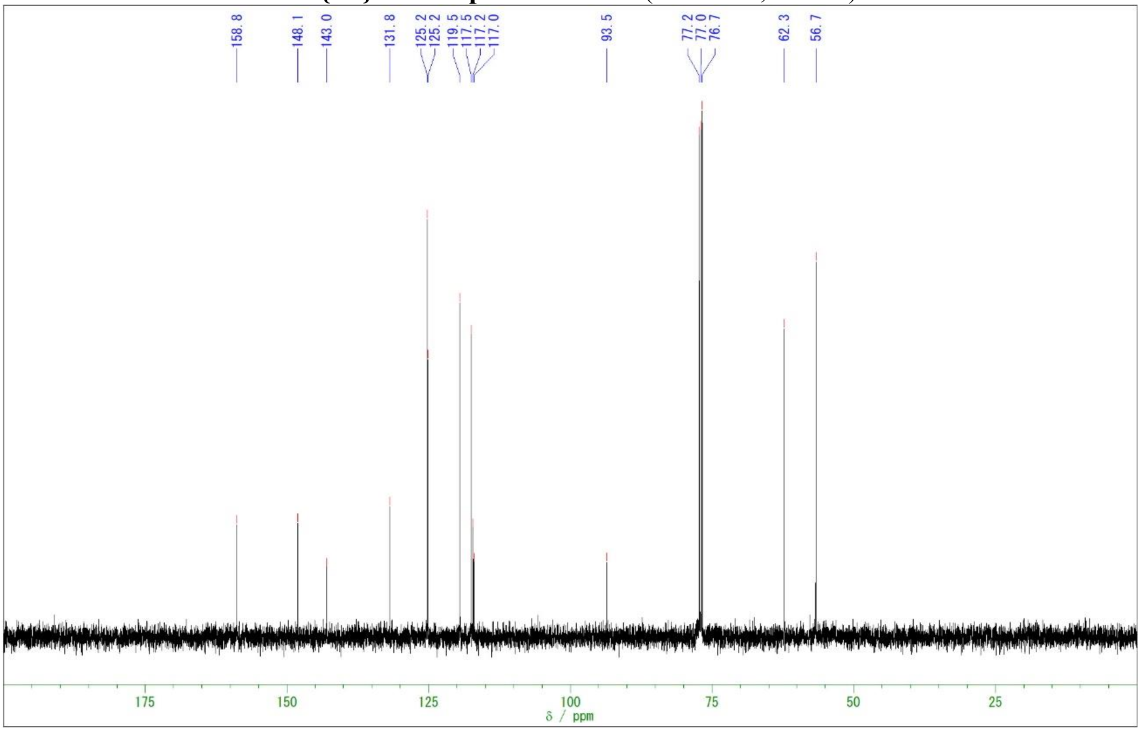

^{1}H NMR Spectrum of 4d (500 MHz, $CDCl_3$)

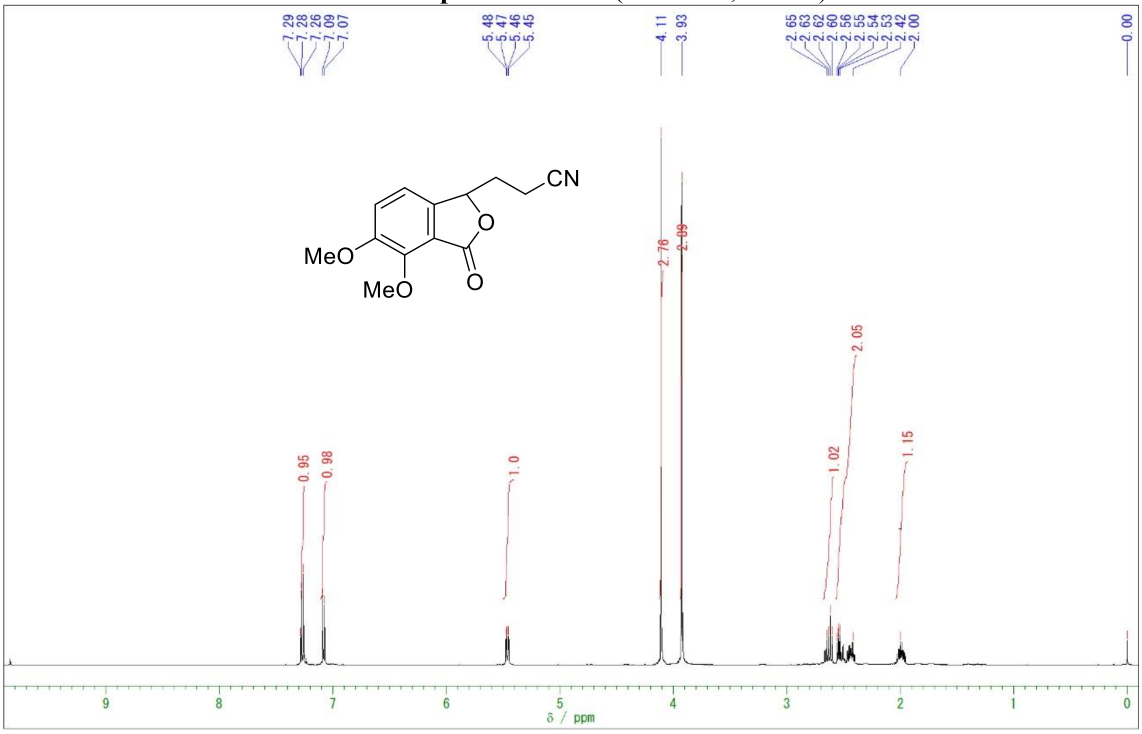

^{13}C{^{1}H} NMR Spectrum of 4d (125 MHz, $CDCl_3$)

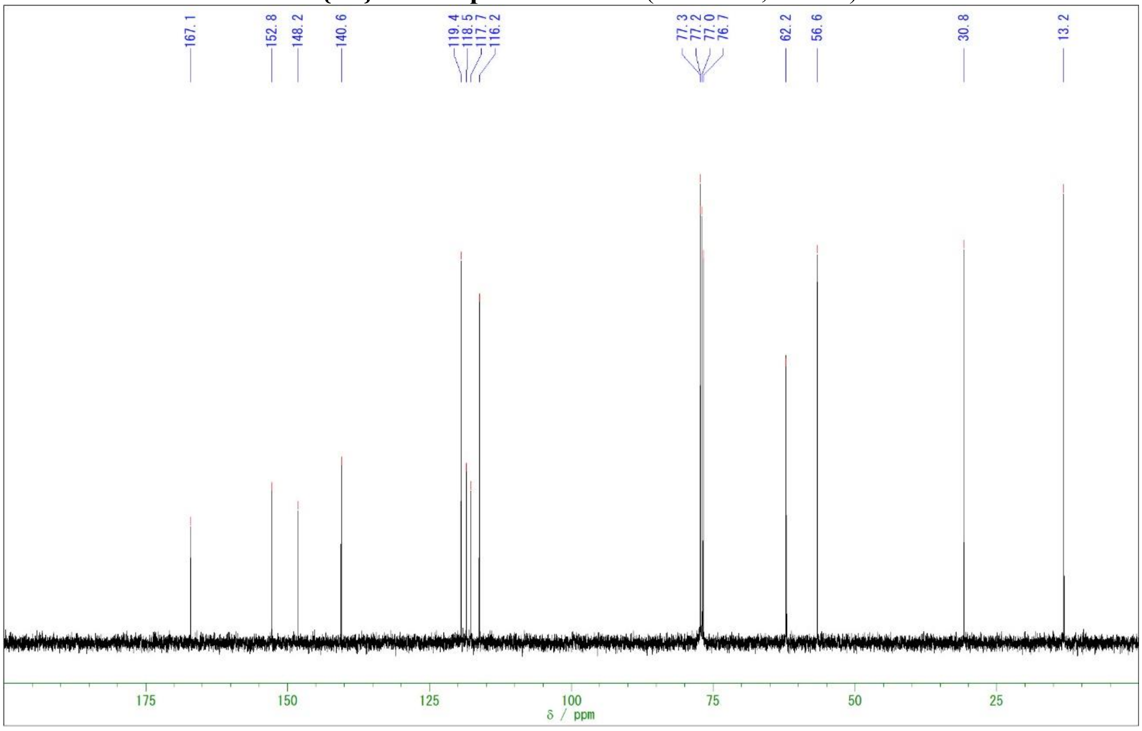

^{1}H NMR Spectrum of 4e (500 MHz, $CDCl_3$)

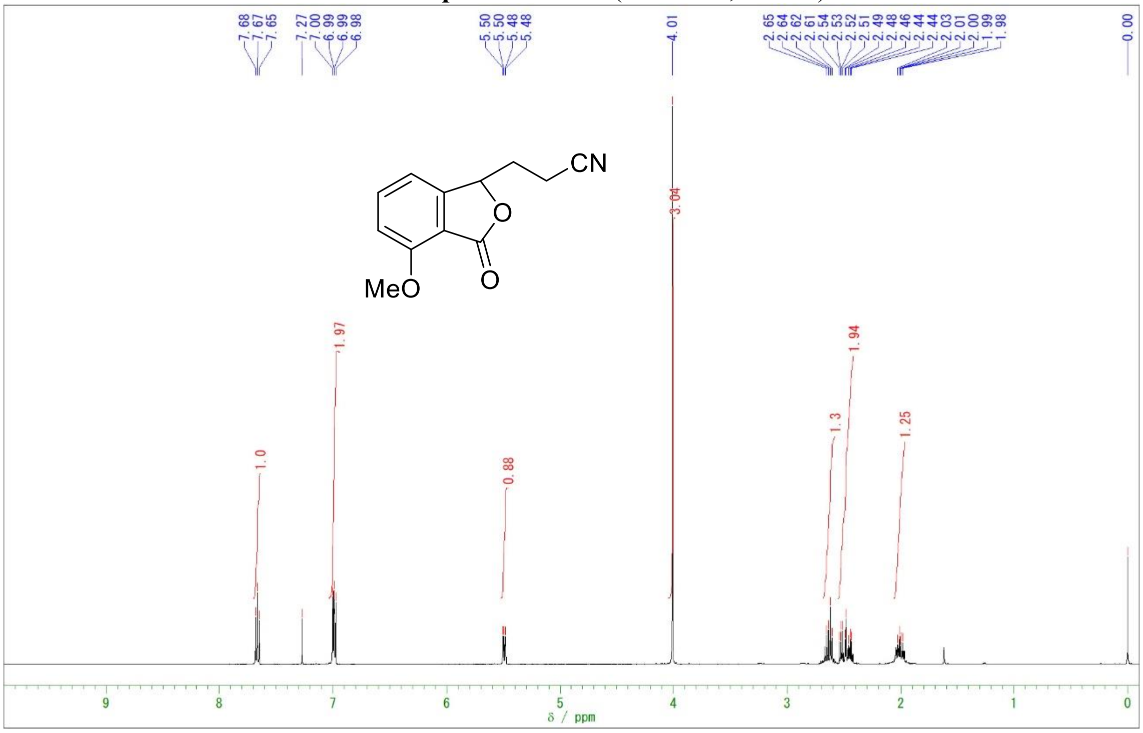

$^{13}C\{^{1}H\}$ NMR Spectrum of 4e (125 MHz, $CDCl_3$)

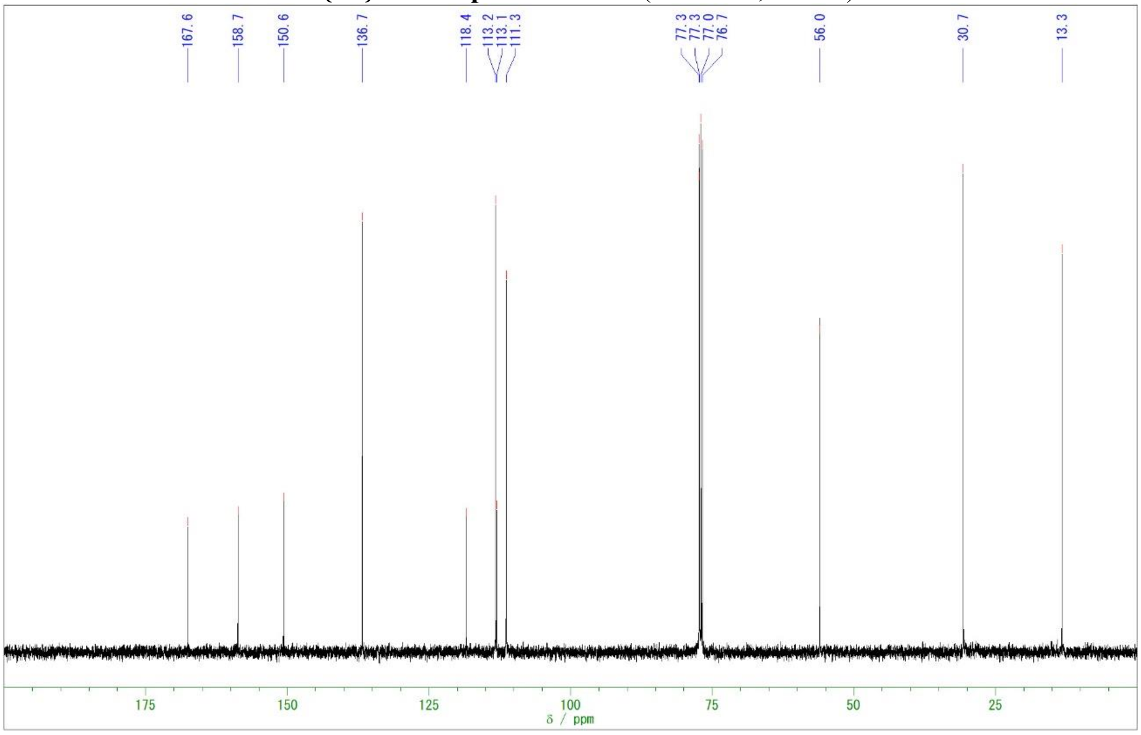

^{1}H NMR Spectrum of 4f (500 MHz, $CDCl_3$)

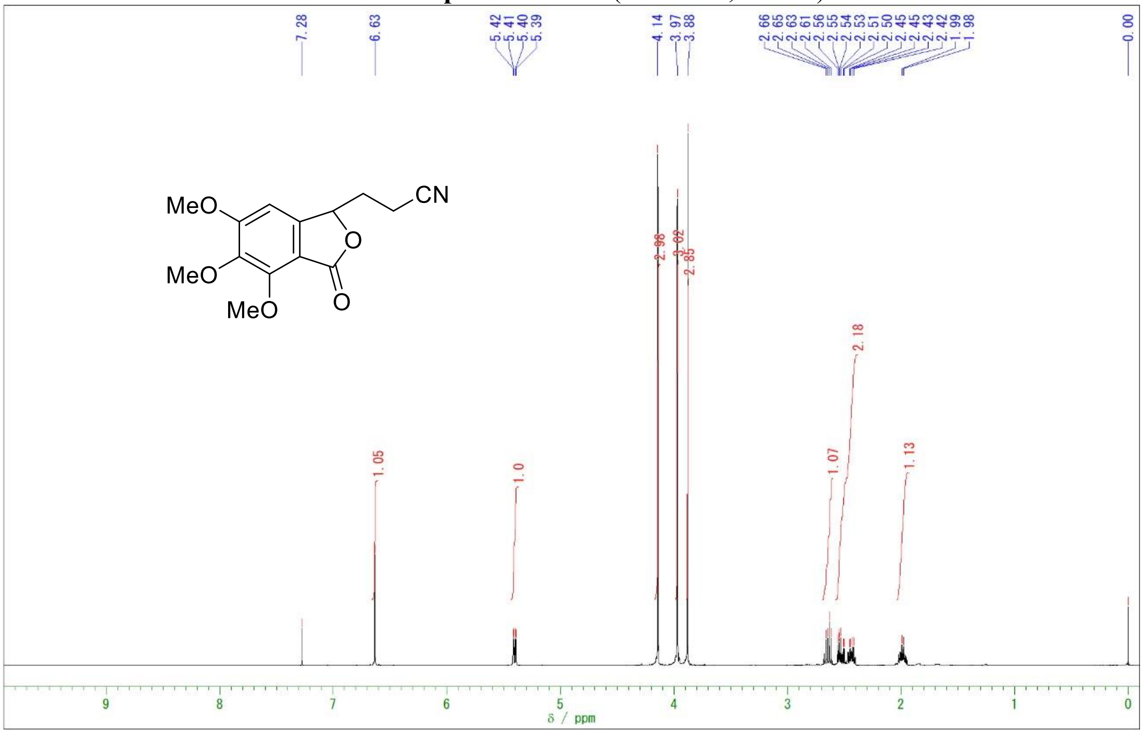

^{13}C{^{1}H} NMR Spectrum of 4f (125 MHz, $CDCl_3$)

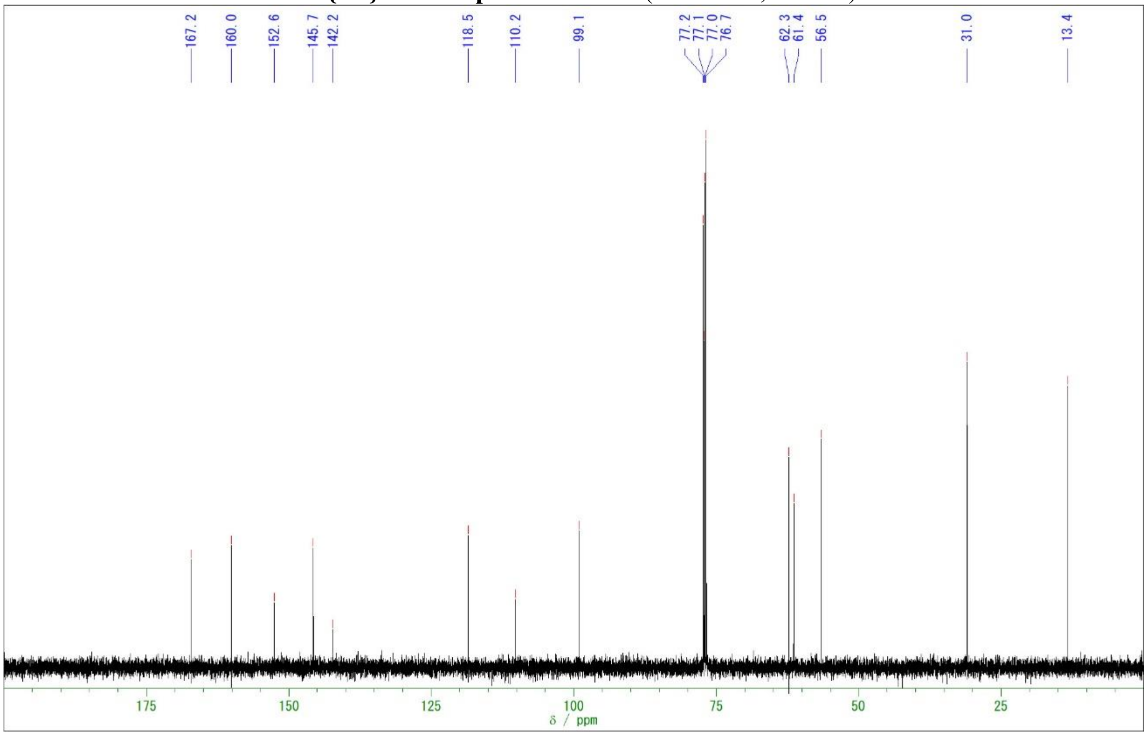

^{1}H NMR Spectrum of 3g (500 MHz, $CDCl_3$)

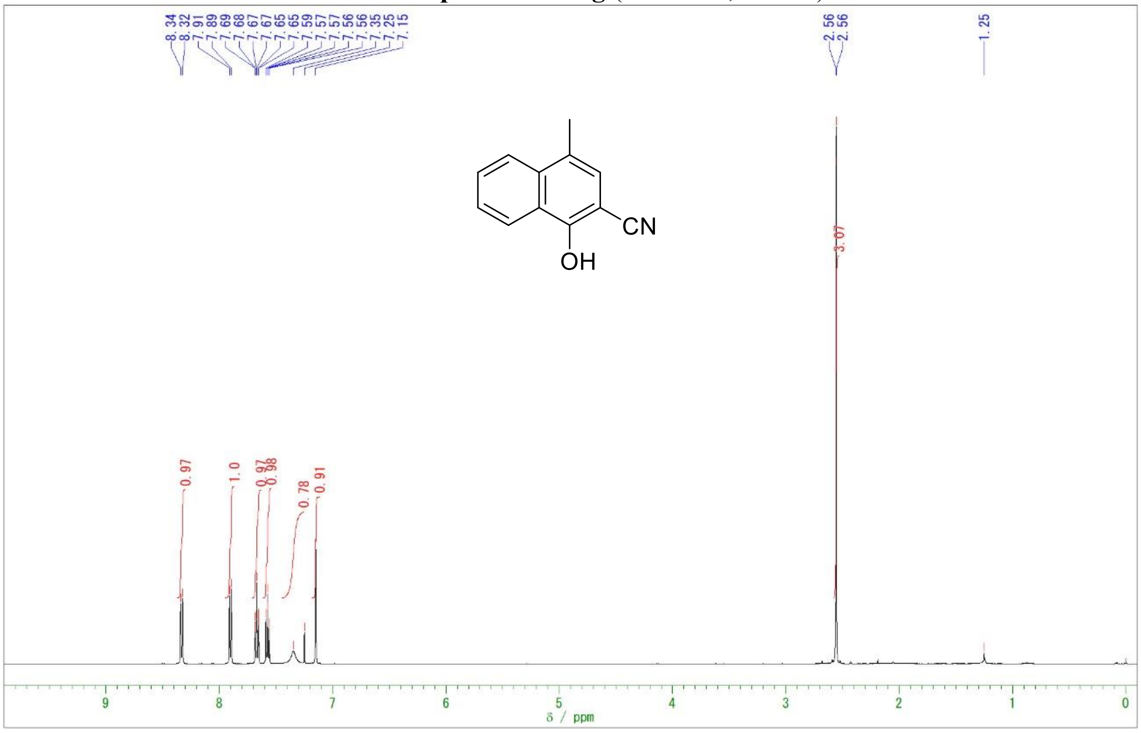

^{13}C{^{1}H} NMR Spectrum of 3g (125 MHz, $CDCl_3$)

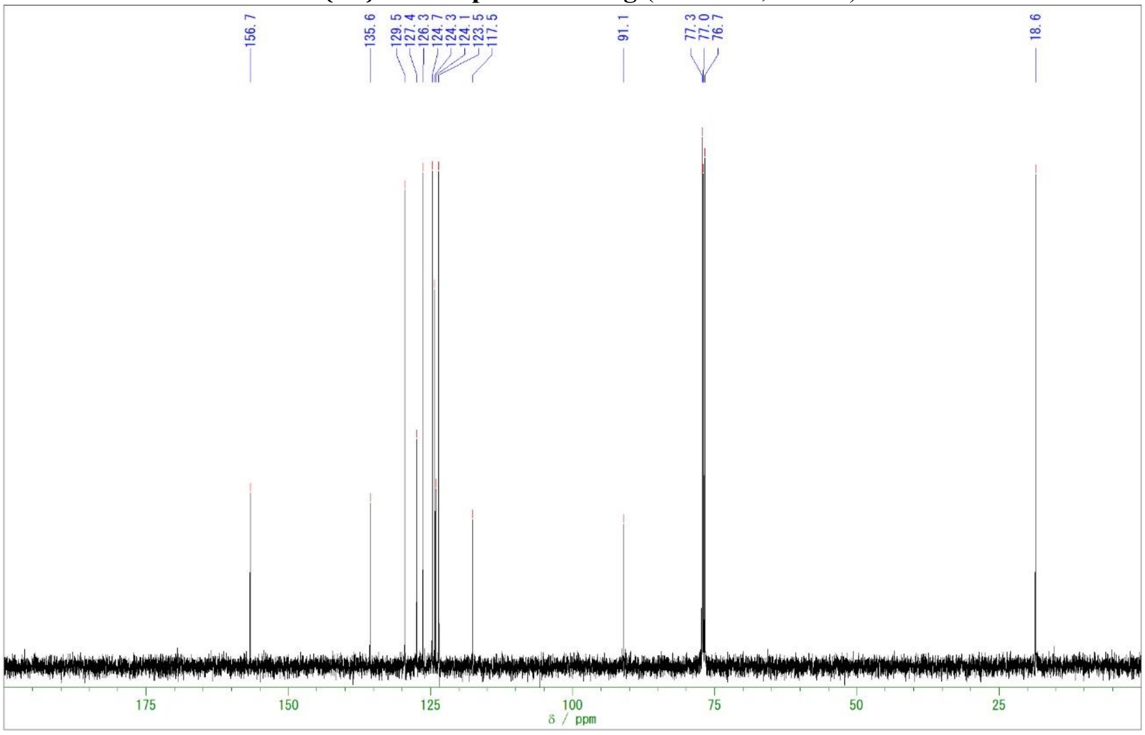

^{1}H NMR Spectrum of 4h (500 MHz, $CDCl_3$)

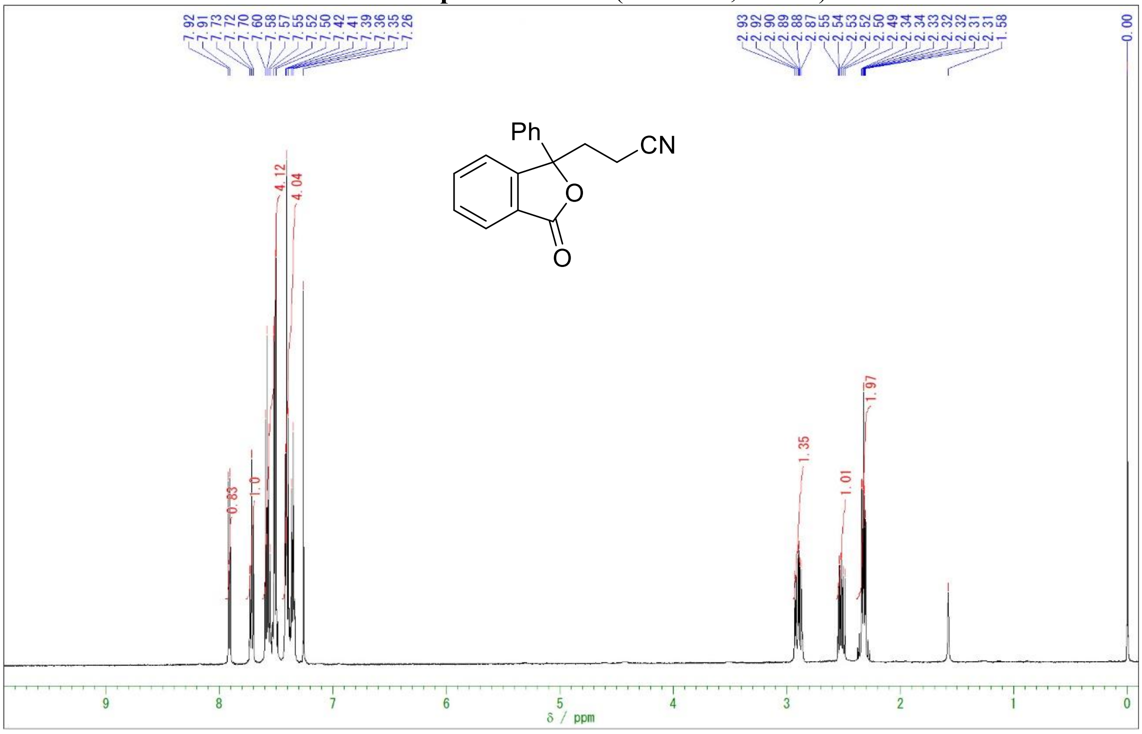

^{13}C{^{1}H} NMR Spectrum of 4h (125 MHz, $CDCl_3$)

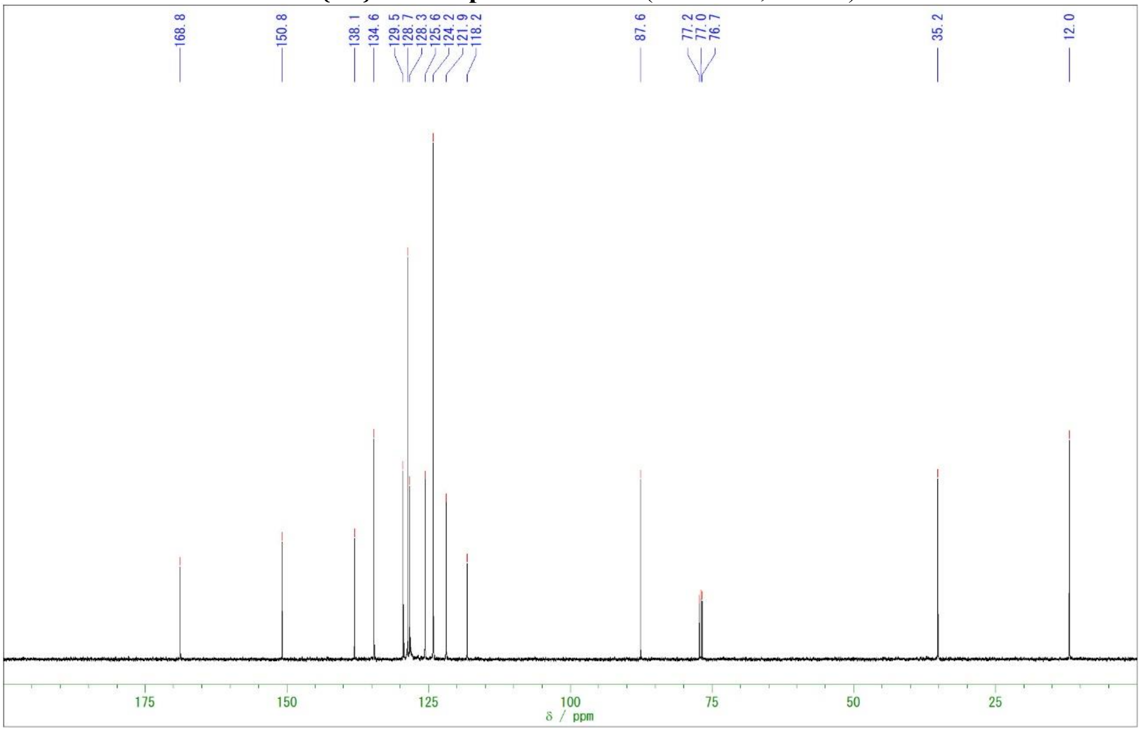

^{1}H NMR Spectrum of i (500 MHz, $CDCl_3$)

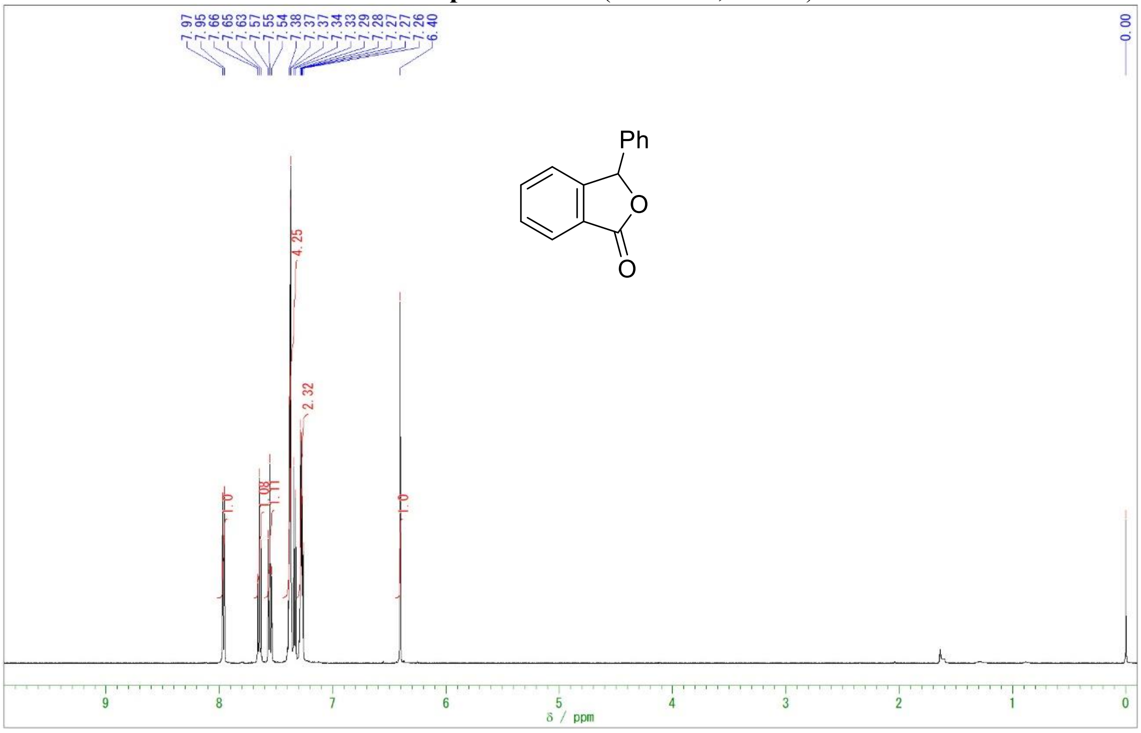

^{13}C{^{1}H} NMR Spectrum of i (125 MHz, $CDCl_3$)

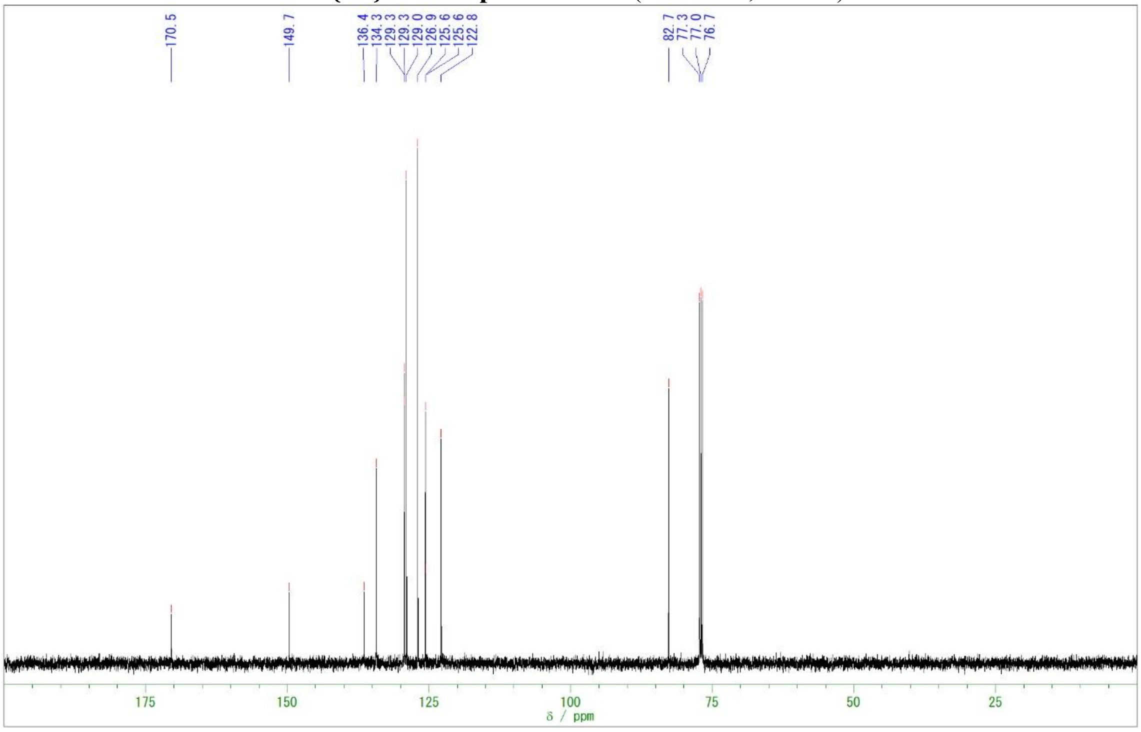

^{1}H NMR Spectrum of 5a (500 MHz, $CDCl_3$)

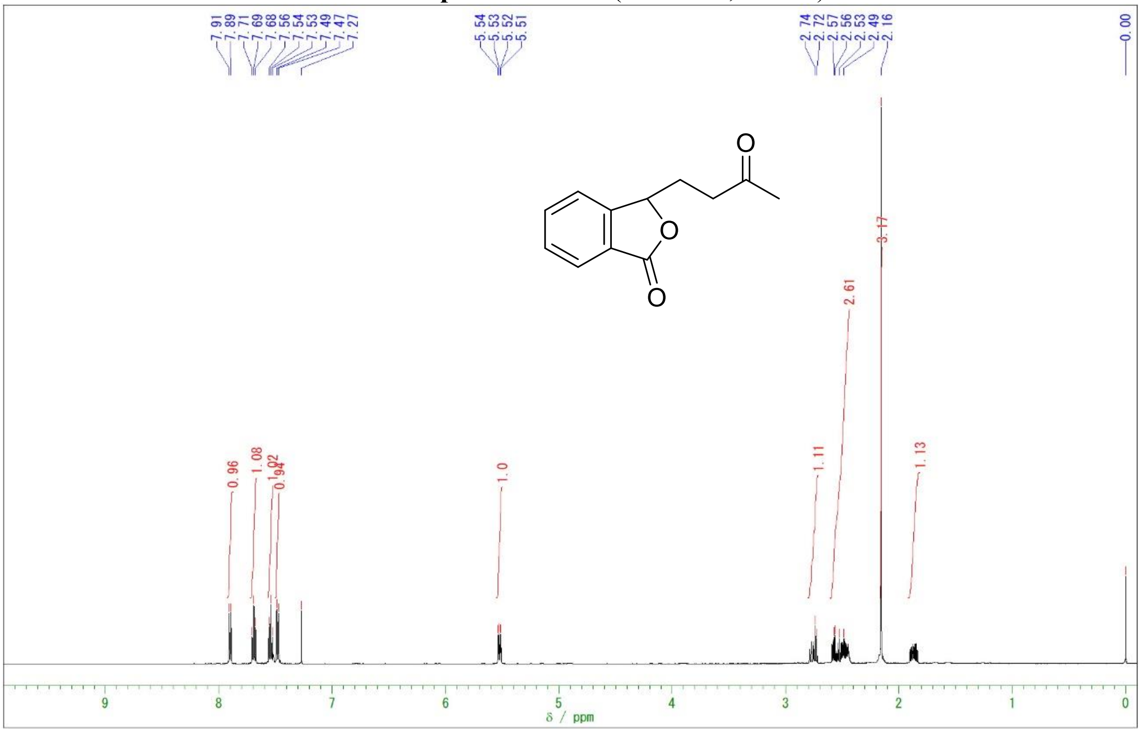

^{13}C{^{1}H} NMR Spectrum of 5a (125 MHz, $CDCl_3$)

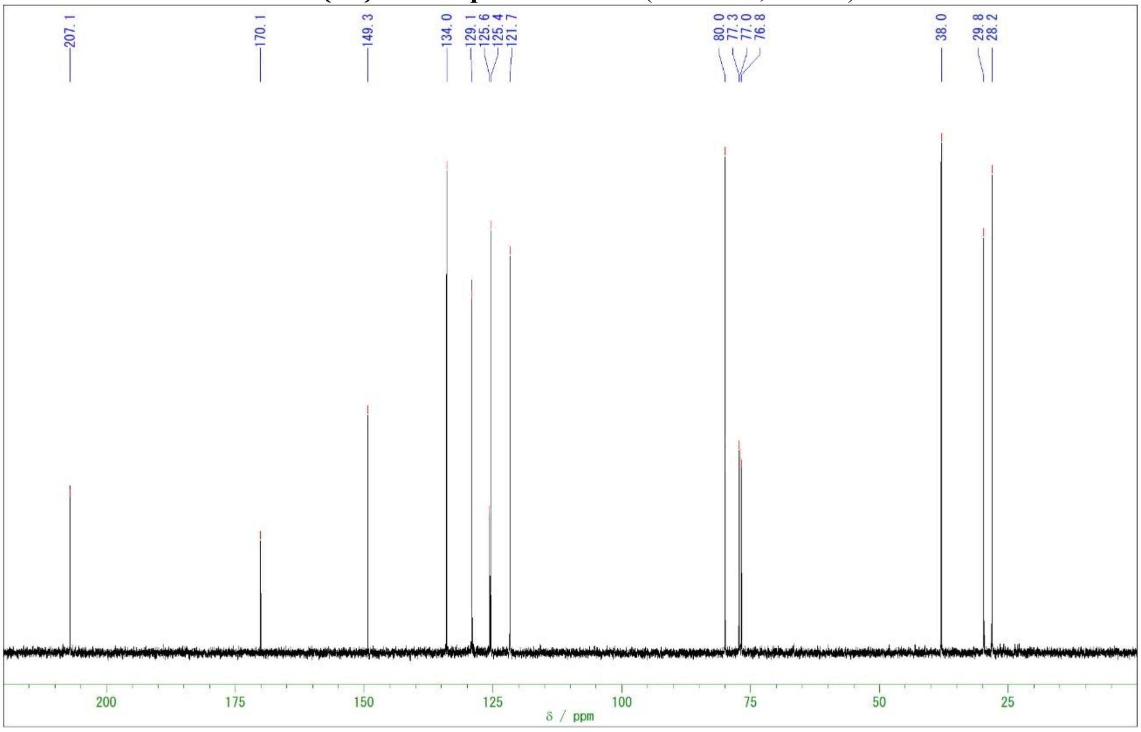

^{1}H NMR Spectrum of 5b and octane-2,7-dione (1:0.17 mixture) (500 MHz, $CDCl_3$)

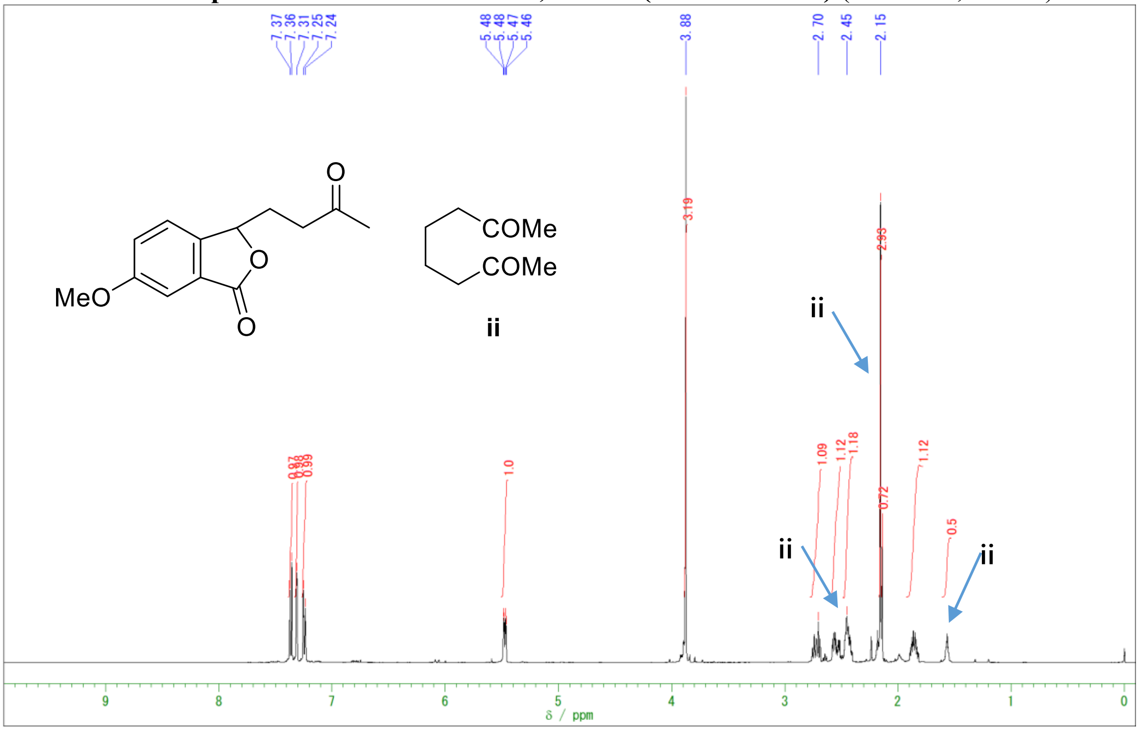

^{13}C{^{1}H} NMR Spectrum of 5b and octane-2,7-dione (1:0.17 mixture) (125 MHz, $CDCl_3$)

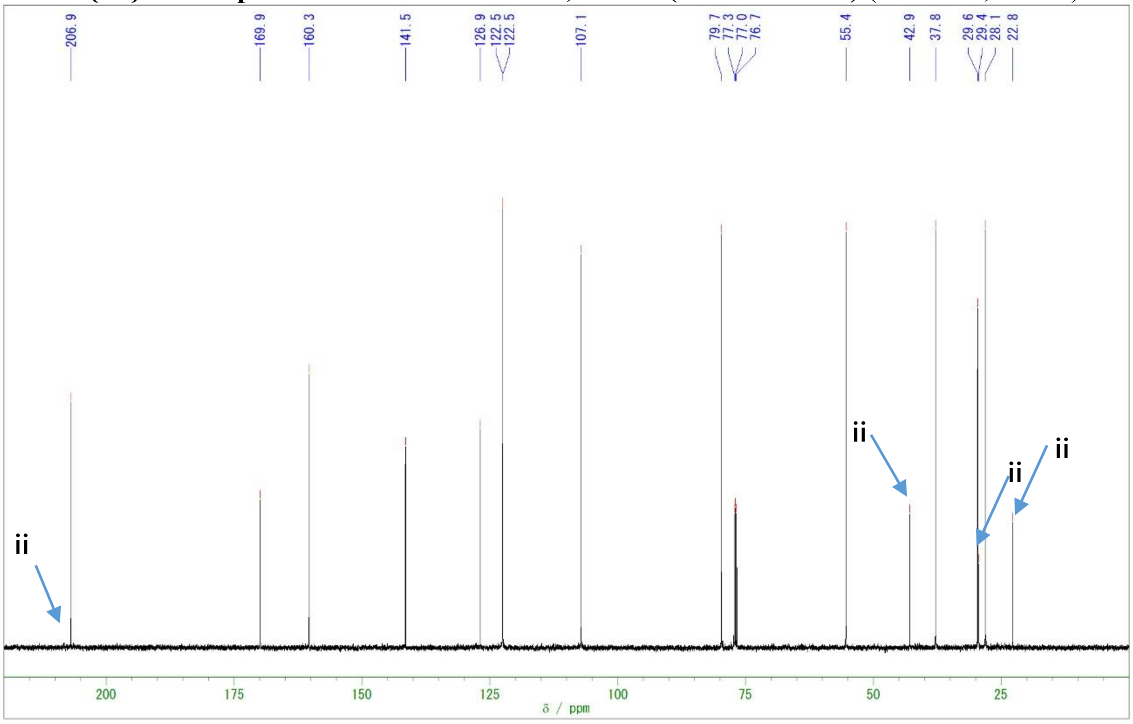

^{1}H NMR Spectrum of 5c (500 MHz, $CDCl_3$)

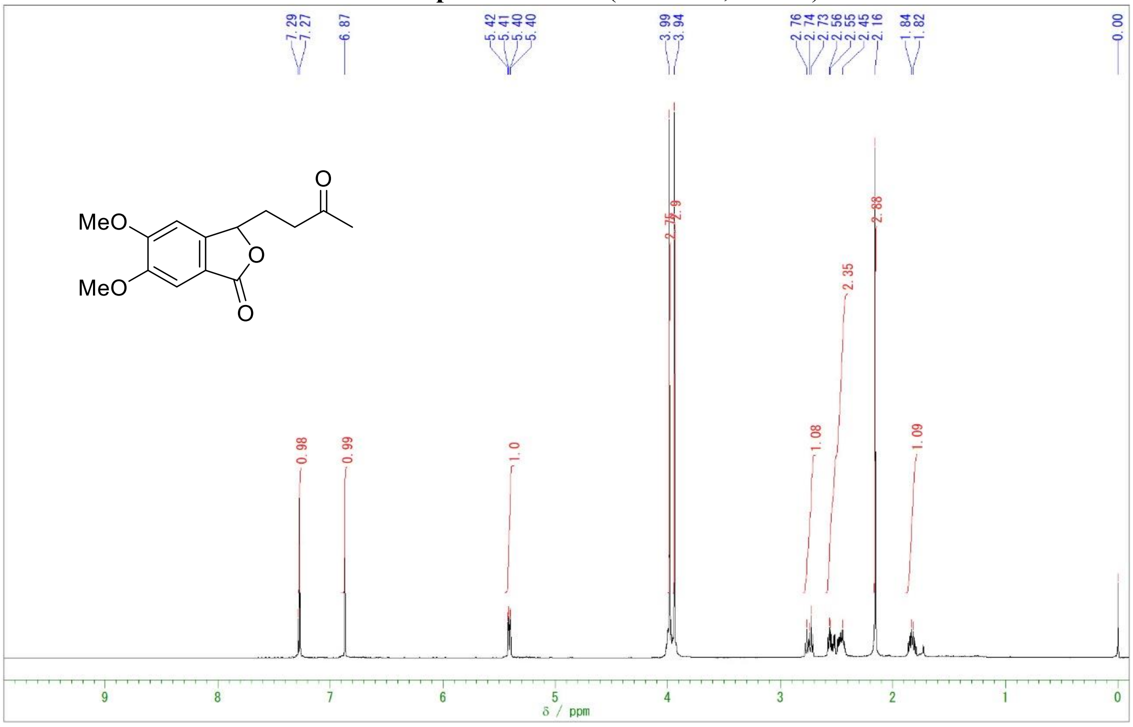

^{13}C{^{1}H} NMR Spectrum of 5c (125 MHz, $CDCl_3$)

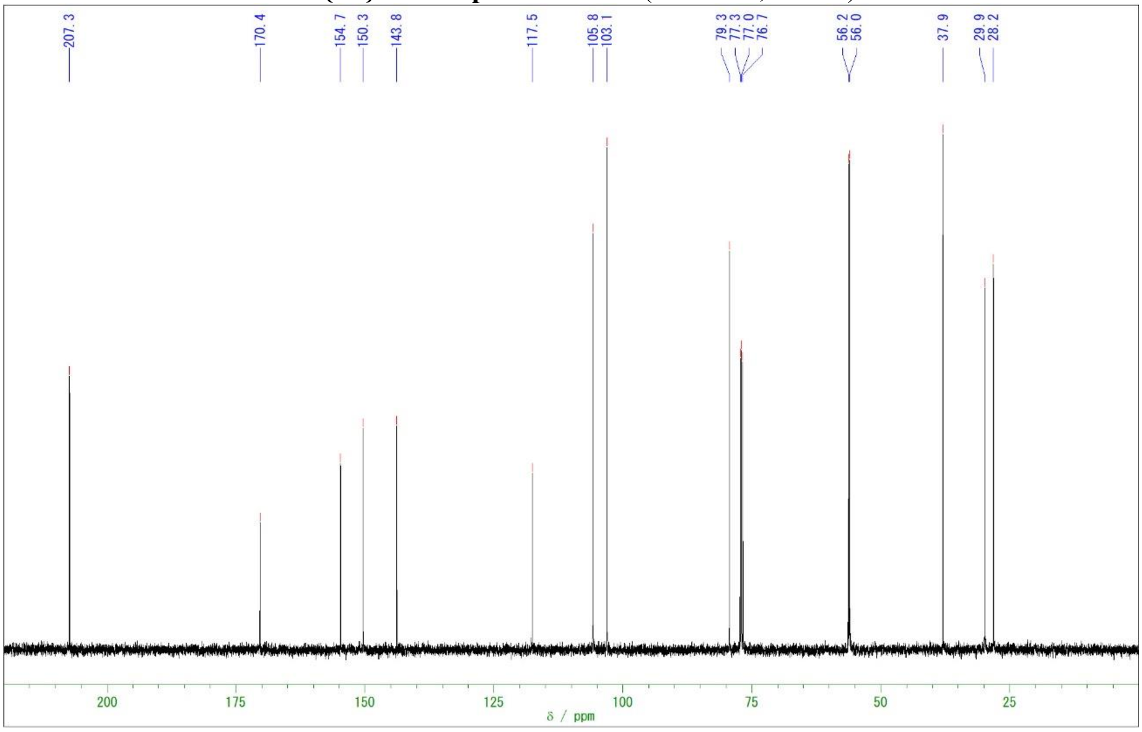

^{1}H NMR Spectrum of 5d (500 MHz, $CDCl_3$)

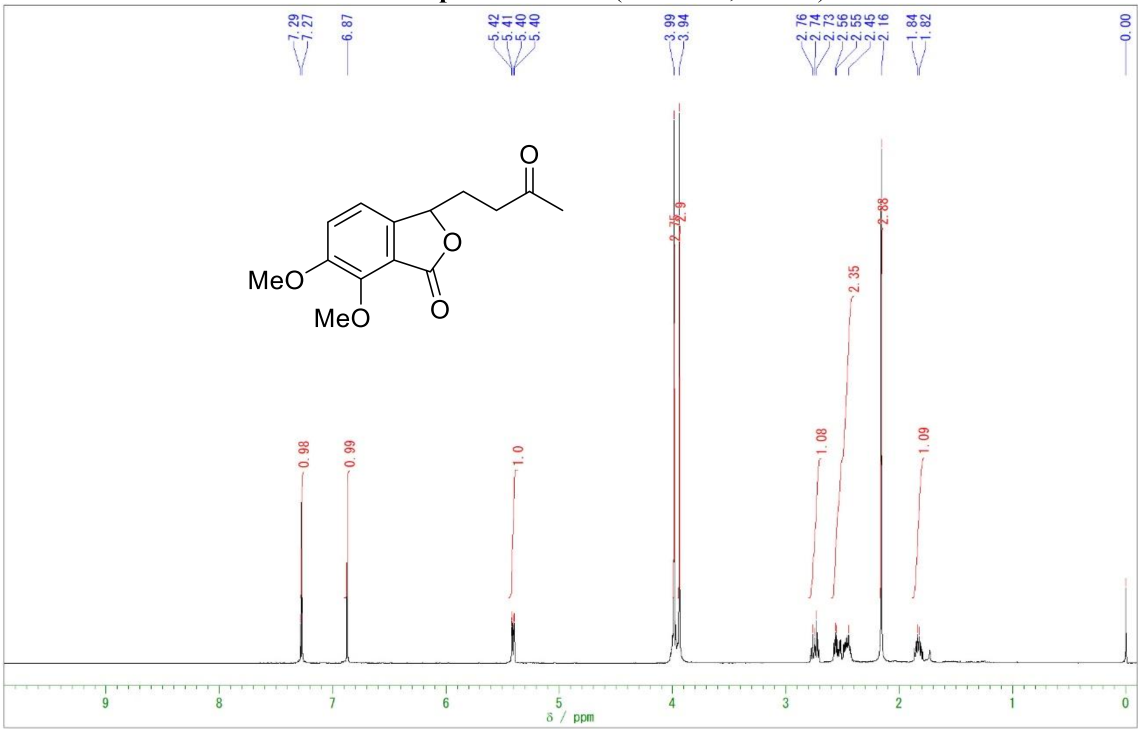

^{13}C{^{1}H} NMR Spectrum of 5d (125 MHz, $CDCl_3$ + *d6*DMSO)

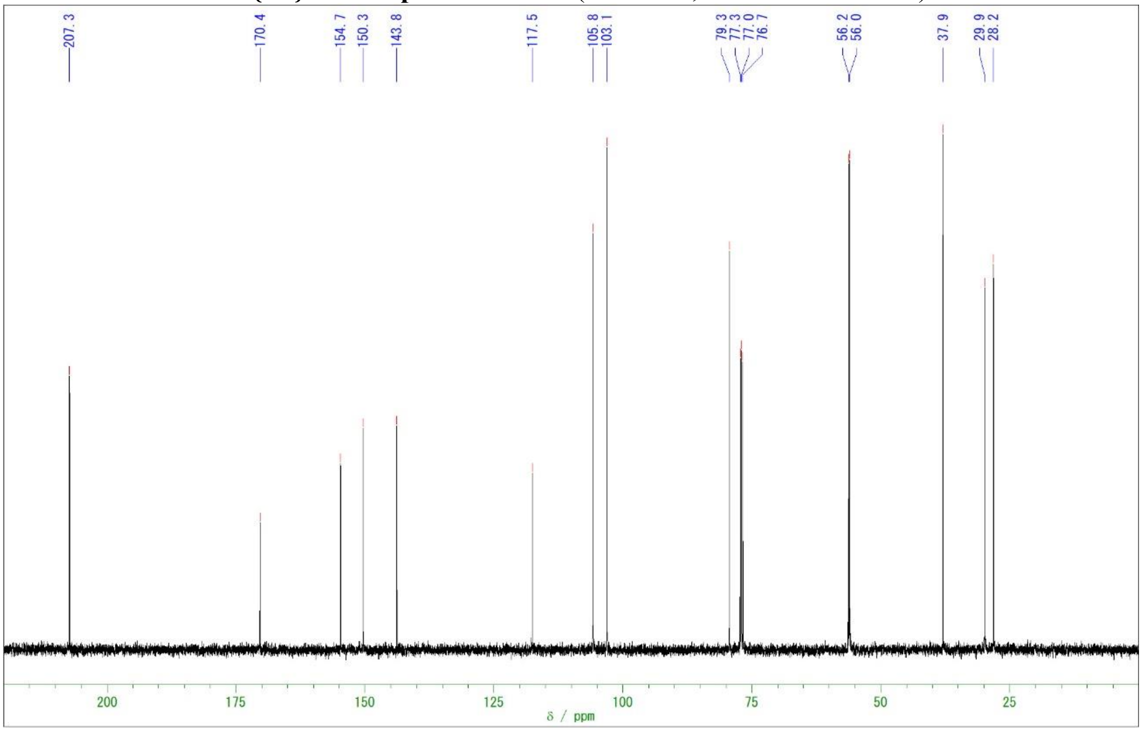

^{1}H NMR Spectrum of 5e (500 MHz, $CDCl_3$)

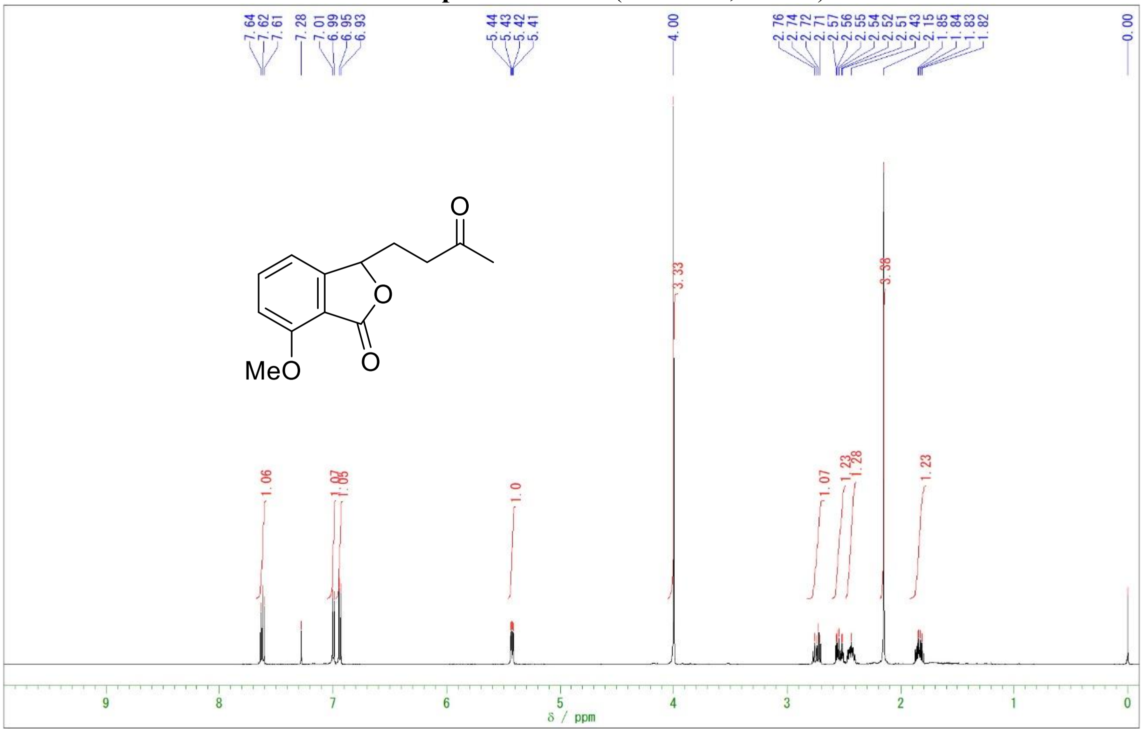

$^{13}C\{^{1}H\}$ NMR Spectrum of 5e (125 MHz, $CDCl_3$)

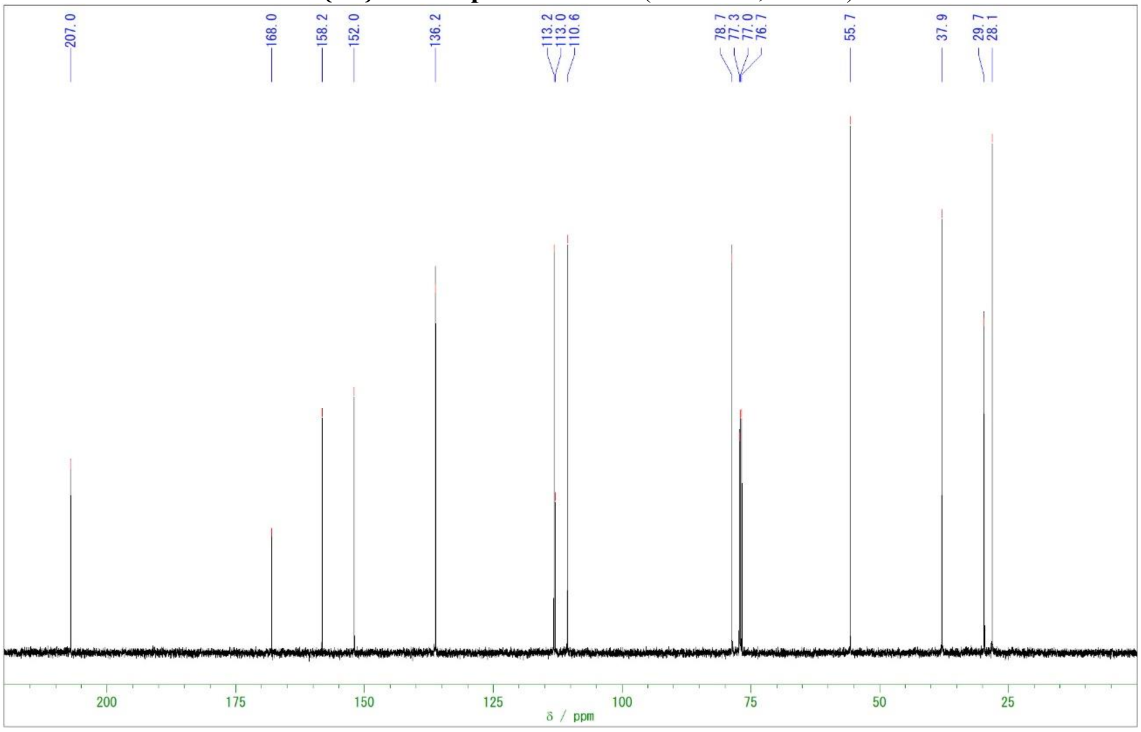

^{1}H NMR Spectrum of 5f (500 MHz, $CDCl_3$)

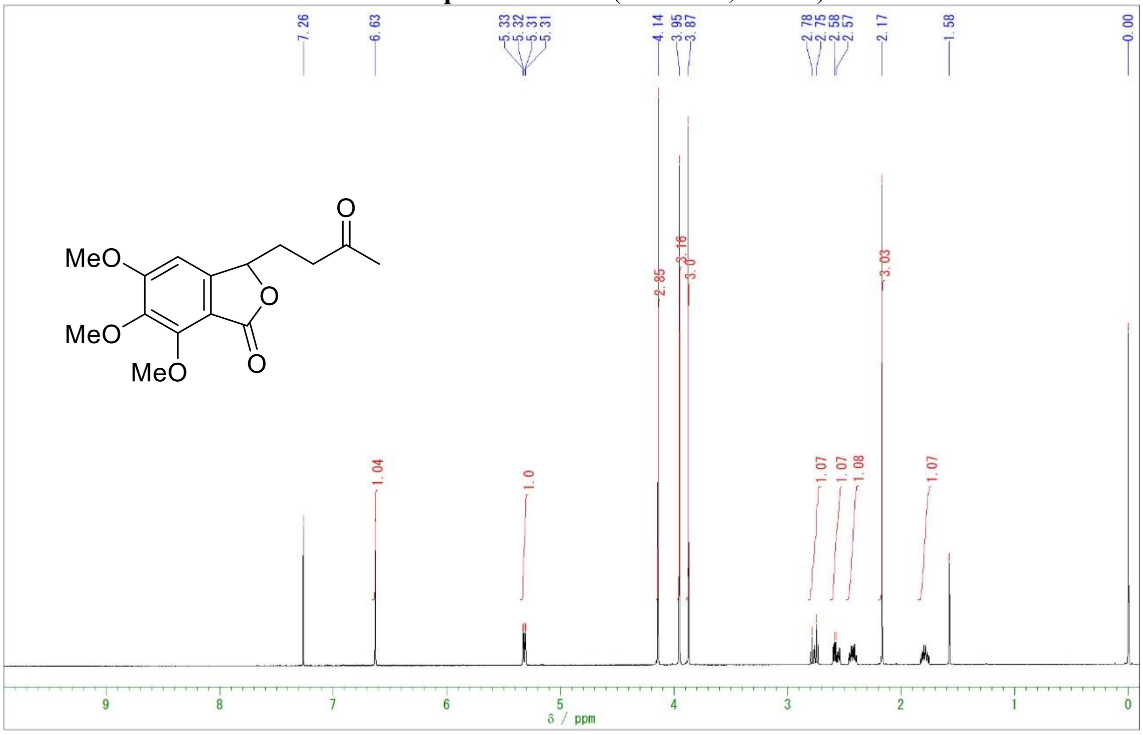

^{13}C{^{1}H} NMR Spectrum of 5f (125 MHz, $CDCl_3$)

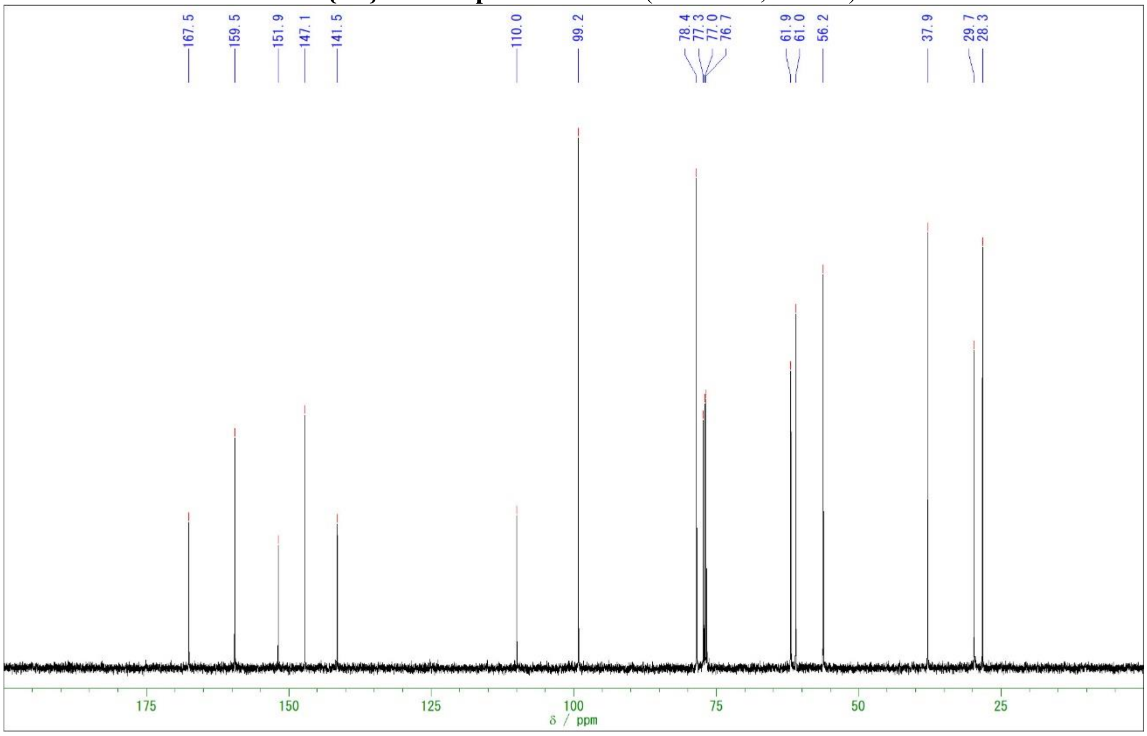

^{1}H NMR Spectrum of 5g and octane-2,7-dione (1:0.3 mixture) (500 MHz, $CDCl_3$)

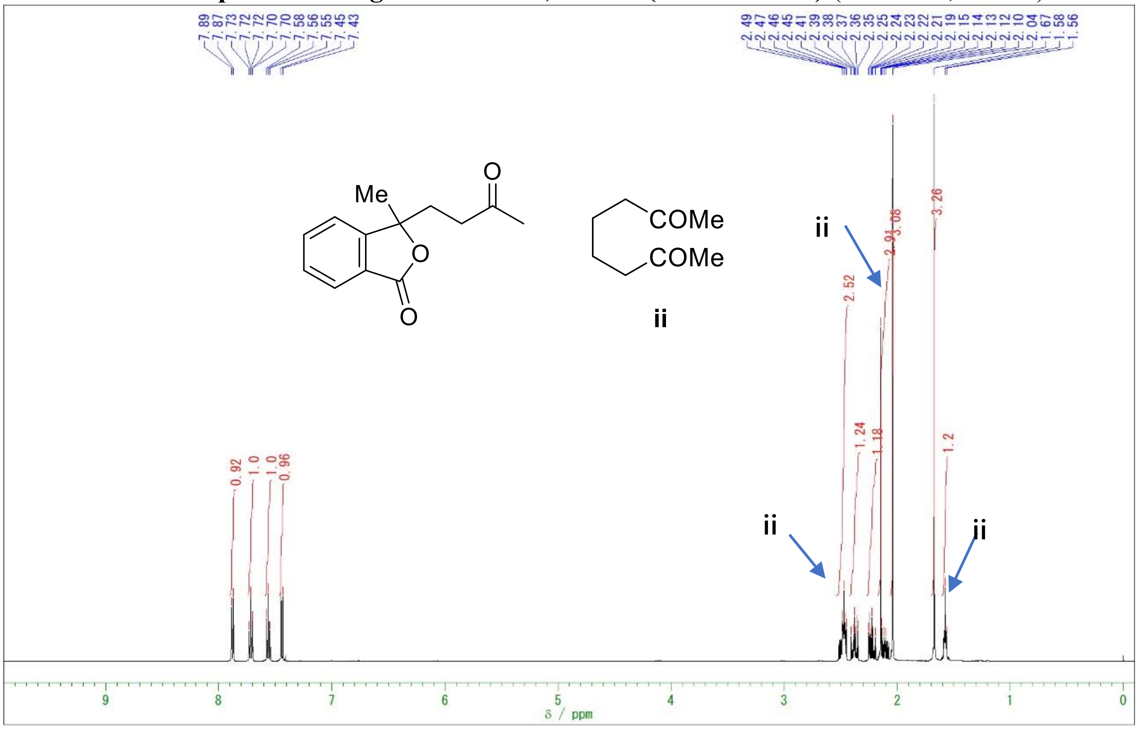

$^{13}C\{^{1}H\}$ NMR Spectrum of 5g and octane-2,7-dione (1:0.3 mixture) (125 MHz, $CDCl_3$)

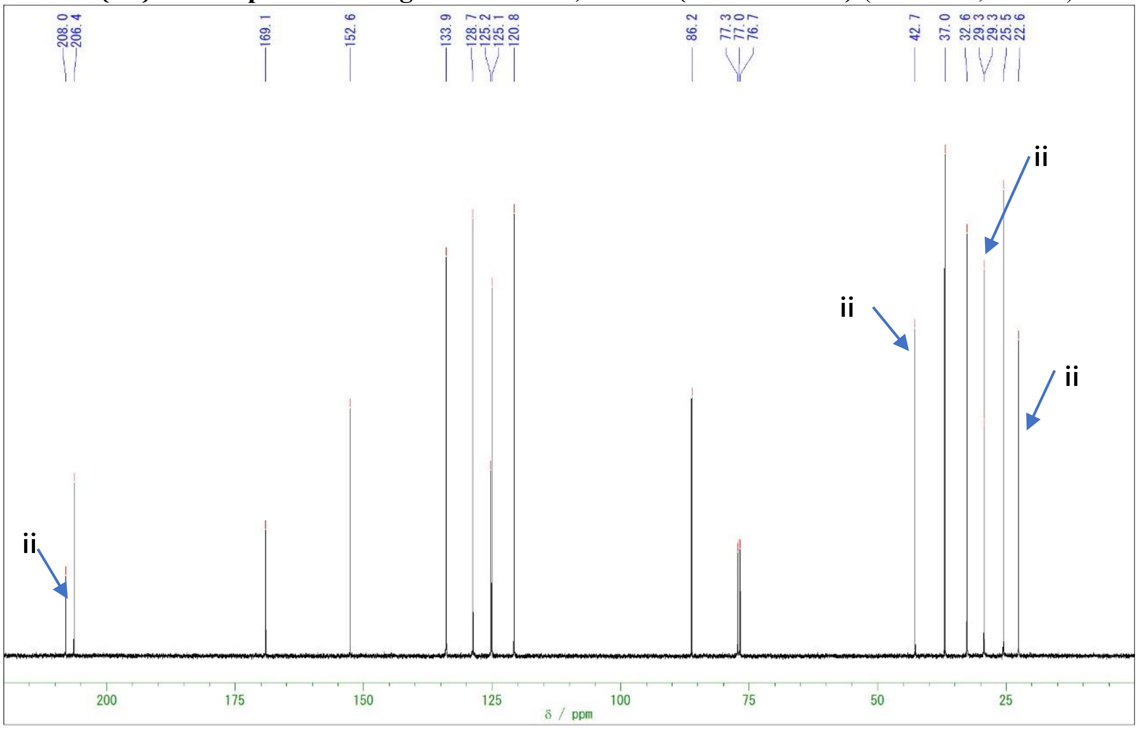

¹H NMR Spectrum of 5h (500 MHz, $CDCl_3$)

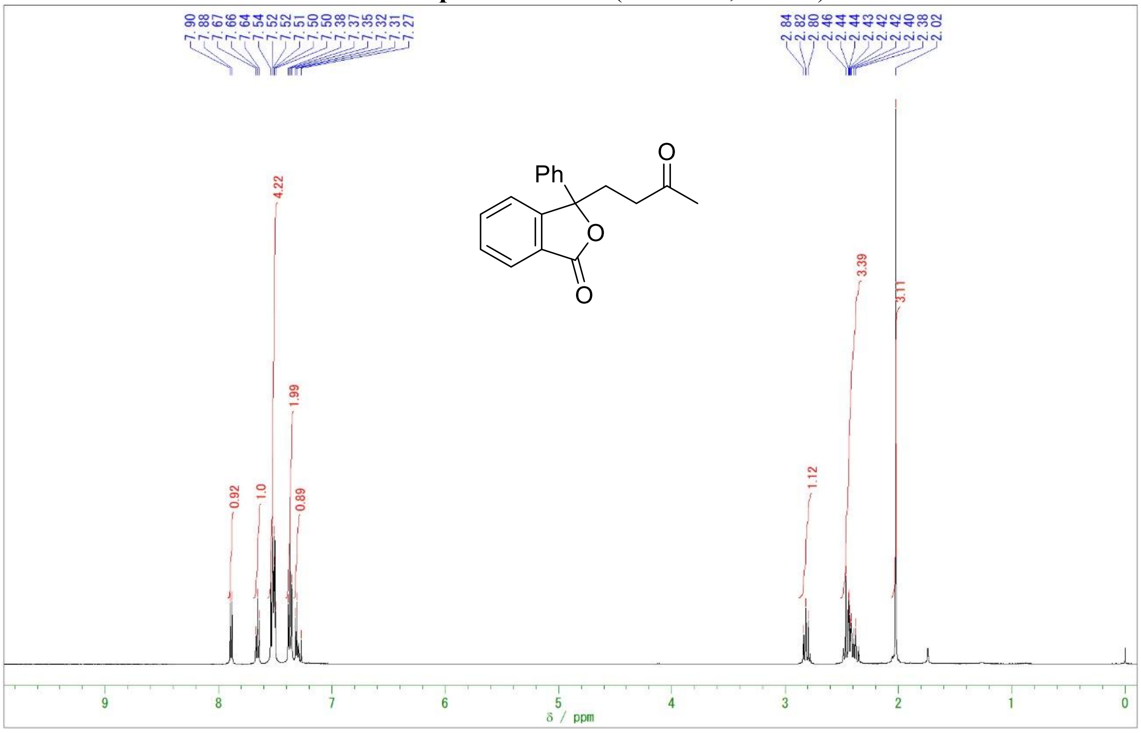

¹³C{¹H} NMR Spectrum of 5h (125 MHz, $CDCl_3$)

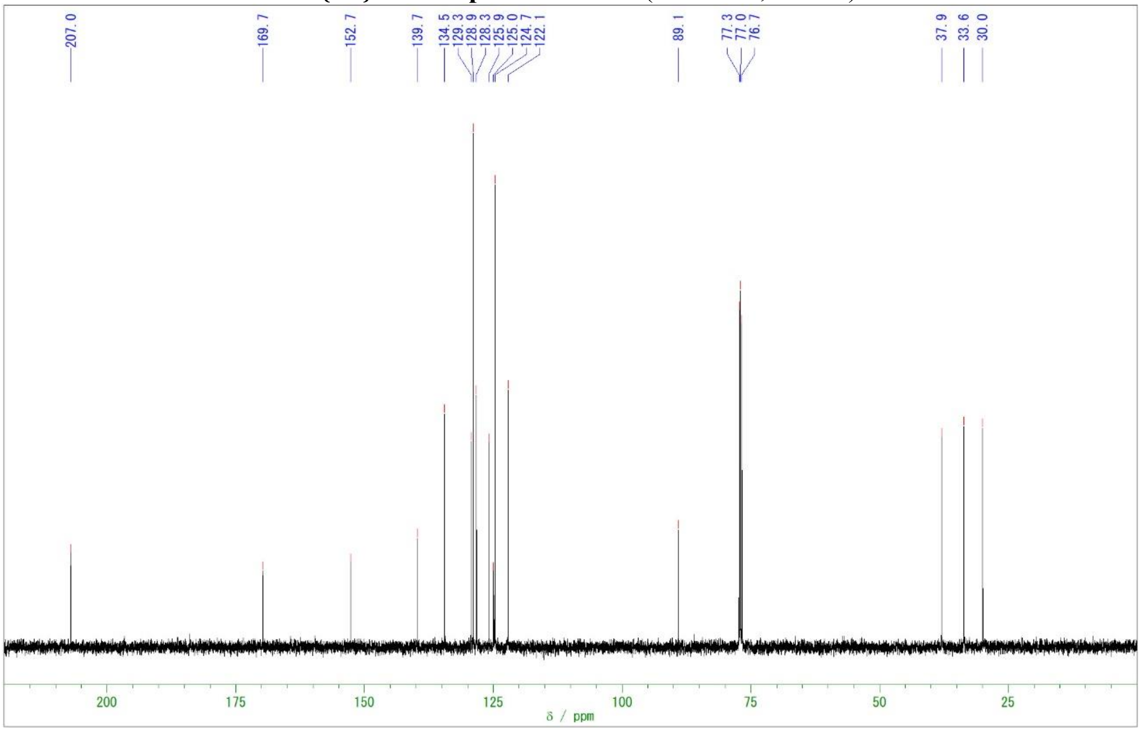

^{1}H NMR Spectrum of 8 (500 MHz, $CDCl_3$)

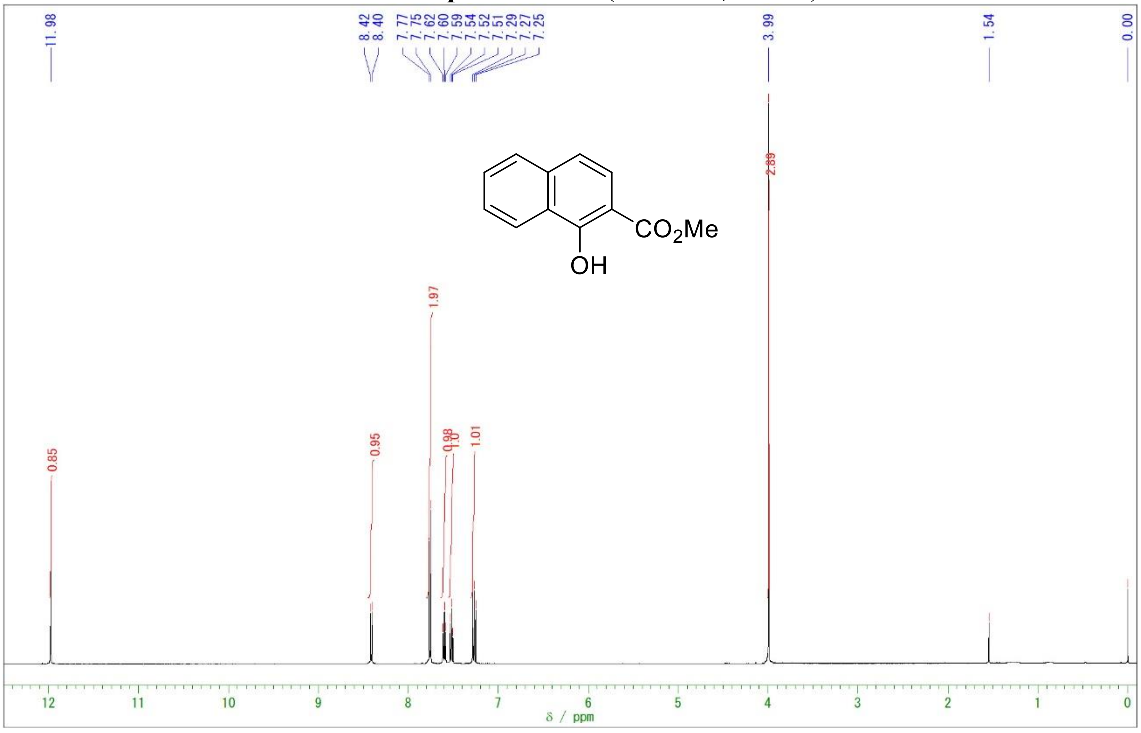

$^{13}C\{^{1}H\}$ NMR Spectrum of 8 (125 MHz, $CDCl_3$)

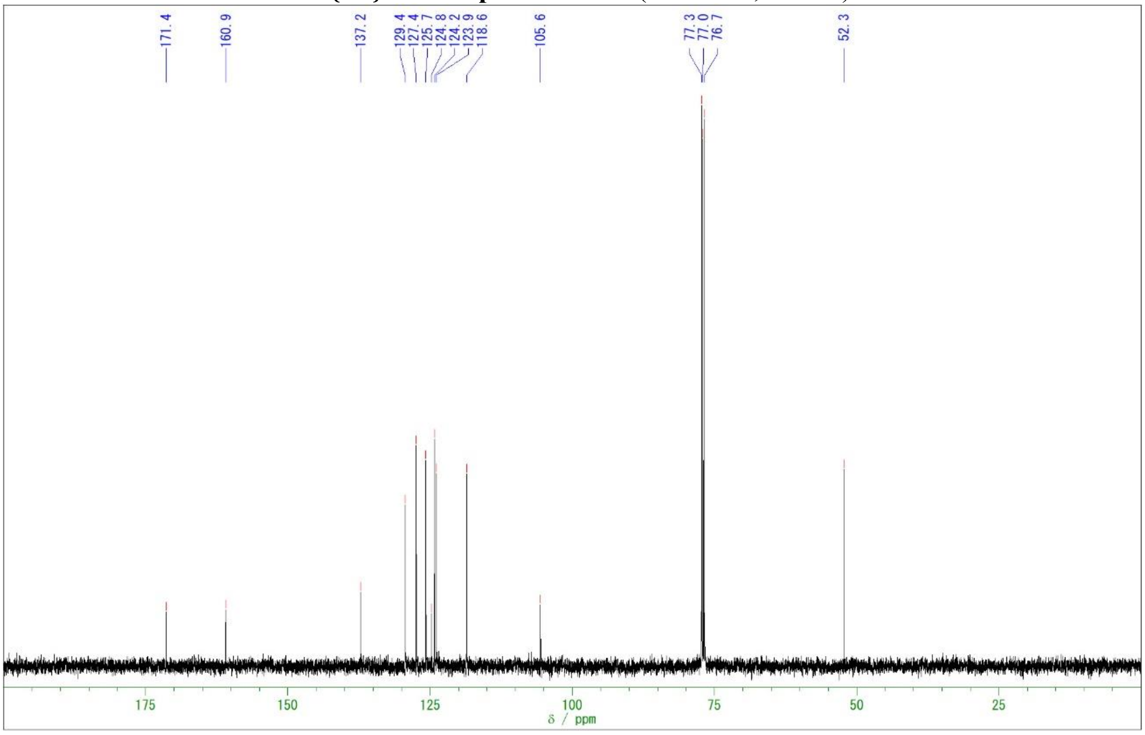

^{1}H NMR Spectrum of 9 (65:35 dr) (500 MHz, $CDCl_3$/DMSO-*d6*)

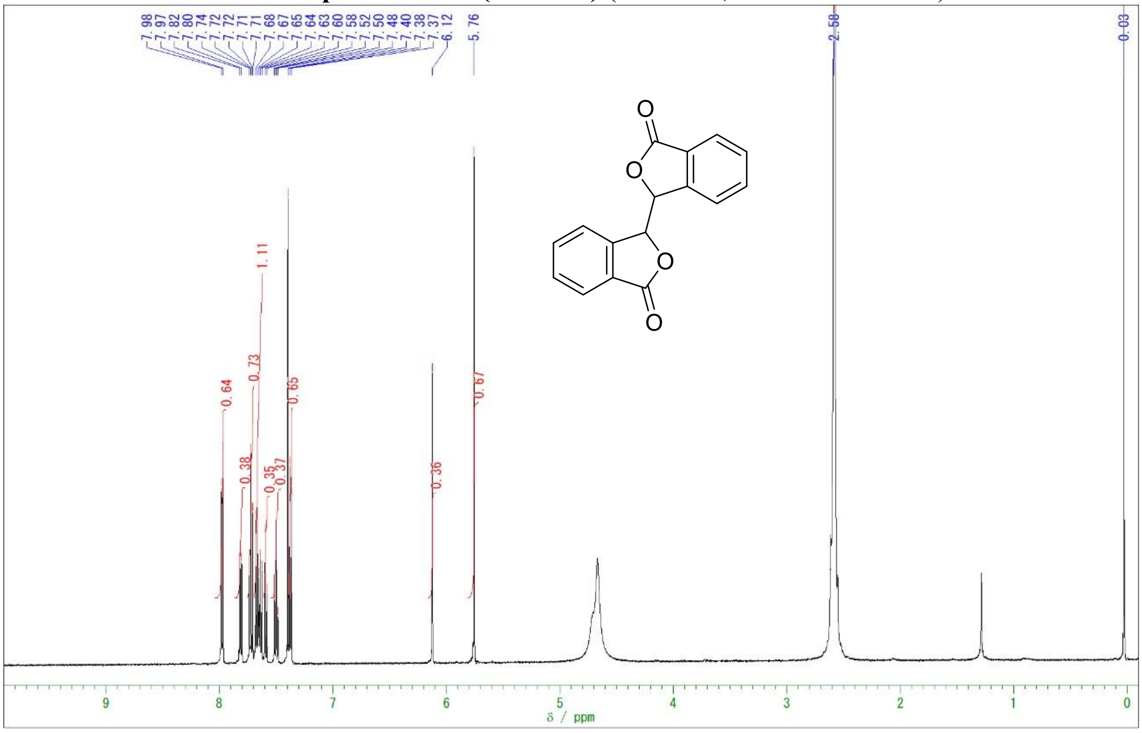

^{13}C{^{1}H} NMR Spectrum of 9 (125 MHz, $CDCl_3$/DMSO-*d6*)

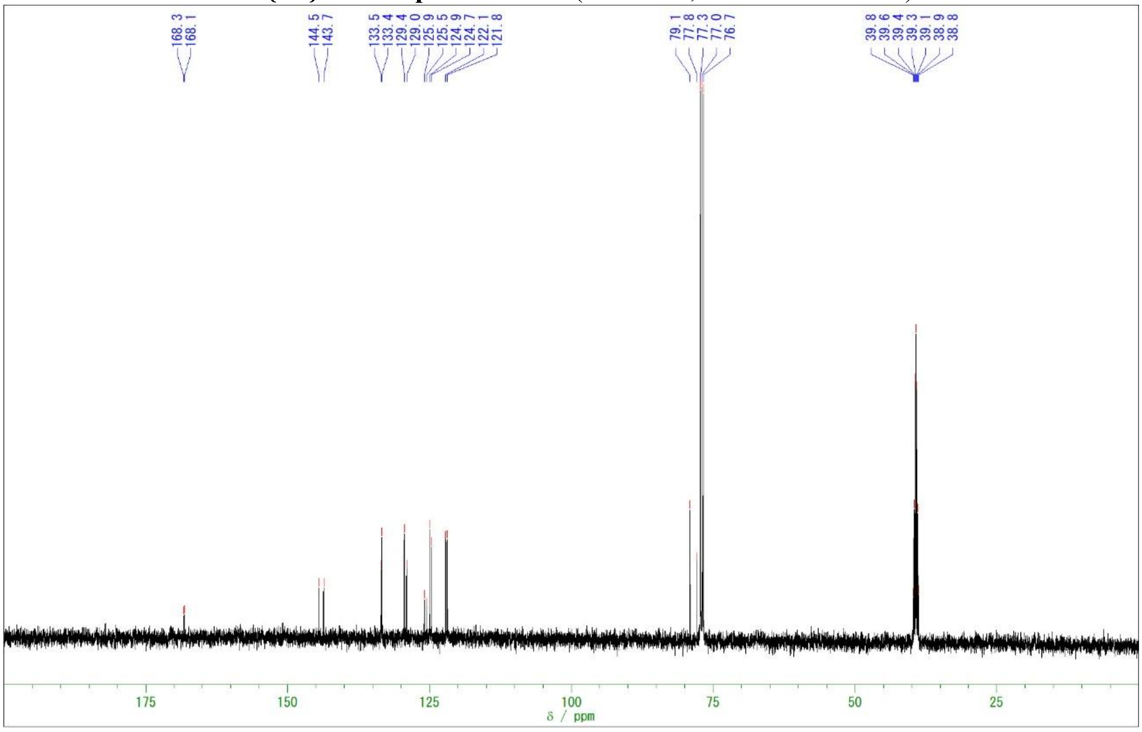

X-ray crystallographic data (ORTEP, 50% probability) of 3b

X-ray crystallographic analysis. All measurements were made on a Rigaku RAXIS imaging plate area detector with graphite monochromated MoKα radiation. The structure was solved by direct methods with SIR-97 and refined with SHELXL-97. The non-hydrogen atoms were refined anisotropically. Hydrogen atoms were refined isotropically. All calculations were performed using the Yadokari-XG software package.

Crystal data of 3b (CCDC: 2160477). $C_{12}H_9NO_2$, FW = 199.20, mp 181-183 ˚C, monoclinic, $P2_{1/n}$ (no 14), colorless block, a = 7.4001(16) Å, b = 11.026(4) Å, c = 12.309(3) Å, β = 96.868(14), V = 997.1(5) Å^3, T = 298 K, Z = 4, D_{calcd} = 1.327 g/cm^3, μ = 0.92 cm^{-1}, R = 0.0414, $wR2$ = 0.1074, GOF = 0.979.

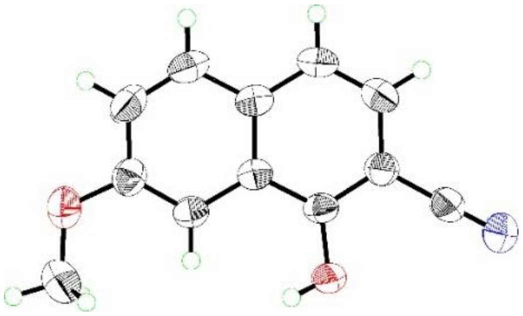

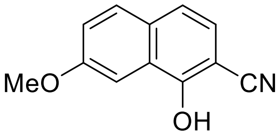

ORTEP (50% Probability)

CV data of 1a–h

Cyclic voltammograms of 3 mM solutions of compounds **1a–h** in 0.03 M Bu_4NClO_4/DMF were recorded at a Pt cathode, 100 mV/s scan rate, and 25 °C.

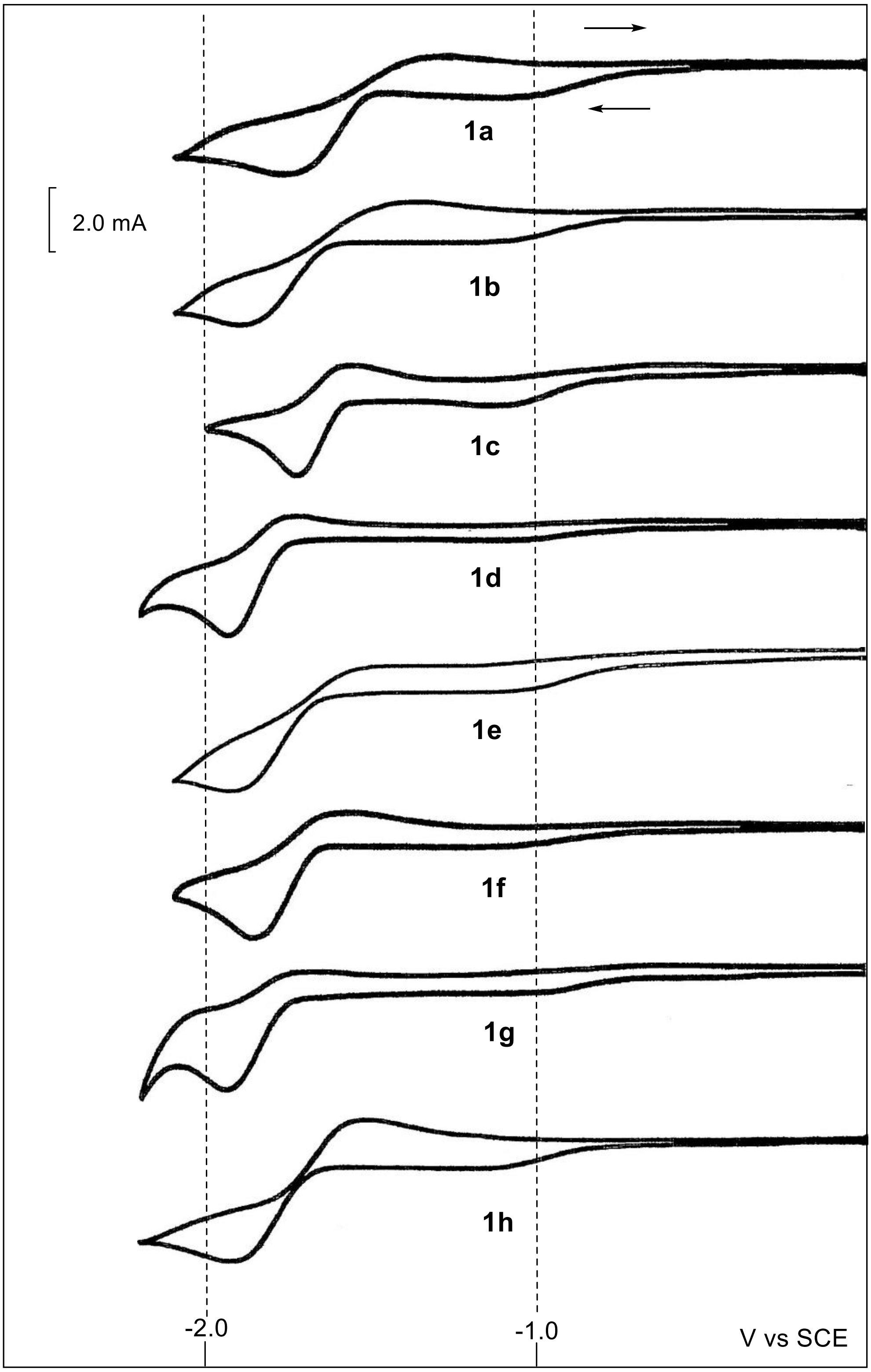

DFT calculations for cyclization of enolate anions

All DFT calculations were carried out with the Gaussian 16[1] program using the supercomputer of ACCMS, Kyoto University. Geometry optimization of intermediates was performed at the B3LYP/6-311+G(2d,p) level of theory using the IEFPCM models for DMF solvent. The optimized geometries were verified by the vibrational analysis (no imaginary frequency) and their energies were thermally corrected to 298 K based on the frequencies. In the calculations of transition states (**TS**) by the same method, it was confirmed that the optimized structures had only one imaginary frequency. The imaginary frequency was verified to be consistent with the corresponding step by displaying the vibrational mode using the Gauss View program.

1) Electroreductive coupling of 1a with 2a

Energy profile (kcal/mol) for the cyclization of Da to Ea calculated at the B3LYP/6-311+G(2d,p)/IEFPCM(THF) level of theory at 298 K

Calculations were made for four possible diastereomers (*RRS*-, *SRS*-, *RRR*-, *SRR*-forms).

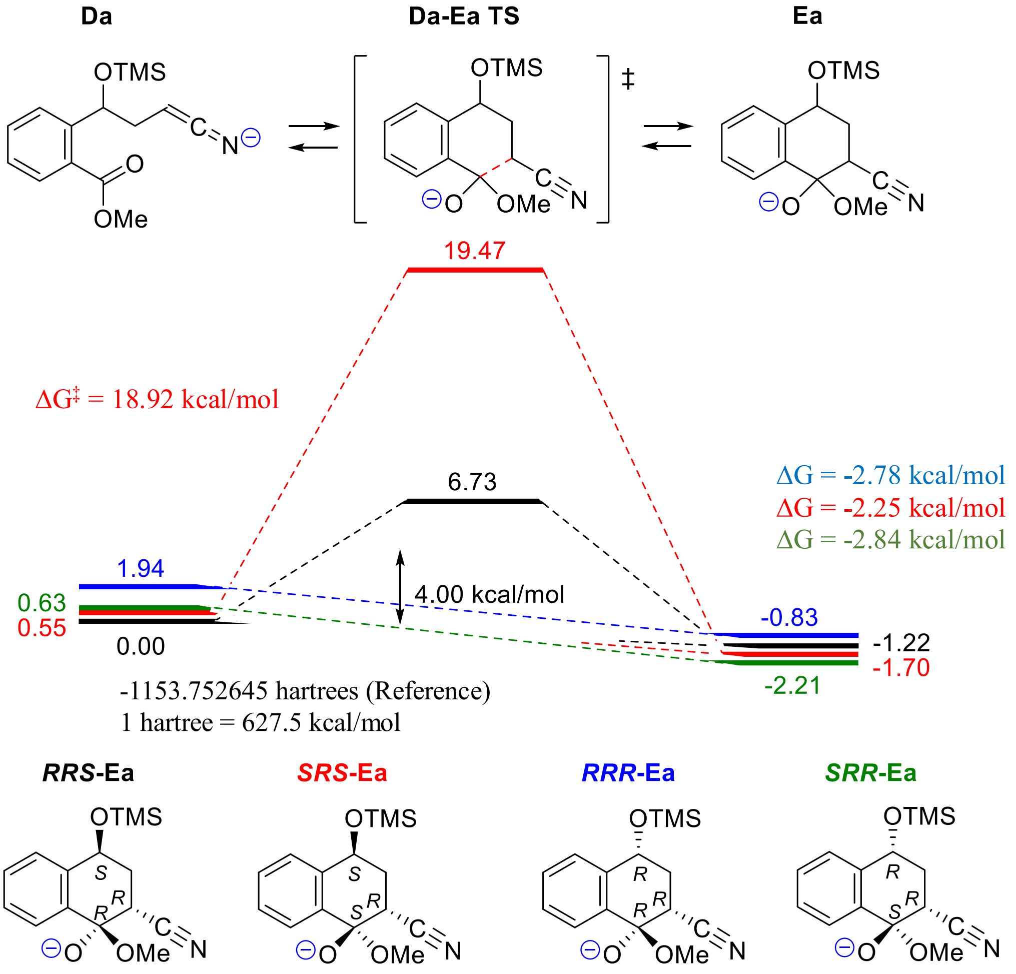

Da-Ea	$\Delta G^{\ddagger}$ (kcal/mol)	ΔG (kcal/mol)	**D**:**E** (calcd)[a]
RRS	6.73	-1.22	11:89
SRS	18.92	-2.25	2:98
RRR	NC[b]	-2.78	1:99
SRR	NC[b]	-2.84	1:99

NC[a]

[a]Calculated from Δ*G* on the basis of the Maxwell–Boltzmann distribution law at 25 °C.
[b]Could not be calculated.

It was found that ***RRS*-Da** was the most stable diastereoisomer and had a small activation energy for the cyclization.

Z-Matrix orientations and thermally corrected free energies

***RRS*-Da**

Z-Matrix orientation:

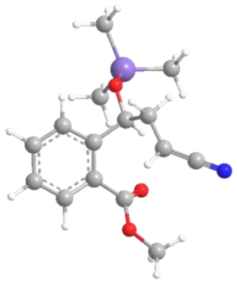

C	-0.22265400	2.27291900	-0.98987600
C	0.52811100	3.44184700	-0.97888300
C	1.78708800	3.45279500	-0.38594300
C	2.27337400	2.29133300	0.19502800
C	1.51185500	1.11441700	0.20753900
C	0.24138800	1.09386100	-0.40158400
C	2.05550700	-0.05071300	0.96878500
C	1.26565700	-1.44344900	-1.93414700
C	-0.14927900	-0.94864500	-1.86011900
C	-0.56730200	-0.17530800	-0.56778800
C	1.62639800	-2.68690500	-1.48519900
N	1.92463600	-3.76882700	-1.10872400
O	1.41094800	-0.77557200	1.69914600
O	-1.96641900	0.13882000	-0.67685100
Si	-3.10539700	-0.27857100	0.46719200
C	-3.17586600	-2.14475000	0.68115400
C	-4.72529600	0.36702600	-0.22227200
C	-2.72213500	0.53051500	2.12079100
O	3.39434900	-0.15300600	0.84537300
C	4.01778000	-1.21118500	1.59804900
H	-1.19534100	2.26111400	-1.46325300
H	0.13261800	4.34121300	-1.43704000
H	2.38344300	4.35725700	-0.37245200
H	3.24868700	2.29033100	0.66312000
H	2.05316000	-0.77262800	-2.25433300
H	-0.85237400	-1.78591600	-1.92866100
H	-0.38323600	-0.28406900	-2.70125900
H	-0.39458100	-0.83050100	0.28392100
H	-3.42283900	-2.63910200	-0.26203900
H	-3.94050200	-2.41709300	1.41484900
H	-2.22288400	-2.54901100	1.03195100
H	-4.69242700	1.45164900	-0.35408800
H	-5.55389700	0.13554900	0.45308100
H	-4.94609700	-0.08517800	-1.19255000
H	-3.48259300	0.26697000	2.86202000
H	-2.70186100	1.61978200	2.03120700
H	-1.75346800	0.20675100	2.50999800
H	5.07976500	-1.13291900	1.38037500
H	3.63273900	-2.17745000	1.27652400
H	3.83600900	-1.07944900	2.66447200

Thermally corrected free energy: **-1153.752645 hartrees**

***SRS*-Da**
Z-Matrix orientation:

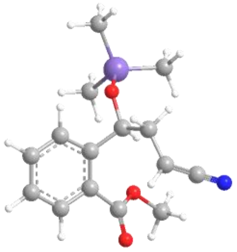

C	-0.02234200	2.40035600	-0.78290600
C	0.76049400	3.54251800	-0.67951800
C	2.03213000	3.46389100	-0.11911200
C	2.49168600	2.24032300	0.34023700
C	1.69761900	1.08690200	0.26627600
C	0.41606100	1.15565900	-0.31944100
C	2.31983200	-0.13284700	0.86931400
C	1.38313200	-1.32887400	-1.98736400
C	-0.00047400	-0.74971900	-1.94538900
C	-0.43771400	-0.06265400	-0.61103000
C	1.63523600	-2.62468000	-1.61900300
N	1.83534500	-3.75031400	-1.31268400
O	1.44959900	-0.90459600	1.54582100
O	-1.81884500	0.31541100	-0.73964500
Si	-3.01003800	-0.15489300	0.32840500
C	-3.15501800	-2.02869700	0.37910000
C	-4.58362200	0.60877800	-0.34600000
C	-2.64738600	0.49100500	2.05721100
O	3.51544500	-0.34925200	0.86193000
C	1.99545800	-2.10180800	2.13653000
H	-1.00216200	2.45927400	-1.23662800
H	0.38101000	4.49049900	-1.04359900
H	2.65665100	4.34546500	-0.03789100
H	3.47784800	2.15824200	0.77860800
H	2.22900300	-0.69146300	-2.21077900
H	-0.74928000	-1.52976700	-2.12057700
H	-0.14577900	-0.00300400	-2.73580900
H	-0.32513000	-0.79161500	0.18816400
H	-3.94586000	-2.33156600	1.07188300
H	-2.22644100	-2.49946100	0.71210900
H	-3.39909500	-2.43166500	-0.60721300
H	-4.51024200	1.69886600	-0.37886900
H	-5.44006000	0.34847000	0.28247500
H	-4.78939200	0.25262300	-1.35863500
H	-3.43838000	0.19327700	2.75199900
H	-2.58452600	1.58224800	2.06531400
H	-1.70285200	0.09754800	2.44199600
H	1.15434900	-2.59228700	2.61978100
H	2.76017200	-1.84825400	2.87001800
H	2.41314400	-2.74016600	1.36050100

Thermally corrected free energy: **-1153.751768 hartrees**

***RRR*-Da**

Z-Matrix orientation:

C	0.60476300	-2.26399100	-0.91667500
C	1.60179500	-3.23006400	-0.93521900
C	2.87520800	-2.92665400	-0.46381800
C	3.12653100	-1.65125900	0.01447100
C	2.12291100	-0.67382300	0.05397400
C	0.82545900	-0.97463400	-0.41965600
C	2.57288600	0.64443100	0.60120500
C	-0.99894000	2.09042100	-2.01977500
C	-0.06276900	0.93079200	-1.82910300
C	-0.28832800	0.05432800	-0.55433100
C	-0.79913500	3.27326500	-1.34993200
N	-0.59997700	4.30291800	-0.80175800
O	1.62196400	1.31906900	1.26439500
O	-1.55955000	-0.59295200	-0.66480500
Si	-2.65619500	-0.74376700	0.58321900
C	-4.04777100	-1.76904000	-0.14400600
C	-3.29735300	0.93256600	1.13833700
C	-1.88394800	-1.62787100	2.05424800
O	3.71185400	1.05445200	0.49693600
C	2.00279800	2.60593600	1.79555700
H	-0.38026300	-2.50147400	-1.29359700
H	1.38408600	-4.21920000	-1.32192800
H	3.66196200	-3.67111300	-0.47177200
H	4.11214900	-1.38886600	0.37600300
H	-2.00868700	1.88630400	-2.35890400
H	0.97568700	1.28040800	-1.79309000
H	-0.12114600	0.24162600	-2.68067800
H	-0.28660400	0.71852800	0.30653800
H	-4.84279100	-1.92181300	0.59139300
H	-3.69012900	-2.75191600	-0.46163800
H	-4.48554800	-1.27166400	-1.01331600
H	-4.02980600	0.81069700	1.94222300
H	-3.78122700	1.45882700	0.31212600
H	-2.49427500	1.57251200	1.51196300
H	-2.61833500	-1.74089500	2.85748900
H	-1.03582800	-1.06876600	2.45840500
H	-1.52836800	-2.62392700	1.77905100
H	2.31418700	3.26859900	0.99002800
H	2.81264000	2.49138100	2.51467900
H	1.11008800	2.99210400	2.27838600

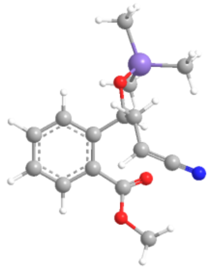

Thermally corrected free energy: **-1153.749548 hartrees**

***SRR*-Da**
Z-Matrix orientation:

C	0.35303200	-2.10616500	-1.29766100
C	1.30305800	-3.10667900	-1.45696700
C	2.56680500	-2.96477700	-0.89185400
C	2.86051000	-1.81625200	-0.17195700
C	1.89958200	-0.81370400	0.01049700
C	0.61819100	-0.94739400	-0.56133300
C	2.27021300	0.34633400	0.87657100
C	-1.08369900	2.31256600	-1.86617800
C	-0.21624200	1.08914600	-1.79097400
C	-0.41724700	0.16386800	-0.54815300
C	-0.68324100	3.51258600	-1.33436300
N	-0.31702800	4.55486300	-0.90804400
O	3.54012200	0.74268000	0.66662400
O	-1.73962500	-0.38496200	-0.59840200
Si	-2.73916200	-0.57251400	0.72377500
C	-4.29860800	-1.34145600	0.02082200
C	-3.12202700	1.08071000	1.52888300
C	-1.96215600	-1.71640400	2.00015800
O	1.55213000	0.86533700	1.70459700
C	4.01281800	1.81913700	1.50189100
H	-0.62582200	-2.21554500	-1.74509900
H	1.05804800	-3.99565800	-2.02691800
H	3.31551900	-3.73853900	-1.01032100
H	3.83906700	-1.69334800	0.27327300
H	-2.14084800	2.18820200	-2.07128300
H	0.84136200	1.37675500	-1.80293700
H	-0.36494900	0.44278400	-2.66665200
H	-0.29919400	0.77065600	0.34740800
H	-5.03736000	-1.50936600	0.80971500
H	-4.08546600	-2.30447000	-0.45037000
H	-4.75167500	-0.69016200	-0.73087000
H	-3.80125900	0.94140700	2.37552300
H	-3.59619000	1.76284000	0.81934400
H	-2.21829300	1.56746300	1.90374300
H	-2.63570800	-1.84948400	2.85226200
H	-1.02236300	-1.30867100	2.38150200
H	-1.75462900	-2.70264900	1.57713800
H	3.97443200	1.52927900	2.55133500
H	3.40700000	2.71043800	1.34665900
H	5.03883300	1.99568900	1.19116800

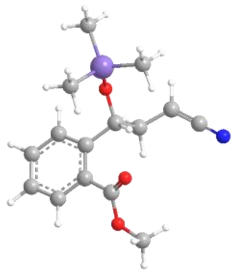

Thermally corrected free energy: **-1153.751646 hartrees**

***RRS*-Da-Ea TS**
Z-Matrix orientation:

C	-0.45530700	2.37552700	-0.60543000
C	0.28143600	3.55790200	-0.60385200
C	1.62730900	3.53058100	-0.25757800
C	2.22645400	2.32287700	0.09020600
C	1.49157600	1.13561100	0.10955800
C	0.13059300	1.16364800	-0.24910800
C	2.12086300	-0.15266900	0.57993800
C	1.52810500	-1.21681500	-1.25135200
C	0.02058000	-1.05445300	-1.40321000
C	-0.62393400	-0.14029800	-0.32950900
C	1.99853800	-2.52434500	-1.07379900
N	2.38199600	-3.60647300	-0.85625800
O	1.66059200	-0.77028500	1.55849300
O	-1.99809900	0.06461900	-0.67042700
Si	-3.26954700	-0.42395600	0.29448100
C	-3.24407200	-2.28881100	0.52864600
C	-4.80338800	0.10348600	-0.64487000
C	-3.19182900	0.42125300	1.97167500
O	3.50624900	-0.10496500	0.37912700
C	4.27299100	-1.01556800	1.16724600
H	-1.49889800	2.38388000	-0.89078000
H	-0.19519000	4.49270700	-0.87688000
H	2.21134100	4.44412700	-0.25423800
H	3.27404400	2.29488200	0.35804200
H	2.12988400	-0.64777800	-1.95284300
H	-0.49005200	-2.01944700	-1.35361100
H	-0.23189500	-0.61700300	-2.37499400
H	-0.53951900	-0.64393500	0.63555200
H	-3.31250300	-2.80738000	-0.43120200
H	-4.08916100	-2.60867200	1.14554000
H	-2.32863200	-2.62036600	1.02561200
H	-4.82245800	1.18641900	-0.79200200
H	-5.70796500	-0.17718100	-0.09796100
H	-4.84404600	-0.37223900	-1.62809800
H	-4.03449900	0.10985200	2.59614900
H	-3.23270000	1.50858700	1.86787400
H	-2.27267200	0.16892000	2.50683800
H	5.31169200	-0.85032500	0.88184000
H	3.99548500	-2.04928900	0.96353000
H	4.14800700	-0.81756400	2.23379600

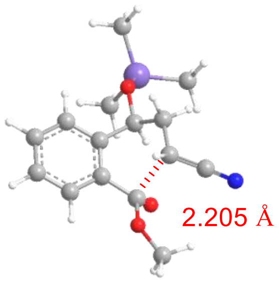

Imaginary frequency: -241.72
Thermally corrected free energy: **-1153.741925 hartrees**

***SRS*-Da-Ea TS**

Z-Matrix orientation:

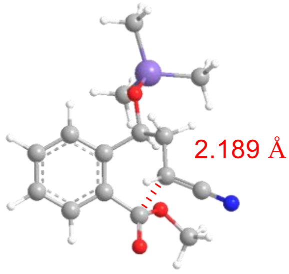

C	-0.43159400	2.39142500	-0.53528500
C	0.25022700	3.60279300	-0.46226200
C	1.59247800	3.61883600	-0.10114500
C	2.23780800	2.42253200	0.18906500
C	1.55841400	1.20529700	0.14144100
C	0.20173700	1.18754600	-0.23244600
C	2.35537900	-0.03831700	0.47007900
C	1.70437300	-1.03767400	-1.36502000
C	0.18794300	-0.99161900	-1.48535500
C	-0.50503100	-0.13784700	-0.39137300
C	2.30127000	-2.30878100	-1.35575100
N	2.80852100	-3.35778800	-1.29380400
O	1.66821100	-0.78821400	1.45001700
O	-1.87906000	0.03478600	-0.75706400
Si	-3.15366300	-0.44760200	0.20084900
C	-3.09980900	-2.30461500	0.48998900
C	-4.68619600	0.02088400	-0.77112500
C	-3.12062200	0.44187800	1.85679600
O	3.59197700	-0.02484000	0.47295300
C	2.45939300	-1.78641200	2.09168500
H	-1.46900600	2.36819200	-0.84118700
H	-0.26534900	4.52763000	-0.69626900
H	2.13474800	4.55625900	-0.04558100
H	3.28657500	2.40440800	0.45702200
H	2.23356300	-0.34984800	-2.01865800
H	-0.24249400	-1.99516300	-1.43312700
H	-0.12684800	-0.56741600	-2.44555100
H	-0.43229700	-0.68019500	0.55225500
H	-3.94547700	-2.62163400	1.10722100
H	-2.18416100	-2.60624300	1.00513600
H	-3.14876800	-2.85188600	-0.45479600
H	-4.72369400	1.09777700	-0.95339800
H	-5.59164100	-0.25882400	-0.22553000
H	-4.70680000	-0.48655000	-1.73871500
H	-3.96209300	0.12459800	2.47952700
H	-3.18763600	1.52467000	1.72533800
H	-2.20111200	0.22832100	2.40791500
H	1.79714300	-2.28211000	2.80177100
H	3.30124300	-1.33843000	2.62387800
H	2.84210100	-2.51200100	1.37449000

Imaginary frequency: -217.89

Thermally corrected free energy: **-1153.721615 hartrees**

***RRS*-Ea**

Z-Matrix orientation:

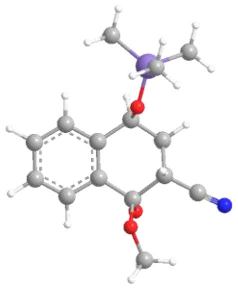

C	0.35957500	2.57448800	-0.46947800
C	-0.42109800	3.67586200	-0.15551300
C	-1.75694000	3.48328200	0.20126900
C	-2.28529500	2.20204300	0.23172700
C	-1.50673400	1.08139300	-0.08114700
C	-0.16503100	1.27626300	-0.43068500
C	-2.20961600	-0.29516800	-0.16367200
C	-1.09124300	-1.41794700	-0.06703800
C	-0.01322400	-1.14831000	-1.12697000
C	0.75587000	0.11688900	-0.76356600
C	-1.66611000	-2.74515600	-0.22923500
N	-2.08465200	-3.81166800	-0.36214000
O	-2.97625200	-0.39770100	-1.20968000
O	1.61066800	-0.14220400	0.37109200
Si	3.26102900	-0.38770600	0.28943900
C	3.68202900	-1.81993600	-0.85529500
C	3.76610100	-0.77618100	2.04990100
C	4.13223000	1.16211100	-0.32492600
O	-2.93311400	-0.39522100	1.17566500
C	-4.18603900	-1.04891600	1.13966600
H	1.39816700	2.71556800	-0.74988000
H	0.00124000	4.67363800	-0.18996500
H	-2.38140400	4.33381700	0.45225400
H	-3.32207400	2.04972900	0.50280800
H	-0.63593600	-1.38509700	0.92609400
H	-0.50353400	-1.01627100	-2.09452600
H	0.68477600	-1.98272400	-1.21185000
H	1.37543000	0.40677600	-1.61991500
H	4.76456300	-1.97759400	-0.87844700
H	3.35635000	-1.62990300	-1.88160500
H	3.21524300	-2.74948000	-0.51973300
H	3.51593100	0.04919000	2.72104900
H	4.84408300	-0.94908300	2.11456100
H	3.25852400	-1.67305600	2.41367300
H	5.21486000	1.00486600	-0.34249600
H	3.92687300	2.01486800	0.32710200
H	3.82414500	1.42951400	-1.33925800
H	-4.77933400	-0.66999900	1.97708600
H	-4.09110400	-2.13755500	1.24756900
H	-4.70590700	-0.84094200	0.20147600

Thermally corrected free energy: **-1153.754587 hartrees**

***SRS*-Ea**

Z-Matrix orientation:

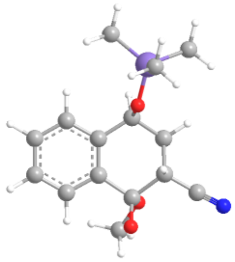

C	0.16203200	2.47539900	-0.48842400
C	-0.62235300	3.55433100	-0.10560800
C	-1.86627600	3.31828000	0.47831500
C	-2.30886800	2.01455800	0.65457000
C	-1.54126000	0.91953000	0.24504200
C	-0.27905900	1.15882900	-0.31630400
C	-2.15223600	-0.49257000	0.43404800
C	-0.99598100	-1.55824400	0.32011200
C	-0.06112500	-1.30261300	-0.87293000
C	0.66096300	0.02791100	-0.69191900
C	-1.55066500	-2.90576200	0.28477500
N	-1.94308800	-3.98941500	0.24455400
O	-2.88643200	-0.75928300	-0.90367100
O	1.65598800	-0.10307400	0.34661200
Si	3.30329200	-0.22243300	0.10017500
C	3.71895700	-1.66645300	-1.03153800
C	4.01550000	-0.49315800	1.81049500
C	3.98036700	1.36153700	-0.65566200
O	-2.90112300	-0.62711200	1.48020000
C	-4.18955700	-0.22853700	-0.96295600
H	1.14039900	2.65261100	-0.92255900
H	-0.26580500	4.56767700	-0.25125500
H	-2.48485900	4.14998000	0.79774700
H	-3.25838600	1.81134400	1.13271400
H	-0.42161400	-1.47564000	1.24715700
H	-0.65368200	-1.27569800	-1.78836800
H	0.67985300	-2.09826200	-0.97149400
H	1.15674300	0.28735800	-1.63427200
H	4.80252700	-1.74102300	-1.16417200
H	3.27452200	-1.54773000	-2.02344900
H	3.36642400	-2.61336900	-0.61491300
H	3.76443900	0.33731400	2.47500400
H	5.10532500	-0.57428900	1.76824500
H	3.62599700	-1.41173300	2.25630400
H	5.06531600	1.28822600	-0.77678700
H	3.77067700	2.22365700	-0.01743900
H	3.55352200	1.55921800	-1.64262400
H	-4.69648800	-0.69823300	-1.81024200
H	-4.18976300	0.85966900	-1.12360100
H	-4.74971000	-0.44358500	-0.04753900

Thermally corrected free energy: **-1153.755361 hartrees**

***RRR*-Ea**

Z-Matrix orientation:

C	-0.52697900	2.36730700	-0.05743800
C	0.19767500	3.54850900	-0.00701000
C	1.58973600	3.49556600	0.07248100
C	2.22767200	2.26549600	0.10572100
C	1.50979200	1.06586200	0.05473900
C	0.11190700	1.12124100	-0.02927800
C	2.29829400	-0.25384200	0.22858300
C	1.39125000	-1.44079200	-0.30216000
C	0.01112500	-1.37576200	0.36788200
C	-0.72815800	-0.14381400	-0.14423500
C	2.04312200	-2.72334200	-0.07381300
N	2.53468900	-3.75290700	0.09322100
O	2.73975500	-0.41667800	1.43701000
O	-1.96407100	0.01246800	0.55688500
Si	-3.47441100	-0.39912900	-0.02826200
C	-4.65394700	0.11596400	1.33160500
C	-3.60169700	-2.24682700	-0.35040400
C	-3.83894300	0.53365100	-1.62012000
O	3.38804500	-0.17002500	-0.85614100
C	4.69662500	-0.50433700	-0.44499500
H	-1.60782300	2.40131900	-0.10815500
H	-0.31570200	4.50321500	-0.02895700
H	2.17038300	4.41086000	0.10925500
H	3.30719000	2.21610600	0.17251100
H	1.27589900	-1.33687000	-1.38619000
H	0.13808200	-1.30388600	1.45007500
H	-0.57883500	-2.26897700	0.15450600
H	-0.93926200	-0.30062100	-1.21288200
H	-5.68771400	-0.10748800	1.05350500
H	-4.58167700	1.18859600	1.52819600
H	-4.43341300	-0.41320700	2.26204700
H	-4.61462000	-2.50435500	-0.67434500
H	-3.38149700	-2.82183700	0.55260000
H	-2.91314100	-2.57216600	-1.13477900
H	-4.83127800	0.26523700	-1.99452000
H	-3.11727800	0.29831100	-2.40683600
H	-3.82262800	1.61484100	-1.46117100
H	5.40006400	0.18788000	-0.92089800
H	4.96648600	-1.52703100	-0.74303900
H	4.79102500	-0.42716300	0.64081800

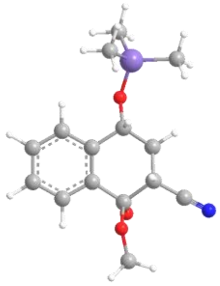

Thermally corrected free energy: **-1153.753974 hartrees**

***SRR*-Ea**
Z-Matrix orientation:

C	-0.35962200	2.35972900	-0.09283700
C	0.41978600	3.50851000	-0.12552600
C	1.80164200	3.39449900	-0.26189500
C	2.38125800	2.13578700	-0.34740600
C	1.61306600	0.96933800	-0.28253000
C	0.21824000	1.08858700	-0.17111200
C	2.37158000	-0.38424700	-0.32586100
C	1.33627700	-1.53015700	-0.64865000
C	0.03559900	-1.41599900	0.16053400
C	-0.68846500	-0.13305800	-0.23046400
C	1.95504100	-2.83951300	-0.47888600
N	2.39778500	-3.89635700	-0.35056200
O	2.65222600	-0.70078400	1.15796200
O	-1.84226700	0.04801500	0.59462900
Si	-3.41256100	-0.28458800	0.12492900
C	-4.44546600	0.11639600	1.63394300
C	-3.61241600	-2.09119200	-0.35596000
C	-3.90367000	0.79793200	-1.33235200
O	3.42644200	-0.39992100	-1.07617900
C	3.81164400	-0.08979700	1.67454200
H	-1.43516800	2.44198300	-0.00021600
H	-0.04766400	4.48436700	-0.05641600
H	2.42168900	4.28331800	-0.30533700
H	3.44997200	2.02799200	-0.48328100
H	1.11116000	-1.42839700	-1.71585000
H	0.27054000	-1.39334200	1.22506300
H	-0.61843800	-2.27025800	-0.02575000
H	-1.01135200	-0.23802900	-1.27756800
H	-5.50588000	-0.06261200	1.43535300
H	-4.32865500	1.16371000	1.92336800
H	-4.15237100	-0.50414900	2.48445900
H	-4.65473200	-2.29758100	-0.61714600
H	-3.33463400	-2.75495200	0.46666000
H	-2.99940400	-2.35233100	-1.22273600
H	-4.93096700	0.57681900	-1.63685400
H	-3.26093100	0.62928000	-2.20051900
H	-3.85143700	1.85916600	-1.07643900
H	4.08000200	-0.61811800	2.59332200
H	3.64761800	0.96896600	1.92319700
H	4.64384800	-0.15647800	0.96707500

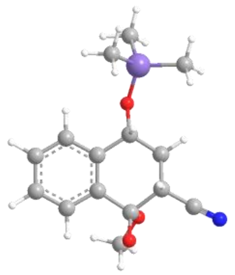

Thermally corrected free energy: **-1153.756165 hartrees**

2) Electroreductive coupling of 1a–h with 2a

Energy profile (kcal/mol) for the cyclization of *RRS*-Dx to *RRS*-Ex calculated at the B3LYP/6-311+G(2d,p)/IEFPCM(THF) level of theory at 298 K

Since ***RRS*-Daa** was the most stable isomer and had a small activation energy for the cyclization as shown in the previous section, we calculated using the RRS form in other cases as well.

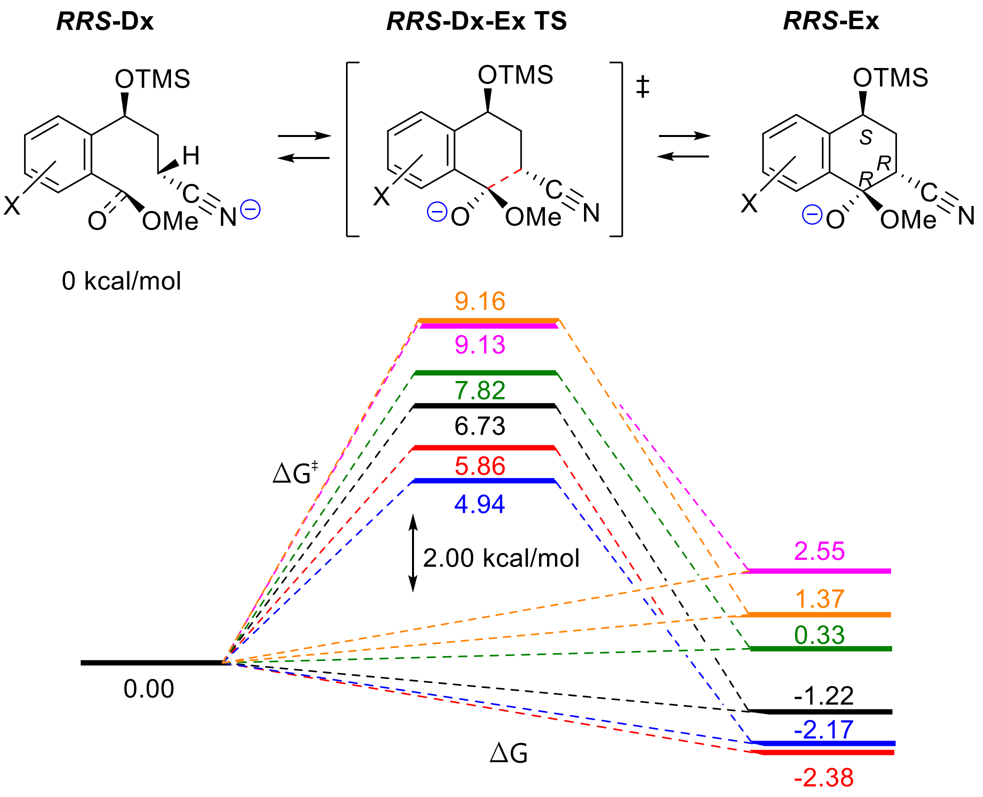

D	X	$\Delta G^{\ddagger}$ (kcal/mol)	ΔG	**D**:**E** (calcd)[a]	**4**:**3** (exptl)[b]
**Da**	H	6.73	-1.22	11:89	<1:99
**Db**	5-MeO	5.86	-2.38	2:98	<1:99
**Dc**	4,5-diMeO	4.94	-2.17	3:97	<1:99
**Dd**	5,6-diMeO	7.82	0.33	36:64	42:58
**De**	6-MeO	9.16	1.37	91:9	>99:1
**Df**	4,5,6-triMeO	9.13	2.55	99:1	>99:1

[a]Calculated from Δ*G* on the basis of the Maxwell–Boltzmann distribution law at 25 °C.
[b]Data from Table 1.

Z-Matrix orientations and thermally corrected free energies

***RRS*-Db**

Z-Matrix orientation:

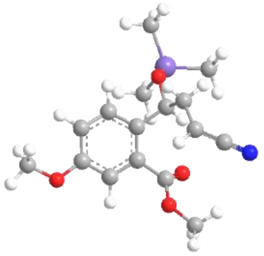

C	-0.67491700	-1.71951100	-0.83696800
C	-1.94882800	-2.26879300	-0.71511500
C	-2.94629800	-1.54099000	-0.07012500
C	-2.64460000	-0.28305100	0.44517600
C	-1.35905300	0.24881800	0.33830300
C	-0.34127500	-0.47067800	-0.32060200
C	-1.09861000	1.55022500	1.02835700
C	0.11067700	2.10916700	-2.01952400
C	1.03081400	0.92348800	-1.94462500
C	1.02591300	0.11417300	-0.61188600
C	0.50070500	3.36233300	-1.62593200
N	0.85039600	4.44508400	-1.29856300
O	-0.11964000	1.79841100	1.70209700
O	2.00432500	-0.93528700	-0.73242900
Si	3.15216100	-1.27233100	0.42889300
C	4.26151600	0.21754800	0.71729000
C	4.13957900	-2.69851600	-0.28363500
C	2.34515900	-1.77409200	2.05176100
O	-2.13367800	2.40258800	0.90787100
C	-1.98489100	3.67338400	1.57164900
O	-4.23298000	-1.97015000	0.10978000
C	-4.59408600	-3.24435600	-0.41569800
H	0.09061700	-2.28336100	-1.35370100
H	-2.14566100	-3.24850100	-1.12763500
H	-3.42435500	0.27405200	0.94685100
H	-0.93502400	1.96005600	-2.25905200
H	2.07109400	1.23490300	-2.09109500
H	0.81219600	0.20187500	-2.74199000
H	1.30708100	0.79399700	0.19048800
H	4.75862400	0.52309800	-0.20704200
H	5.03491000	-0.02024900	1.45383400
H	3.69867700	1.07491900	1.09504400
H	3.50288300	-3.56959500	-0.45915900
H	4.93779300	-2.99849400	0.40129700
H	4.60004700	-2.41938600	-1.23488800
H	3.10660000	-2.01624400	2.79929600
H	1.70847300	-2.65296800	1.92135800
H	1.72742000	-0.96808600	2.45609000
H	-2.91362900	4.20611300	1.38479500
H	-1.14119800	4.21617400	1.14819500
H	-1.83526900	3.53036900	2.64137400
H	-5.64526600	-3.38022300	-0.17171000
H	-4.00806300	-4.04435300	0.04505900
H	-4.46600600	-3.27643600	-1.50108000

Thermally corrected free energy: **-1268.282357 hartrees**

***RRS*-Dc**

Z-Matrix orientation:

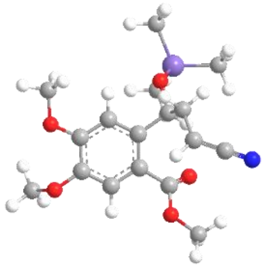

C	-1.04039400	-1.15516800	-0.64689300
C	-2.42764800	-1.16018500	-0.54386500
C	-3.07650600	-0.03915700	0.00663300
C	-2.32205100	1.03587200	0.42898800
C	-0.92112100	1.03517500	0.34591600
C	-0.26755500	-0.07692100	-0.20876500
C	-0.18981800	2.19005300	0.93596100
C	1.14166000	1.92524000	-2.15917100
C	1.53019200	0.49629200	-1.91174800
C	1.21944500	-0.09783300	-0.50006300
C	2.01410400	2.96575100	-1.97751600
N	2.77870300	3.85879900	-1.83472700
O	0.84288300	2.12421700	1.57290300
O	1.71086700	-1.45050800	-0.47742200
Si	2.74220200	-2.04275100	0.69209700
C	4.34611700	-1.06347200	0.71711100
C	3.06256300	-3.81932400	0.18417000
C	1.93835100	-1.97879100	2.39086400
O	-0.85927600	3.34992300	0.76720800
C	-0.25818700	4.51553200	1.36082700
O	-3.22803500	-2.18056500	-0.95325600
O	-4.45232200	0.02440900	0.07222300
C	-2.61538000	-3.32900000	-1.53918900
C	-5.04111600	-0.74296500	1.13139200
H	-0.52493200	-1.99766400	-1.08218500
H	-2.83947400	1.88996200	0.84478200
H	0.10951500	2.16709200	-2.38143800
H	2.60974100	0.36700400	-2.04435200
H	1.05100700	-0.17997000	-2.63178700
H	1.74072800	0.50313400	0.24195200
H	4.84658800	-1.10426500	-0.25383200
H	5.03082200	-1.47109900	1.46682200
H	4.17558300	-0.01221400	0.96261600
H	2.13400400	-4.39552200	0.15863200
H	3.74065500	-4.30724400	0.89015000
H	3.51868200	-3.86820000	-0.80789500
H	2.61472700	-2.38237600	3.15032800
H	1.01698700	-2.56670100	2.41386900
H	1.69137200	-0.95356500	2.67783300
H	-0.91974000	5.34100200	1.11152000
H	0.73309400	4.68091500	0.94194200
H	-0.18401100	4.39915400	2.44199400
H	-3.43152600	-4.00287200	-1.78871700
H	-1.93902500	-3.81849500	-0.83423500
H	-2.06944800	-3.06379100	-2.44760800
H	-6.11803300	-0.60658300	1.04701400
H	-4.70119100	-0.37505200	2.10391700
H	-4.79674800	-1.80214400	1.03124800

Thermally corrected free energy: **-1382.808772 hartrees**

***RRS*-Dd**

Z-Matrix orientation:

C	0.47366100	-1.55829200	1.30991300
C	1.76475500	-2.06365500	1.21215200
C	2.69103500	-1.45592300	0.36889600
C	2.30226800	-0.31798000	-0.35632900
C	0.99950300	0.17546300	-0.24946300
C	0.05540000	-0.44818200	0.58283100
C	0.63070400	1.34569400	-1.11597200
C	-0.80786800	2.01689300	2.37948400
C	-1.58917100	0.75338000	2.13215300
C	-1.36697500	0.05571700	0.76514000
C	-1.28241700	3.24045600	1.97982100
N	-1.71064400	4.29815600	1.66201800
O	-0.18467400	1.29676600	-2.01154100
O	-2.27972200	-1.05936700	0.68668100
Si	-3.24135900	-1.41526700	-0.62865500
C	-4.35552900	0.04214500	-1.04178000
C	-4.26324200	-2.88779800	-0.07530600
C	-2.20339000	-1.85719900	-2.13101600
O	1.31127100	2.45179200	-0.79614300
C	1.02743800	3.62813600	-1.58280400
O	3.96520000	-1.89750100	0.16590300
O	3.16174500	0.24873900	-1.26948400
C	4.24399300	1.01299600	-0.71214900
C	4.39623900	-3.05326800	0.88131700
H	-0.23465100	-2.05359300	1.96134900
H	2.03559800	-2.93977800	1.78456400
H	0.25492900	1.94194800	2.58208800
H	-2.66430200	0.95724600	2.18682700
H	-1.38476700	0.00209600	2.90566300
H	-1.60018600	0.77869600	-0.01799700
H	-4.97240500	0.32238500	-0.18393100
H	-5.02435300	-0.21270200	-1.86937100
H	-3.77964800	0.92081400	-1.34311000
H	-3.62159100	-3.73075500	0.19442400
H	-4.93247500	-3.21791600	-0.87504800
H	-4.87627900	-2.63774300	0.79432500
H	-2.84463300	-2.09524000	-2.98519000
H	-1.57070500	-2.72628900	-1.93173500
H	-1.55589900	-1.02399300	-2.41556500
H	1.71162700	4.38919500	-1.21845600
H	-0.00367700	3.94148800	-1.42725200
H	1.20063400	3.42810300	-2.63929800
H	4.82866700	1.36848000	-1.55894100
H	4.86882300	0.39179600	-0.06949300
H	3.85324600	1.86454800	-0.15174100
H	5.42516100	-3.22456700	0.57375500
H	3.78977500	-3.92592900	0.62542700
H	4.36165100	-2.88658300	1.96109000

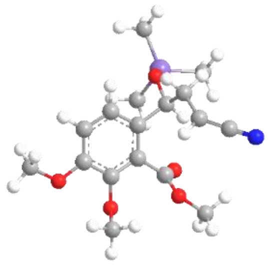

Thermally corrected free energy: **-1382.804271 hartrees**

***RRS*-De**
Z-Matrix orientation:

C	0.14637200	-1.55263100	1.81555500
C	1.22400200	-2.40322200	1.98749600
C	2.35886400	-2.28931200	1.18705200
C	2.40323500	-1.29902500	0.21135400
C	1.31310400	-0.42161600	0.03976100
C	0.17108500	-0.55322000	0.83610600
C	1.39006700	0.59726100	-1.06035000
C	-0.02028800	2.40392500	1.99341300
C	-1.12413000	1.38566500	1.88868800
C	-1.03023600	0.37205500	0.71723800
C	-0.08594500	3.59786100	1.32078300
N	-0.16368300	4.64165200	0.76631800
O	0.68741100	0.58671800	-2.04903700
O	-2.23798400	-0.41388400	0.71876700
Si	-3.12057800	-0.83173500	-0.63393000
C	-3.59084500	0.70285400	-1.61280200
C	-4.65036600	-1.67231600	0.05299800
C	-2.16428000	-2.01341600	-1.73911000
O	2.33291500	1.51818800	-0.83654800
C	2.48757100	2.53577100	-1.84742100
O	3.44710000	-1.13028600	-0.65051400
C	4.56688500	-2.00522100	-0.54362000
H	-0.73481200	-1.65920500	2.43393700
H	1.18959100	-3.17420100	2.74862400
H	3.18510400	-2.97162500	1.32412900
H	0.95687000	2.08232100	2.33701700
H	-2.08921800	1.88756400	1.75988500
H	-1.21141200	0.79526500	2.80955900
H	-0.96406600	0.94162600	-0.21033800
H	-4.14868300	1.41013800	-0.99357100
H	-4.22065600	0.43082300	-2.46512700
H	-2.71144100	1.21950600	-2.00520200
H	-4.38219100	-2.55413700	0.64082200
H	-5.31181700	-1.99622100	-0.75564400
H	-5.21453400	-0.99458300	0.69878900
H	-2.76917800	-2.30186100	-2.60424600
H	-1.89118800	-2.92426500	-1.19968500
H	-1.24700300	-1.54943000	-2.10960800
H	3.30965900	3.15789900	-1.50516700
H	1.57528000	3.12422000	-1.92845700
H	2.72333500	2.08129200	-2.80904600
H	5.26277100	-1.68968000	-1.31738300
H	4.27528700	-3.04437200	-0.71736900
H	5.04606700	-1.91671600	0.43470000

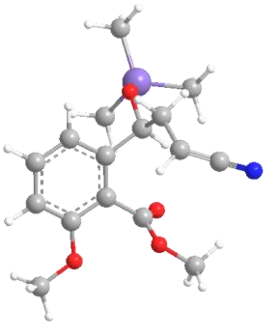

Thermally corrected free energy: **-1268.278265 hartrees**

***RRS*-Df**

Z-Matrix orientation:

C	0.54078300	-1.36598100	0.95897800
C	1.89778600	-1.62797900	0.81742600
C	2.70511300	-0.73685900	0.08907900
C	2.13482000	0.41343100	-0.44936800
C	0.76279100	0.67714300	-0.29853100
C	-0.04403300	-0.22852900	0.39862200
C	0.19336900	1.89518200	-0.95758000
C	-1.23427900	1.80380000	2.50527600
C	-1.81536500	0.47621100	2.10010600
C	-1.52621100	0.00249500	0.64993100
C	-1.92014100	2.97645400	2.31875900
N	-2.52766700	3.98471600	2.18556300
O	-0.66328000	1.87232300	-1.81668300
O	-2.24922800	-1.22549200	0.43739800
Si	-3.10186100	-1.61530200	-0.94300600
C	-4.36193100	-0.27813600	-1.34006500
C	-3.96093500	-3.22769200	-0.51702500
C	-1.95404700	-1.85670300	-2.41197300
O	0.74123800	3.02863800	-0.50050900
C	0.25014900	4.25256400	-1.08438200
O	2.53320100	-2.71686300	1.32945000
O	4.04972300	-0.97545900	-0.06541800
O	2.89676600	1.24668600	-1.23444300
C	3.83659500	2.07355200	-0.52703500
C	4.37171300	-1.90705500	-1.10981300
C	1.76421900	-3.65352000	2.08367000
H	-0.09382100	-2.04973000	1.50237900
H	-0.16679200	1.87663000	2.68126300
H	-2.90696900	0.50012000	2.18593400
H	-1.47678200	-0.32881300	2.76537900
H	-1.89480300	0.76938900	-0.03091800
H	-5.04643900	-0.12038700	-0.50253300
H	-4.95685300	-0.56543900	-2.21222900
H	-3.88345900	0.67744400	-1.56845100
H	-3.23434100	-4.00544900	-0.26740400
H	-4.55747700	-3.58462500	-1.36144100
H	-4.62920800	-3.10241800	0.33879000
H	-2.52507600	-2.14205700	-3.30076700
H	-1.22157000	-2.64406300	-2.21516000
H	-1.41011000	-0.93789000	-2.64383800
H	0.82580300	5.04641400	-0.61672900
H	-0.80924500	4.37140600	-0.86354500
H	0.40632700	4.24806100	-2.16249100
H	4.36692600	2.64426100	-1.28752600
H	4.54339600	1.46245800	0.03521500
H	3.30725500	2.75536900	0.14117800
H	5.45654100	-1.99930500	-1.11370600
H	4.03177800	-1.52982600	-2.07753300
H	3.92181000	-2.88198600	-0.91177000
H	2.46518300	-4.42198500	2.40085500
H	0.97950400	-4.10456300	1.47143700
H	1.31840400	-3.18073000	2.96195300

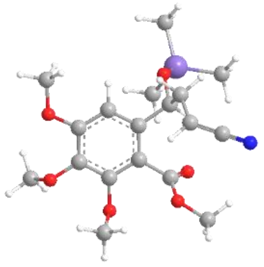

Thermally corrected free energy: **-1497.331304 hartrees**

***RRS*-Db-Eb TS**
Z-Matrix orientation:

C	-0.34881600	-1.95635200	-0.48237400
C	-1.56372900	-2.64432200	-0.43355000
C	-2.71748200	-1.95289100	-0.07942100
C	-2.64332500	-0.59171100	0.22479500
C	-1.42875000	0.08405900	0.19164400
C	-0.25522800	-0.60744600	-0.17365800
C	-1.35156300	1.53139300	0.61374400
C	-0.35990700	2.10641400	-1.27184800
C	0.88723100	1.24242400	-1.41753300
C	1.03401300	0.16346100	-0.31583600
C	-0.15160400	3.48622400	-1.15857600
N	0.02894200	4.62902600	-0.99326600
O	-0.62781600	1.88403100	1.56321100
O	2.12499400	-0.69608300	-0.66424900
Si	3.49579100	-0.86806000	0.27145900
C	4.38687100	0.77713700	0.45217200
C	4.55716000	-2.09394400	-0.66901800
C	3.05951200	-1.53639800	1.97352700
O	-2.59103400	2.15026300	0.41746500
C	-2.79818900	3.36006200	1.14707400
O	-3.96688300	-2.51437200	0.00281300
C	-4.09648600	-3.90108300	-0.28888600
H	0.54742800	-2.48660100	-0.77623000
H	-1.58874900	-3.69742900	-0.67642800
H	-3.54853400	-0.06662000	0.49699100
H	-1.17606300	1.86049500	-1.94385300
H	1.79503100	1.85119800	-1.40125600
H	0.88522900	0.70929100	-2.37438100
H	1.23774500	0.67236300	0.62861700
H	4.67597400	1.17804900	-0.52280600
H	5.29542600	0.65644600	1.04969900
H	3.76118500	1.52233500	0.95021900
H	4.79874900	-1.71899000	-1.66688900
H	4.04458900	-3.05265900	-0.78289400
H	5.49765100	-2.27652600	-0.14147600
H	3.96219300	-1.65769500	2.57978900
H	2.56913300	-2.51052200	1.90054100
H	2.38821000	-0.86167500	2.51101800
H	-3.80747400	3.68586400	0.89657800
H	-2.08123600	4.12686700	0.85488500
H	-2.72104300	3.19076800	2.22268000
H	-5.15070800	-4.13719200	-0.16065200
H	-3.50233900	-4.50974300	0.39885700
H	-3.79838700	-4.12124900	-1.31800100

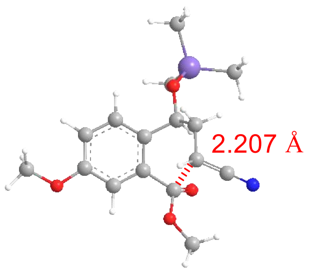

Imaginary frequency: -235.64
Thermally corrected free energy: **-1268.273021 hartrees**

***RRS*-Dc-Ec TS**

Z-Matrix orientation:

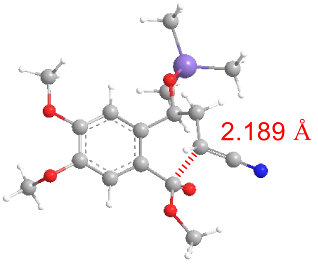

C	-0.75376600	-1.47550600	-0.41445800
C	-2.12252400	-1.73738800	-0.39897300
C	-3.01149800	-0.68853000	-0.11959600
C	-2.51741000	0.58130800	0.13369400
C	-1.14634700	0.84735700	0.13419200
C	-0.25889500	-0.19977400	-0.14825600
C	-0.62309800	2.21262000	0.50410700
C	0.58033700	2.37325100	-1.31686400
C	1.44688500	1.12210100	-1.41303800
C	1.21427800	0.10751400	-0.26451200
C	1.26467600	3.58970600	-1.19002900
N	1.83446900	4.59411300	-1.01214400
O	0.13148600	2.36847800	1.48377900
O	1.97792800	-1.07317900	-0.53034000
Si	3.24872600	-1.58108000	0.42628900
C	4.62531400	-0.30169200	0.43789700
C	3.83264300	-3.17274700	-0.37304400
C	2.66805900	-1.89281400	2.18670800
O	-1.60059600	3.17904500	0.22352200
C	-1.43784900	4.42916100	0.89179300
C	-1.80660500	-4.04507200	-0.95276500
O	-4.37910000	-0.89107300	-0.15177600
C	-4.91425100	-1.53861700	1.00864600
O	-2.67975400	-2.96051700	-0.65287600
H	-0.04527600	-2.25567700	-0.64756700
H	-3.22303100	1.37571700	0.33595900
H	-0.23673000	2.42644600	-2.02937800
H	2.51086800	1.37263500	-1.41152200
H	1.25347500	0.58438100	-2.34709000
H	1.54232000	0.57185300	0.66774300
H	5.00197300	-0.11805300	-0.57165300
H	5.46227800	-0.64642700	1.05249200
H	4.28522100	0.65248700	0.84880900
H	3.03784800	-3.92299700	-0.38546100
H	4.68124800	-3.59182400	0.17503900
H	4.15115000	-3.00010600	-1.40426000
H	3.49940400	-2.24278000	2.80591200
H	1.88290700	-2.65265900	2.21641500
H	2.27362300	-0.98343900	2.64729300
H	-2.27856500	5.04585100	0.57433100
H	-0.50199400	4.90993300	0.60871800
H	-1.45971900	4.30584600	1.97646500
H	-2.44950100	-4.90698100	-1.11795000
H	-1.12826600	-4.25116900	-0.12033800
H	-1.22360700	-3.84777000	-1.85626700
H	-5.98648900	-1.63411200	0.84240700
H	-4.73648800	-0.93260900	1.90218000
H	-4.47550700	-2.52951000	1.14321700

Imaginary frequency: -252.65

Thermally corrected free energy: **-1382.800905 hartrees**

***RRS*-Dd-Ed TS**
Z-Matrix orientation:

C	0.06378300	-1.92955800	-0.61870600
C	-1.12119800	-2.66095300	-0.56065900
C	-2.28524300	-2.03974500	-0.13328600
C	-2.26560400	-0.67096800	0.21360400
C	-1.08037900	0.07015500	0.12349800
C	0.10651300	-0.58927000	-0.27383200
C	-0.99065300	1.53126200	0.54557900
C	0.02870200	2.13400700	-1.27616500
C	1.29101200	1.29902400	-1.43076200
C	1.39784100	0.19489700	-0.35957000
C	0.20407300	3.51845900	-1.14437700
N	0.35019400	4.66328400	-0.96472600
O	-0.31035700	1.83298700	1.54624000
O	2.50579400	-0.65330900	-0.68276800
Si	3.83709300	-0.83879900	0.30616800
C	4.71666500	0.80484700	0.54807300
C	4.93876000	-2.04845100	-0.60892800
C	3.33372200	-1.53271200	1.97901900
O	-2.17603000	2.22301500	0.28111100
C	-2.46163200	3.30808700	1.15973200
O	-3.41807000	-0.14071200	0.75198100
C	-4.42895700	0.21848500	-0.19580500
O	-3.48856100	-2.67549800	0.01296800
C	-3.55368300	-4.06016800	-0.30784700
H	0.97596300	-2.41684500	-0.93431300
H	-1.11553500	-3.70851800	-0.82727900
H	-0.77586100	1.88496200	-1.96136500
H	2.19028200	1.91674200	-1.36345200
H	1.32377600	0.80132700	-2.40630600
H	1.56142600	0.67834900	0.60587700
H	5.04440800	1.22099800	-0.40813100
H	5.60020500	0.67682100	1.18057000
H	4.06915800	1.54119000	1.03124300
H	5.22155400	-1.65712500	-1.58952000
H	4.43281800	-3.00559100	-0.75970600
H	5.85654500	-2.23887900	-0.04538600
H	4.21163800	-1.66054800	2.61939200
H	2.84917200	-2.50666700	1.87292400
H	2.63957200	-0.86710600	2.49845400
H	-3.39351600	3.74263600	0.79763100
H	-1.67465100	4.06136700	1.13893000
H	-2.59513200	2.96122700	2.18753600
H	-5.28930000	0.55043600	0.38468600
H	-4.71479300	-0.64025600	-0.80702400
H	-4.07857700	1.03648600	-0.82760800
H	-4.58245700	-4.36085300	-0.12135400
H	-2.88359200	-4.64772600	0.32603100
H	-3.30829800	-4.23847800	-1.35875300

Imaginary frequency: -252.94
Thermally corrected free energy: **-1382.791813 hartrees**

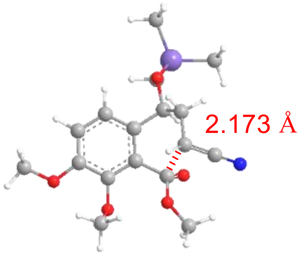

***RRS*-De-Ee TS**
Z-Matrix orientation:

C	0.30606100	-2.16463700	-0.85012800
C	-0.67859800	-3.13893500	-0.89253300
C	-1.97501900	-2.84081400	-0.49266400
C	-2.29704100	-1.54902300	-0.06690500
C	-1.32080400	-0.52606100	-0.07776800
C	-0.00489300	-0.86964900	-0.43539400
C	-1.61458300	0.88772100	0.41037000
C	-0.63135500	1.87200700	-1.24781000
C	0.76570700	1.29986300	-1.43056000
C	1.07043400	0.19637600	-0.40012100
C	-0.71051200	3.22969600	-0.90760800
N	-0.77269000	4.33811800	-0.54541500
O	-1.13044400	1.26610300	1.49660500
O	2.36270300	-0.35316100	-0.67581600
Si	3.64452800	-0.27316900	0.38993700
C	4.10517300	1.51568100	0.73835200
C	5.04478100	-1.15662900	-0.48925100
C	3.22171800	-1.13880900	2.00406200
O	-2.90296900	1.29775300	0.04325500
C	-3.54631900	2.19675000	0.94006000
O	-3.53984500	-1.24081600	0.41224800
C	-4.51949000	-2.26775000	0.48210600
H	1.32342800	-2.39395200	-1.13317000
H	-0.43892600	-4.14465400	-1.21923300
H	-2.72347300	-3.61977400	-0.49559500
H	-1.35199000	1.58856700	-2.00753300
H	1.53788100	2.06724200	-1.33067200
H	0.88670000	0.85837500	-2.42532100
H	1.05733600	0.65365100	0.59221300
H	4.38102700	2.03844900	-0.18115600
H	4.95748900	1.56484300	1.42261500
H	3.27899600	2.06213000	1.20073100
H	4.78465700	-2.19780700	-0.69680300
H	5.95027300	-1.15266000	0.12415400
H	5.28056100	-0.67083400	-1.43957000
H	4.06829000	-1.09261000	2.69561700
H	2.98056000	-2.19150900	1.83556000
H	2.36508000	-0.67305100	2.49811500
H	-4.51524600	2.41716100	0.49139600
H	-2.98195800	3.12161000	1.06144300
H	-3.69656400	1.73988100	1.92223200
H	-5.40692500	-1.79931300	0.90310200
H	-4.19983600	-3.08663000	1.13358100
H	-4.75905400	-2.66532100	-0.50852000

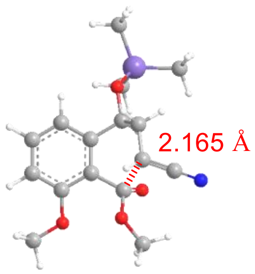

Imaginary frequency: -255.60
Thermally corrected free energy: **-1268.263667 hartrees**

***RRS*-Df-Ef TS**

Z-Matrix orientation:

C	-0.00033200	-1.65801900	-0.52360500
C	-1.23446700	-2.29801000	-0.47866100
C	-2.36901300	-1.56384300	-0.11994900
C	-2.26140200	-0.19574300	0.16100700
C	-1.02352000	0.46812600	0.08327000
C	0.11013000	-0.30127100	-0.23370500
C	-0.84239700	1.93359200	0.45042600
C	0.31605200	2.40995100	-1.31080100
C	1.47610700	1.43169000	-1.42658200
C	1.46344000	0.37428200	-0.30401200
C	0.65846600	3.76043100	-1.15045100
N	0.94518600	4.87364300	-0.94499900
O	-0.20017200	2.23056200	1.47836500
O	2.51024600	-0.57208600	-0.54319500
Si	3.83371100	-0.74597900	0.46031400
C	4.80301300	0.86071500	0.56422100
C	4.86353300	-2.08813700	-0.34659100
C	3.29244700	-1.26678000	2.18331200
O	-1.96642100	2.69072200	0.09605800
C	-2.20467900	3.84683100	0.89385000
O	-3.39622300	0.43465000	0.62204600
C	-4.33572300	0.81553100	-0.38963300
O	-3.59984500	-2.18231100	-0.05935900
C	-3.88499300	-2.79558200	1.20446200
O	-1.43075900	-3.62534800	-0.74118700
C	-0.29468000	-4.41177600	-1.08734000
H	0.89816900	-2.19835500	-0.77702000
H	-0.48250100	2.26724300	-2.03178000
H	2.44107700	1.94343300	-1.38606800
H	1.44678700	0.89071500	-2.37845200
H	1.62840100	0.88948500	0.64497900
H	5.15798200	1.17223800	-0.42154500
H	5.67510100	0.73960400	1.21371800
H	4.19583600	1.67152600	0.97496400
H	5.16545600	-1.79749200	-1.35605200
H	4.30568700	-3.02552200	-0.41698200
H	5.77068500	-2.28033800	0.23331100
H	4.16159400	-1.39018000	2.83627500
H	2.75294000	-2.21705800	2.15697600
H	2.63755500	-0.52146300	2.64195700
H	-3.10700600	4.30432500	0.48732900
H	-1.37824800	4.55483900	0.83501400
H	-2.36928300	3.58096900	1.94082900
H	-5.18967600	1.24643600	0.13221500
H	-4.66150900	-0.05426700	-0.96398900
H	-3.89551300	1.56609200	-1.04832500
H	-4.87405200	-3.24338100	1.11541600
H	-3.89128400	-2.04815200	2.00162000
H	-3.15168100	-3.57329800	1.43230400
H	-0.67103500	-5.41859700	-1.25525900
H	0.43947700	-4.42802000	-0.27727000
H	0.18028200	-4.04401000	-2.00071300

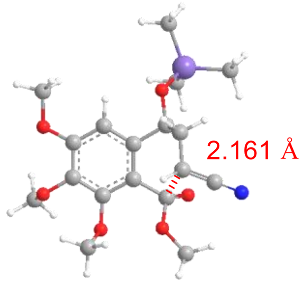

Imaginary frequency: -259.07

Thermally corrected free energy: **-1497.316759 hartrees**

***RRS*-Eb**

Z-Matrix orientation:

C	0.57524900	-2.04803700	-0.63375500
C	1.82017700	-2.60759900	-0.37210300
C	2.86798600	-1.76089300	-0.00273900
C	2.65128300	-0.38931900	0.08433700
C	1.40416400	0.16994600	-0.17948600
C	0.34151500	-0.67657800	-0.53619800
C	1.28892300	1.71605600	-0.20495500
C	-0.24700900	2.08315800	-0.03174000
C	-1.06772500	1.34003200	-1.09671700
C	-1.05676400	-0.15446700	-0.79633600
C	-0.45588000	3.52082100	-0.11687200
N	-0.65859000	4.65403400	-0.18811100
O	1.84328200	2.23965600	-1.25859700
O	-1.88279300	-0.41992400	0.36117100
Si	-3.42668100	-1.05254200	0.31622000
C	-4.57648800	0.01831700	-0.71867600
C	-3.97799800	-1.07541700	2.10588400
C	-3.40845200	-2.79404000	-0.39541400
O	1.91261700	2.13372500	1.12224000
C	2.62223300	3.35650500	1.10439500
O	4.13787600	-2.18980200	0.28921600
C	4.40490100	-3.58646000	0.23533300
H	-0.24036000	-2.70469800	-0.91837100
H	1.95783800	-3.67617400	-0.45852700
H	3.47513300	0.25284500	0.36720400
H	-0.58001600	1.76985700	0.96114600
H	-0.61486700	1.52293600	-2.07417800
H	-2.09914000	1.69471900	-1.12839900
H	-1.47175200	-0.68827500	-1.65881300
H	-5.58783800	-0.39933100	-0.71350300
H	-4.25042700	0.07572700	-1.76074300
H	-4.63257300	1.03689100	-0.32627000
H	-3.30881400	-1.68864700	2.71449000
H	-4.98768700	-1.48606900	2.19562100
H	-3.98638100	-0.06635200	2.52551700
H	-4.41754700	-3.21688500	-0.38789400
H	-2.76326800	-3.45282600	0.19141100
H	-3.05534700	-2.80847700	-1.43004300
H	3.44286800	3.28045600	1.82453300
H	1.98761800	4.20521600	1.39262300
H	3.02945900	3.54998200	0.10925300
H	5.45117000	-3.70199300	0.51050900
H	4.24997600	-3.98402000	-0.77178100
H	3.78106600	-4.13891100	0.94392400

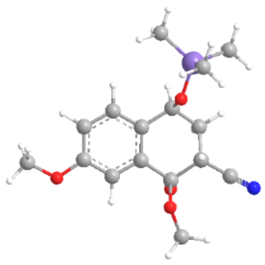

Thermally corrected free energy: **-1268.286151 hartrees**

***RRS*-Ec**
Z-Matrix orientation:

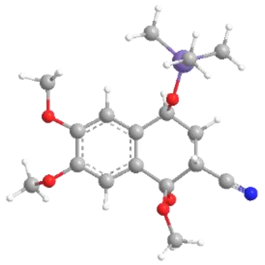

C	0.86590100	-1.57051100	-0.36994300
C	2.19583500	-1.76739900	-0.03163500
C	2.98216400	-0.64323100	0.29002300
C	2.41920300	0.61603300	0.26722500
C	1.07454800	0.82544200	-0.06235500
C	0.29793500	-0.28625900	-0.38395400
C	0.57171400	2.28403000	-0.17793700
C	-1.01453000	2.24858200	-0.10132400
C	-1.55247700	1.25480200	-1.14190600
C	-1.17090200	-0.16663100	-0.74335500
C	-1.57770700	3.57507500	-0.30418100
N	-2.05889600	4.61037800	-0.46816400
O	1.04156900	2.90327400	-1.21992400
O	-1.96747200	-0.58954000	0.38452100
Si	-3.28761300	-1.60868900	0.30021000
C	-4.61014000	-0.92479800	-0.84936500
C	-3.92198500	-1.70331500	2.05914400
C	-2.76968700	-3.31167700	-0.30976400
O	0.98070200	2.89293900	1.16467100
C	1.37297400	4.25000400	1.12255300
O	2.82050100	-2.98358200	0.02313700
O	4.29581100	-0.80252400	0.69051200
C	2.04679300	-4.14322100	-0.26313100
C	5.22438600	-1.00748700	-0.38090700
H	0.24064100	-2.41696800	-0.62060300
H	3.04149300	1.46327500	0.52520100
H	-1.31568900	1.92101200	0.89698800
H	-1.11330100	1.49375800	-2.11348000
H	-2.63744800	1.32245900	-1.23680200
H	-1.37295200	-0.83089000	-1.59160000
H	-5.47514500	-1.59464400	-0.86848300
H	-4.24694700	-0.82852300	-1.87614000
H	-4.95523500	0.05851000	-0.51994000
H	-3.15119500	-2.08549500	2.73309900
H	-4.78774300	-2.36843200	2.12389400
H	-4.22677700	-0.71766700	2.41966900
H	-3.63331000	-3.98293700	-0.33442300
H	-2.01913100	-3.75484500	0.34973200
H	-2.35484900	-3.27576200	-1.32072300
H	2.09462700	4.41383400	1.92876900
H	0.52558300	4.93244700	1.27428300
H	1.83946000	4.48929200	0.16368800
H	2.72203500	-4.98841700	-0.14813200
H	1.66162800	-4.12267000	-1.28659900
H	1.21300600	-4.24627400	0.43690000
H	6.20535000	-1.12299300	0.07817300
H	5.23399900	-0.14221700	-1.05015900
H	4.97771400	-1.90803800	-0.94756700

Thermally corrected free energy: **-1382.812237 hartrees**

***RRS*-Ed**
Z-Matrix orientation:

C	0.10564700	-2.06459200	-0.72453300
C	1.33125700	-2.68685300	-0.53740000
C	2.43858100	-1.90653900	-0.22019500
C	2.30365400	-0.51069000	-0.09038200
C	1.06841800	0.12249100	-0.29636700
C	-0.04517200	-0.68646000	-0.60739600
C	0.97071800	1.67600800	-0.37280300
C	-0.54383900	2.07955400	-0.12066700
C	-1.44672800	1.36015500	-1.12563700
C	-1.44289200	-0.12776200	-0.80716700
C	-0.69407900	3.52524600	-0.19659600
N	-0.85132600	4.66630000	-0.25625400
O	1.45970100	2.13586700	-1.48232100
O	-2.21593700	-0.36691600	0.39276800
Si	-3.77926000	-0.95120900	0.42431000
C	-4.95792300	0.18570100	-0.50204600
C	-4.22185500	-1.01806400	2.24283100
C	-3.86477700	-2.66828800	-0.34092500
O	1.61406500	2.21208700	0.90945000
C	2.59949000	3.20537000	0.72831600
O	3.69787700	-2.40812100	-0.03632800
O	3.44853100	0.20969500	0.16974600
C	3.88433200	0.16020900	1.53174500
C	3.88347500	-3.81381900	-0.15680400
H	-0.75742800	-2.67283000	-0.97199800
H	1.41559900	-3.75829400	-0.65065700
H	-0.82541400	1.78510400	0.89384800
H	-1.05574900	1.52619500	-2.13222400
H	-2.47081100	1.73556100	-1.09360900
H	-1.90732600	-0.66767400	-1.63948500
H	-5.97974500	-0.20123700	-0.44285000
H	-4.69768100	0.26490700	-1.56104300
H	-4.95588300	1.19310600	-0.07809900
H	-5.23677600	-1.40186100	2.38013200
H	-4.17410500	-0.02422400	2.69481000
H	-3.53711600	-1.67119900	2.78949800
H	-4.88713800	-3.05416300	-0.28562100
H	-3.21223500	-3.36934700	0.18567900
H	-3.57470700	-2.66224400	-1.39500700
H	2.22713200	4.18858300	1.04633300
H	2.88982300	3.26612600	-0.32277300
H	3.47993900	2.95920900	1.33199100
H	4.83117800	0.69798900	1.57203900
H	4.04263500	-0.86993200	1.85817200
H	3.15272100	0.65436400	2.17436300
H	4.94076600	-3.99100300	0.02861100
H	3.62833300	-4.16525300	-1.16043900
H	3.28918200	-4.35995200	0.58140700

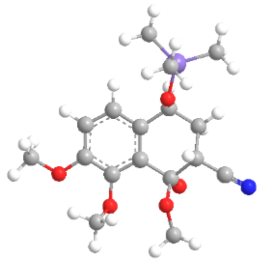

Thermally corrected free energy: **-1382.803743 hartrees**

***RRS*-Ee**

Z-Matrix orientation:

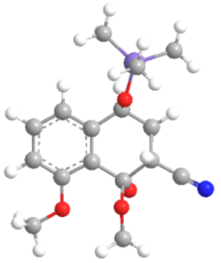

C	-0.21531000	-2.39249900	-0.57802200
C	0.80275300	-3.29298000	-0.34005700
C	2.08415800	-2.82564100	-0.05353300
C	2.33691800	-1.45603000	-0.00819300
C	1.29978400	-0.51196800	-0.22096300
C	0.02471400	-1.01236100	-0.51473600
C	1.61473900	1.01271100	-0.26690600
C	0.24973200	1.79665900	-0.08405400
C	-0.76147600	1.31885900	-1.12713500
C	-1.16777300	-0.10692200	-0.78396800
C	0.49181500	3.22947200	-0.16515400
N	0.65052100	4.37005300	-0.22895200
O	2.28396500	1.35809600	-1.32168400
O	-2.02156400	-0.10323700	0.38053600
Si	-3.68516400	-0.24746700	0.35359100
C	-4.47408400	1.10034900	-0.69548400
C	-4.20041200	-0.07585200	2.14556300
C	-4.19554300	-1.92551500	-0.32656100
O	2.27420600	1.35076800	1.08200800
C	3.58481400	1.86559700	1.02231600
O	3.58604200	-0.96403600	0.23046100
C	4.65007200	-1.87541500	0.45522900
H	-1.21177600	-2.74770600	-0.81409500
H	0.61817000	-4.36027800	-0.38428100
H	2.87843100	-3.53689400	0.12158400
H	-0.14415000	1.59734600	0.91575500
H	-0.29046400	1.34423400	-2.11289700
H	-1.64900400	1.95267300	-1.15849000
H	-1.72093300	-0.52943400	-1.63017200
H	-5.56365100	1.00105100	-0.67741800
H	-4.15655200	1.04156500	-1.74007800
H	-4.22222500	2.09600600	-0.32164100
H	-3.73871900	-0.85087300	2.76239000
H	-5.28571100	-0.16505700	2.24700000
H	-3.90318800	0.89641000	2.54655100
H	-5.28523400	-2.02290700	-0.31143200
H	-3.77689700	-2.73831800	0.27226700
H	-3.86916500	-2.06401700	-1.36078700
H	3.61107200	2.87465600	1.45495600
H	3.92440300	1.91883300	-0.01449100
H	4.27033600	1.22391000	1.58821900
H	5.53371000	-1.26270300	0.62274100
H	4.81918900	-2.52110200	-0.41204100
H	4.47040400	-2.49673400	1.33814000

Thermally corrected free energy: **-1268.276089 hartrees**

***RRS*-Ef**
Z-Matrix orientation:

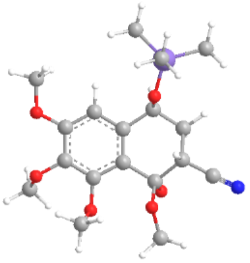

C	0.18671300	-1.82060900	-0.46840500
C	1.45357700	-2.30641000	-0.19004200
C	2.47286900	-1.39129200	0.11072600
C	2.20856000	-0.01975400	0.12374600
C	0.92793600	0.48896400	-0.17662500
C	-0.07540500	-0.44503700	-0.46376900
C	0.70652700	2.01408600	-0.38011100
C	-0.85014800	2.30411000	-0.27269800
C	-1.61436300	1.40526900	-1.24770100
C	-1.50634200	-0.03640000	-0.77258000
C	-1.11052400	3.71507000	-0.51974600
N	-1.35162300	4.82609600	-0.71391300
O	1.24584600	2.44471400	-1.47769400
O	-2.30479500	-0.22383700	0.41637300
Si	-3.82318300	-0.91647900	0.46890400
C	-5.07641800	0.09901100	-0.49855900
C	-4.26289000	-0.94343800	2.28890100
C	-3.78083300	-2.66331200	-0.22930400
O	1.20457700	2.68286400	0.91158000
C	2.07626500	3.77664800	0.73110500
O	1.80809300	-3.62727400	-0.19519500
O	3.73090100	-1.86067800	0.42150400
O	3.27277500	0.81658000	0.37469200
C	3.63720600	0.91571000	1.75603700
C	4.61977900	-1.93398000	-0.70103000
C	0.80181100	-4.58230800	-0.51361600
H	-0.61994700	-2.50201200	-0.70137400
H	-1.18230000	2.09301600	0.74681000
H	-1.16632500	1.50077600	-2.23974300
H	-2.66617900	1.68657500	-1.32175800
H	-1.88363500	-0.69556100	-1.56192900
H	-6.07190900	-0.34683000	-0.41138100
H	-4.82855500	0.14490800	-1.56239900
H	-5.13345800	1.12314000	-0.12088000
H	-3.53467500	-1.52568600	2.85896800
H	-5.24895000	-1.39073200	2.44299500
H	-4.28406200	0.06800000	2.70218600
H	-4.77273700	-3.11966800	-0.15906300
H	-3.08177300	-3.29407100	0.32571500
H	-3.48903400	-2.67819800	-1.28277000
H	2.95311100	3.65750000	1.37778500
H	1.58027300	4.72083600	0.99382700
H	2.40354000	3.83146100	-0.30971500
H	4.52414500	1.54743800	1.79453200
H	3.87406300	-0.06654000	2.17034800
H	2.82750900	1.38330700	2.31939600
H	5.56974100	-2.30647500	-0.31965400
H	4.76309600	-0.94596700	-1.14409200
H	4.23372200	-2.62537600	-1.45486100
H	1.28767800	-5.55445400	-0.46473900
H	0.40698600	-4.42370400	-1.52120200
H	-0.01949200	-4.55056600	0.20767000

Thermally corrected free energy: **-1497.327235 hartrees**

3) Electroreductive coupling of 1a with 2b

Energy profile (kcal/mol) for the cyclization of *RRS*-D'a to *RRS*-E'a calculated at the B3LYP/6-311+G(2d,p)/IEFPCM(THF) level of theory at 298 K

Similarly to the previous section, the calculation was performed on the cyclization of *E*- and *Z*-forms of ***RRS*-D'a**.

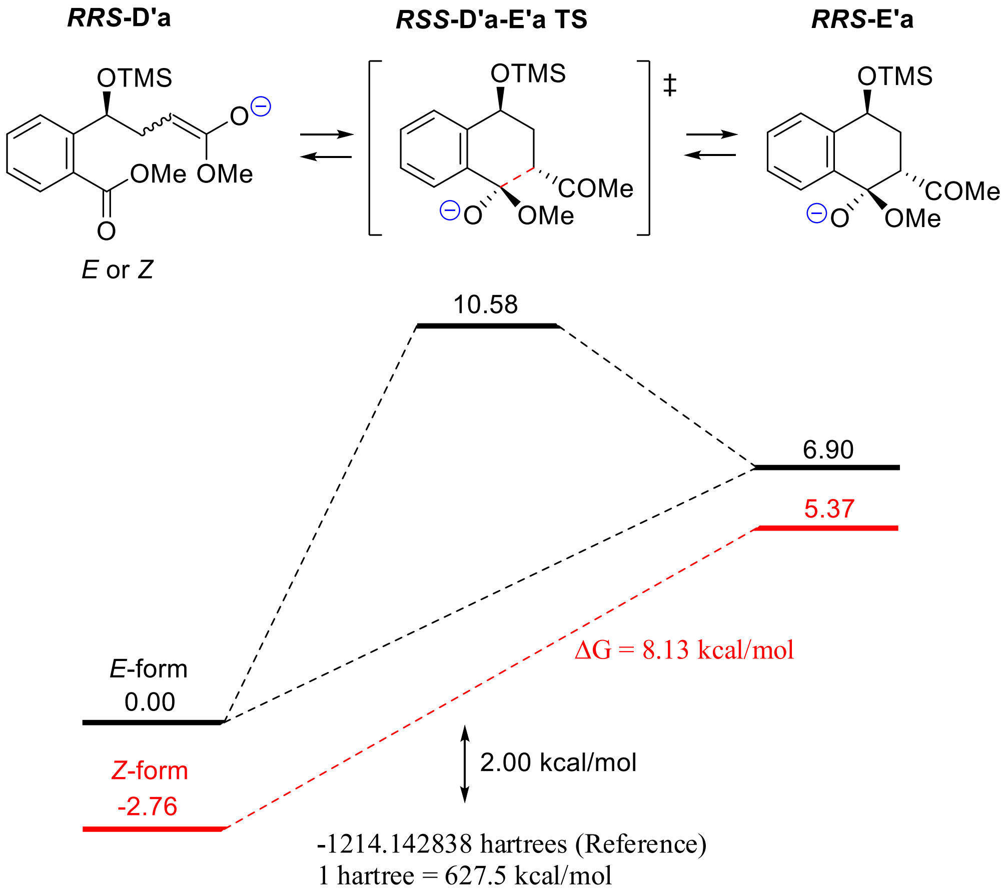

***RRS*-D'a**	$\Delta G^{\ddagger}$ (kcal/mol)	ΔG	**D'a:E'a** (calcd)[a]
E-form	10.58	6.90	100:0
Z-form	NC[b]	8.13	100:0

[a]Calculated from Δ*G* on the basis of the Maxwell–Boltzmann distribution law at 25 °C.
[b]Could not be calculated.

Z-Matrix orientations and thermally corrected free energies

***E*-*RRS*-D'a**

Z-Matrix orientation:

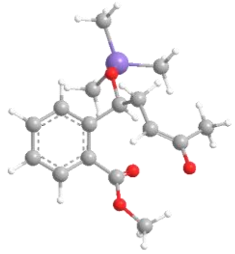

C	-0.64917600	2.15934200	-1.32494800
C	-0.12402100	3.44071500	-1.43236800
C	1.03493100	3.77646700	-0.73923300
C	1.64980900	2.82180400	0.05688900
C	1.11093400	1.53502100	0.18776500
C	-0.05543200	1.18591100	-0.51792700
C	1.77386900	0.60534200	1.15329600
C	1.48137800	-1.28443200	-1.65023200
C	-0.00326400	-1.05910200	-1.70264500
C	-0.61816400	-0.22363400	-0.54291500
C	2.14712700	-2.37376500	-1.13222100
O	3.43171100	-2.51066800	-1.10949300
O	1.19657600	-0.10851000	1.94858200
O	-2.04406100	-0.18491600	-0.73265300
Si	-3.15788800	-0.47752300	0.47370900
C	-2.93749300	-2.21028500	1.17036800
C	-4.82326500	-0.31839100	-0.37297200
C	-2.99814500	0.78137800	1.86097700
O	3.11309700	0.70951600	1.10741000
C	3.84720300	-0.09663100	2.04927300
C	1.37602100	-3.54439000	-0.51805800
H	-1.54306800	1.89515100	-1.87426600
H	-0.61570200	4.17406400	-2.06130200
H	1.45619800	4.77149700	-0.81736400
H	2.55182600	3.07132900	0.59979300
H	2.10852900	-0.49006800	-2.05004000
H	-0.55594500	-2.00237100	-1.70118200
H	-0.28762500	-0.54675400	-2.63107000
H	-0.38125700	-0.72333900	0.39450500
H	-3.04445100	-2.96525700	0.38705300
H	-3.69190700	-2.41121900	1.93690700
H	-1.95572900	-2.34124600	1.63236600
H	-4.95983800	0.68335000	-0.78833500
H	-5.63653200	-0.50243600	0.33485000
H	-4.91872300	-1.03892000	-1.18931200
H	-3.74082200	0.58855200	2.64100700
H	-3.15406900	1.79797700	1.49078900
H	-2.00968300	0.74011400	2.32550800
H	4.89506100	0.10454300	1.84472400
H	3.62618400	-1.15081600	1.89491700
H	3.59435600	0.18880100	3.07035700
H	1.67767900	-4.47191700	-1.01470100
H	0.28977800	-3.45841400	-0.57442200
H	1.65370600	-3.64277900	0.53622000

Thermally corrected free energy: **-1214.142838 hartrees**

***Z*-*RRS*-D'a**

Z-Matrix orientation:

C	-0.65597600	2.04286700	-1.47732400
C	-0.15238800	3.32427200	-1.65980200
C	0.96804900	3.73883400	-0.94595900
C	1.56624400	2.86133900	-0.05442500
C	1.04811800	1.57535000	0.15009900
C	-0.07851700	1.14562200	-0.57503400
C	1.68704100	0.73754200	1.21026400
C	1.50051100	-1.36589100	-1.51700700
C	0.01701800	-1.17039800	-1.60785700
C	-0.61848000	-0.27336700	-0.51366900
C	2.07214300	-2.52670700	-1.04399600
O	1.44344600	-3.57033300	-0.62252400
O	1.09424400	0.07021400	2.03179300
O	-2.04400500	-0.25754600	-0.71786600
Si	-3.17207400	-0.52273700	0.48056200
C	-2.99178300	-2.25180900	1.19278900
C	-4.82645000	-0.33503200	-0.38329400
C	-3.00339600	0.74377900	1.86080400
O	3.02758600	0.87172700	1.21360200
C	3.73139300	0.14989500	2.24225200
C	3.59899500	-2.61409500	-1.00959100
H	-1.52073800	1.71881900	-2.04122100
H	-0.63094100	3.99678500	-2.36253200
H	1.37121600	4.73533200	-1.08059800
H	2.43692600	3.17250500	0.50753100
H	2.15022400	-0.56290000	-1.84797300
H	-0.46957900	-2.14513000	-1.51347200
H	-0.28688100	-0.74406100	-2.57384200
H	-0.38512900	-0.70855700	0.45615300
H	-3.11930300	-3.01125100	0.41710500
H	-3.74578300	-2.42786500	1.96593200
H	-2.00916600	-2.40121300	1.64711700
H	-4.94173300	0.66927400	-0.79914500
H	-5.65025100	-0.50601300	0.31562000
H	-4.92526800	-1.05325800	-1.20128400
H	-3.76891100	0.57993200	2.62534600
H	-3.11939900	1.76198500	1.48031600
H	-2.02730400	0.67400500	2.34743100
H	4.78251200	0.37897000	2.08857000
H	3.55481100	-0.91974800	2.13879200
H	3.40827200	0.47973200	3.22916200
H	3.93551900	-3.46211200	-1.61573300
H	3.93605400	-2.80452800	0.01523100
H	4.08860400	-1.70808500	-1.37592000

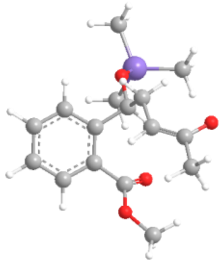

Thermally corrected free energy: **-1214.147237 hartrees**

***E-RRS*-D'a-E'a TS**

Z-Matrix orientation:

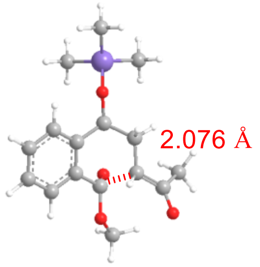

C	-0.86706000	2.43836000	-0.64932800
C	-0.25628100	3.69050600	-0.68641900
C	1.07820900	3.81682700	-0.31867900
C	1.79535800	2.69213900	0.08351500
C	1.18839300	1.43714900	0.13696500
C	-0.16172800	1.31188400	-0.23646000
C	1.93168600	0.21424300	0.63998800
C	1.55652100	-0.88794800	-1.07849800
C	0.04229800	-1.02311000	-1.13871800
C	-0.76966900	-0.06730500	-0.22649300
C	2.38136300	-2.05058100	-0.93070500
O	3.53025900	-2.13806600	-1.41818500
O	1.49679100	-0.40127400	1.64768800
O	-2.12989600	-0.05023100	-0.67467400
Si	-3.41482900	-0.60496700	0.23172000
C	-3.19567300	-2.42497700	0.65079900
C	-4.91118000	-0.35743400	-0.87017400
C	-3.58985600	0.38532300	1.82014000
O	3.31961300	0.45248200	0.53397100
C	4.13920500	-0.30116400	1.41921000
C	1.87325700	-3.19355200	-0.06275100
H	-1.90141400	2.32631700	-0.94653900
H	-0.82153700	4.55941100	-1.00455500
H	1.56365200	4.78621100	-0.34382100
H	2.83644600	2.78295400	0.36213700
H	1.96616100	-0.20325000	-1.81656900
H	-0.27950800	-2.03959200	-0.90524100
H	-0.30259900	-0.82107500	-2.15914600
H	-0.71091300	-0.44582700	0.79583200
H	-3.11701600	-3.03106300	-0.25552800
H	-4.05011500	-2.79088000	1.22793000
H	-2.29656000	-2.59438200	1.24912700
H	-5.03737500	0.69642200	-1.13138300
H	-5.82300500	-0.69003400	-0.36606000
H	-4.81372000	-0.92618700	-1.79837500
H	-4.44561000	0.02856300	2.40109200
H	-3.74516100	1.44622900	1.60783300
H	-2.70131100	0.29593600	2.45055700
H	5.16315400	0.01279500	1.21527600
H	4.05219900	-1.37338900	1.23918900
H	3.89097200	-0.09655500	2.46294500
H	1.12748100	-3.78926500	-0.59831000
H	1.40349700	-2.80392900	0.84444700
H	2.70669700	-3.84532500	0.20032700

Imaginary frequency: -290.58

Thermally corrected free energy: **-1214.125985 hartrees**

***E-RRS*-E'a**

Z-Matrix orientation:

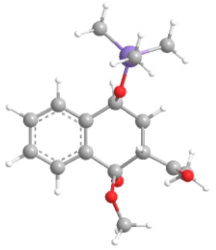

C	0.79466700	2.67695700	-0.49024200
C	0.16142900	3.87087200	-0.18150100
C	-1.18100600	3.84868500	0.20118800
C	-1.86162800	2.64224600	0.25898500
C	-1.23357600	1.42961200	-0.04998600
C	0.11618100	1.45348900	-0.42040900
C	-2.10450700	0.14921600	-0.11810200
C	-1.13070600	-1.10607400	0.03674100
C	-0.04588200	-0.98864900	-1.04471400
C	0.88139500	0.18404000	-0.74286000
C	-1.83573300	-2.43727500	-0.00513500
O	-1.87687200	-3.16285100	0.98274100
O	-2.86492300	0.15269300	-1.18072900
O	1.73962000	-0.13766200	0.37468500
Si	3.34335300	-0.58036800	0.26111900
C	3.56063800	-2.10207900	-0.82445500
C	3.85690300	-0.94883000	2.02388000
C	4.37619600	0.82431400	-0.44731100
O	-2.86890500	0.18794200	1.19775600
C	-4.16717400	-0.36279500	1.14959800
C	-2.48112700	-2.85813100	-1.29964300
H	1.83792600	2.68505100	-0.78863200
H	0.70242200	4.80861100	-0.23929400
H	-1.69180600	4.77270600	0.45018800
H	-2.90412400	2.61882900	0.54832800
H	-0.66212100	-1.04273200	1.01968500
H	-0.51902500	-0.82548900	-2.01655900
H	0.55523300	-1.89908100	-1.11472000
H	1.50577800	0.37557500	-1.62377800
H	4.61559900	-2.38968200	-0.86546000
H	3.22943400	-1.91978400	-1.85041600
H	2.99579600	-2.95210800	-0.43344100
H	3.72294100	-0.07123500	2.66134300
H	4.90960700	-1.24197500	2.06917200
H	3.26107400	-1.76372200	2.44246300
H	5.43103000	0.53594200	-0.48498800
H	4.29549400	1.72300900	0.16952000
H	4.07130400	1.08374200	-1.46468500
H	-4.68165000	-0.04899700	2.06204500
H	-4.16132000	-1.46117000	1.12456400
H	-4.72418900	-0.00626300	0.27916600
H	-3.09153200	-3.74835900	-1.15069100
H	-1.70998800	-3.07183400	-2.04648300
H	-3.06260600	-2.01044800	-1.67482500

Thermally corrected free energy: **-1214.131841 hartrees**

***Z-RRS*-E’a**
Z-Matrix orientation:

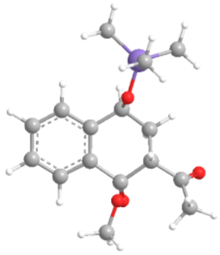

C	0.64425600	2.70936800	-0.58710600
C	-0.09605100	3.85771100	-0.35512400
C	-1.45345000	3.73846500	-0.05096100
C	-2.03825300	2.48357800	0.01955800
C	-1.29605500	1.31237400	-0.18541900
C	0.06160800	1.43804300	-0.50233300
C	-2.04965500	-0.03997800	-0.13349700
C	-1.02149700	-1.24113600	-0.18624500
C	0.06393300	-0.96462000	-1.22084900
C	0.91682400	0.22295000	-0.79543900
C	-1.74525600	-2.54355200	-0.45085600
O	-1.61562600	-3.14783000	-1.50495000
O	-3.02412400	-0.10514200	-0.98708800
O	1.69340600	-0.11317400	0.37792000
Si	3.32425900	-0.45806200	0.38044100
C	3.71768200	-1.91314400	-0.74577700
C	3.71677100	-0.87885100	2.16340300
C	4.32499700	1.03434700	-0.17860200
O	-2.50817200	-0.14803800	1.36847400
C	-3.85108900	0.17935000	1.62269900
C	-2.62794900	-3.10658500	0.63759800
H	1.69481800	2.79086400	-0.84710900
H	0.36979300	4.83422900	-0.42339100
H	-2.05256800	4.62603500	0.12188300
H	-3.09626500	2.39840900	0.22828300
H	-0.56106700	-1.31418300	0.80235200
H	-0.39323400	-0.74484200	-2.18984400
H	0.70181900	-1.83925100	-1.36097400
H	1.60342500	0.48089600	-1.61035300
H	3.17547300	-2.81107900	-0.43859100
H	4.78790100	-2.13890600	-0.71407900
H	3.45625300	-1.70230300	-1.78628700
H	3.49060100	-0.03758400	2.82329900
H	4.77614800	-1.12440600	2.28029500
H	3.13239400	-1.73867600	2.50060300
H	5.39517900	0.81093300	-0.13368100
H	4.13458700	1.89868300	0.46270800
H	4.09301600	1.31965000	-1.20818400
H	-4.25109900	-0.51596300	2.37107200
H	-4.44811300	0.09356700	0.70875200
H	-3.96115600	1.19917100	2.02195000
H	-2.93636800	-4.11825900	0.37443400
H	-3.50453900	-2.46703200	0.75016200
H	-2.11138900	-3.10353400	1.59937700

Thermally corrected free energy: **-1214.134286 hartrees**

References

1. Broom, N. J. P.; Sammes, P. G. *J. Chem. Soc. Perkin 1*, **1981**, 465-470.
2. Zhang, W.; Li, T.; Wang, Q.; Zhao, W. *Adv. Synth. Catal.* **2019**, *361*, 4914-4918.
3. Yeh, C.-H.; Lin, Y.-C.; Mannathan, S.; Hung, K.; Cheng, C.-H. *Adv. Synth. Catal.* **2014**, *356*, 831-842.
4. Liu, T.; Jia, W.; Xi, Q.; Chen, Y.; Wang, X.; Yin, D. *J. Org. Chem.* **2018**, *83*, 1387-1393.
5. Anselmo, M.; Basso, A.; Protti, S.; Ravelli, D. *ACS Catal.* **2019**, *9*, 2493-2500.
6. Cho, I.; Meimetis, L.; Britton, R. *Org. Lett.* **2019**, *11*, 1903-1906.
7. Frisch, M. J.; Trucks, G. W.; Schlegel, H. B.; Scuseria, G. E.; Robb, M. A.; Cheeseman, J. R.; Scalmani, G.; Barone, V.; Petersson, G. A.; Nakatsuji, H.; Caricato, M.; Li, X.; Marenich, A. V.; Bloino, J.; Janesko, B, G.; Gomperts, R.; Mennucci, B.; Hratchian, H. P.; Ortiz, J. V.; Izmaylov, A. F.; Sonnenberg, J. L.; Williams-Young, D.; Ding, F.; Lipparini, F.; Egidi, F.; Goings, J.; Peng, B.; Petrone, A.; Henderson, T.; Ranasinghe, D.; Zakrzewski, V. G.; Gao, J.; Rega, N.; Zheng, G.; Liang, W.; Hada, M.; Ehara, M.; Toyota, K.; Fukuda, R.; Hasegawa, J.; Ishida, M.; Nakajima, T.; Honda, Y.; Kitao, O.; Nakai, H.; Vreven, T.; Throssell, K.; Montgomery, Jr, J. A.; Peralta, J. E.; Ogliaro, F.; Bearpark, M. J.; Heyd, J. J.; Brothers, E. N.; Kudin, K. N.; Staroverov, V. N.; Keith, T. A.; Kobayashi, R.; Normand, J.; Raghavachari, K.; Rendell, A. P.; Burant, J. C.; Iyengar, S. S.; Tomasi, J.; Cossi, M.; Millam, J. M.; Klene, M.; Adamo, C.; Cammi, R.; Ochterski, J. W.; Martin, R. L.; Morokuma, K.; Farkas, O.; Foresman, J. B; Fox, D. J. *Gaussian 16*, *revision C.01*; Gaussian, Inc.: Wallingford, CT, **2019**.
